# The Dry Secretion Metabolome: LC-MS Profiling Distinguishes Subclinical Mastitis from Healthy Udder Quarters Across the Dry Period in Dairy Cows

**DOI:** 10.3390/vetsci13040345

**Published:** 2026-04-02

**Authors:** Barjam Hasanllari, Memet Kaja, Shuang Zhao, Xian Luo, Liang Li, Burim N. Ametaj

**Affiliations:** 1 Department of Agricultural, Food and Nutritional Science, University of Alberta, Edmonton, AB T6G 2P5, Canada; hasanlla@ualberta.ca (B.H.); memet@ualberta.ca (M.K.); 2 The Metabolomics Innovation Centre, Department of Chemistry, University of Alberta, Edmonton, AB T6G 2G2, Canada; szhao1@ualberta.ca (S.Z.); xluo2@ualberta.ca (X.L.); liang.li@ualberta.ca (L.L.)

**Keywords:** dairy cow, dry period, subclinical mastitis, metabolomics, dry secretion, chemical isotope labeling, mammary involution, dipeptides, biomarker, LC–MS

## Abstract

The dry period is a critical window of susceptibility to intramammary infection in dairy cows, yet the metabolic environment of the mammary gland during this phase remains largely uncharacterized. The objective of this study was to profile the metabolome of bovine dry secretion fluid from quarters with subclinical mastitis (SCM; SCC ≥ 200,000 cells/mL) and healthy quarters (H; SCC < 200,000 cells/mL) on day 2 (D2) and day 21 (D21) of the dry period (n = 10 per group quarters per group, drawn from a cohort of 41 enrolled Holstein dairy cows) using high-performance chemical isotope labeling liquid chromatography–mass spectrometry (CIL-LC–MS). A total of 474 metabolites were positively identified. At D2, 186 metabolites differed significantly between SCM and H quarters, with dipeptides dominating the upregulated metabolites, indicating active proteolysis in infected quarters. Norepinephrine was the most significantly depleted metabolite (FC = 0.27, *p* = 3.37 × 10^−7^), pointing to local catecholamine exhaustion. By D21, only 36 metabolites remained altered, representing an 80.6% attenuation of the SCM signature. Interestingly, temporal changes from D2 to D21 far exceeded disease-related differences, with 316 metabolites shifting in both SCM and healthy quarters, establishing mammary involution as the dominant metabolic event during the dry period. Principal component and PLS-DA analyses confirmed that time, not disease status, was the primary driver of metabolic variation. Pathway analysis revealed significant perturbations in amino acid metabolism, glyoxylate and dicarboxylate metabolism, and tryptophan metabolism. These findings provide the first comprehensive metabolomic map of bovine dry secretion, reveal that subclinical mastitis superimposes a proteolytic and neuroimmune disruption onto the involution process, and identify candidate biomarkers for early detection of intramammary infection during the dry period.

## Simple Summary

Mastitis—infection of the mammary gland—is one of the most costly diseases in dairy farming, reducing milk production, increasing treatment costs, and causing premature removal of cows from production. A particularly vulnerable time is the dry period, the six-to-eight weeks between lactations when the mammary gland undergoes internal renewal. Despite this importance, the chemical environment inside the gland during this phase was almost entirely uncharacterized. We collected fluid directly from individual udder quarters at two days and twenty-one days after drying off, from cows with subclinical mastitis—a hidden infection with no outward signs—and from healthy cows. Using an advanced chemical profiling method, we identified and measured 474 small molecules in these samples. Infection caused a striking accumulation of protein-breakdown products and depleted key protective molecules, including a stress-regulating compound called norepinephrine, in the early dry period. Most disease-related changes resolved by day twenty-one, while the gland’s own renewal process drove far larger chemical shifts regardless of health status. These findings deliver the first detailed chemical map of the dry-period mammary gland, identify candidate early-warning markers for hidden infection at dry-off, and open new avenues for interventions beyond conventional antibiotic treatment.

## 1. Introduction

The dry (non-lactating) period is a critical physiological stage in dairy cows during which the mammary gland undergoes involution, tissue repair, and regeneration of secretory epithelium in preparation for the subsequent lactation [1,2]. Spanning approximately 40–60 days, this transitional phase demands substantial metabolic, endocrine, and immunological adjustments in both the cow and the udder [3]. It has been reported that the dry period also represents a window of heightened susceptibility to intramammary infection (IMI): the termination of regular milk removal disrupts the teat canal’s physical barrier, while the accumulating secretion within the gland provides a nutrient-rich medium for bacterial proliferation [4]. Consequently, new IMIs acquired during the dry period account for a considerable proportion of clinical and subclinical mastitis cases detected after calving [4,5].

Subclinical mastitis (SCM) is characterized by elevated somatic cell counts (SCC) and the presence of intramammary pathogens in the absence of visible clinical signs, making it particularly difficult to detect through visual observation [6]. Its global economic burden is considerable: SCM decreases milk yield, lowers milk quality, increases treatment and culling costs, and, together with reproductive disorders, is a leading reason for premature removal of cows from production [7,8]. Subclinical IMIs present at dry-off can persist through the transition period or progress to clinical disease after calving. Previous work from our group has shown that cows subsequently diagnosed with post-partum SCM already exhibit systemic metabolic perturbations—altered serum and urine metabolite profiles—during the dry period [9,10,11], suggesting that the metabolic signature of infection may be detectable before clinical onset and that an early-intervention window may exist.

Although metabolomic investigations of bovine mastitis have expanded rapidly in recent years, most studies have focused on milk during lactation, blood serum, or urine as the sample matrices [11,12,13,14]. Comparatively little attention has been directed toward the dry secretion fluid that accumulates in the mammary gland during the non-lactating period. This fluid is a direct product of the involuting udder and thus reflects the local metabolic and immunological environment of the gland more intimately than systemic biofluids. During mastitis, metabolite profiles in mammary secretions change markedly due to pathogen metabolism, immune cell activity, epithelial cell damage, and increased vascular permeability that allows blood-derived compounds to enter the gland lumen [10,15]. Yet, to our knowledge, no study has systematically characterized the metabolome of bovine dry secretions, nor compared metabolomic signatures between healthy and SCM-affected quarters during the dry-off period.

Metabolomics—the comprehensive profiling of small-molecule metabolites (typically <1500 Da) in biological samples—offers a powerful, hypothesis-generating approach for biomarker discovery and for elucidating the biochemical mechanisms underlying complex diseases [16]. Liquid chromatography–mass spectrometry (LC–MS)-based platforms, particularly when combined with chemical isotope labeling (CIL) for enhanced sensitivity and metabolome coverage, enable the simultaneous detection and quantification of hundreds of metabolites from a single sample [17]. In the context of mastitis, metabolomic studies on lactating milk have identified potential biomarkers linked to amino acid metabolism, glycerophospholipid turnover, and energy metabolism pathways, including lactic acid, pyruvate, and formic acid [12,13,18]. However, whether similar or distinct metabolic signatures characterize the dry secretion fluid of SCM-affected quarters remains unexplored.

A critical knowledge gap therefore exists: while systemic metabolic alterations preceding SCM have been documented in blood and urine, the local metabolic landscape of the mammary gland during the dry period—as reflected in dry secretion fluid—has not been characterized. Understanding how the dry secretion metabolome evolves from the early dry-off phase (when active involution commences) to the later dry period (when the gland approaches a steady-state quiescent phase), and how this trajectory differs between healthy and SCM-affected quarters, could reveal both the pathophysiology of subclinical infection during this vulnerable period and practical biomarkers for early detection.

The objectives of this study were: (1) to characterize and compare the metabolite composition of dry secretions collected on day 2 and day 21 of the dry period from the same udder quarters, capturing metabolic shifts associated with the transition from active involution to the established dry state; (2) to identify metabolites and metabolic pathways that differ between healthy (H) and subclinical mastitis (SCM) quarters at each time point; and (3) to evaluate candidate biomarkers that could enable early, non-invasive detection of SCM during the dry period. We hypothesized that the dry secretion metabolome would differ significantly between H and SCM quarters, reflecting infection-driven metabolic perturbations, and that the metabolite profiles would shift substantially between day 2 and day 21, reflecting the physiological processes of mammary involution and immune adaptation.

## 2. Materials and Methods

### 2.1. Animals, Experimental Design, and Sample Collection

The study was conducted at the Dairy Research and Technology Centre, University of Alberta, Edmonton, AB, Canada. A total of 41 multiparous Holstein dairy cows were enrolled at the time of dry-off. Cows had a mean parity of 2.3 (range: 1–7) and a mean body condition score (BCS) of 3.06 (range: 2.5–3.25; scale 1–5; [19] assessed at dry-off. Inclusion criteria were: multiparous Holstein dairy cows in good health at dry-off (BCS 2.5–3.25), with no clinical signs of systemic disease or acute mastitis, and with complete quarter-level SCC data available from the pre-dry-off milk sample. Cows with quarters that failed aseptic sample collection at either D2 or D21 were excluded from the final metabolomic analysis. All cows received standard blanket dry-cow therapy consisting of intramammary cephapirin benzathine (Cefa-Dri^®^, Boehringer Ingelheim, Burlington, ON, Canada) and an internal teat sealant (Orbeseal^®^, Zoetis, Kalamazoo, MI, USA) at the time of dry-off. Given that cephapirin benzathine persists in the mammary gland for up to 7–10 days post-treatment, samples collected on D2 reflect an early post-treatment environment, whereas those from D21 represent endogenous metabolite profiles with minimal antibiotic influence. Cephapirin benzathine does not contain amine or phenol functional groups and is therefore not reactive with the dansyl chloride CIL reagent; accordingly, no cephapirin-derived ion features are expected to be detected by the HP-CIL LC–MS platform, and no antibiotic-related peaks were identified in the annotated metabolite list.

Three sample types were collected from each udder quarter: (1) milk, collected two days before dry-off (day −2); (2) dry secretion fluid, collected on day 2 of the dry period; and (3) dry secretion fluid, collected on day 21 of the dry period. Prior to each collection, teats were cleaned with soap and water, dried with single-use paper towels, and disinfected with 70% ethanol. The first few streams of milk or secretion were discarded to minimize teat canal contamination, and samples were then collected aseptically into sterile tubes. Following collection, teats were dipped in an iodine-based teat disinfectant. All samples were transported on ice to the laboratory and stored at −80 °C within 30 min of collection until analysis.

All animal procedures were approved by the University of Alberta’s Animal Policy and Welfare Committee for Livestock (Protocol No. 2021F090R), and animal handling and care followed the standards established by the Canadian Council on Animal Care (CCAC) [20].

### 2.2. Subclinical Mastitis Classification and Study Groups

Milk samples collected at day −2 relative to dry-off were analysed by Lactanet (Edmonton, AB, Canada) for somatic cell count (SCC), total protein, total lipids, lactose, milk urea nitrogen (MUN), and total solids. SCC was determined using a fluorooptic automated cell counter (Fossomatic series, Foss), a validated instrument operating under IDF-aligned accreditation standards and internationally accepted as equivalent to the IDF reference microscopic method for routine diagnostic classification. Individual udder quarters were classified as subclinical mastitis (SCM) or healthy (H) based on SCC: quarters with SCC ≥ 200,000 cells/mL were classified as SCM, and those with SCC < 200,000 cells/mL were classified as healthy [21,22]. Of the 41 enrolled cows, 15 had at least one quarter with elevated SCC (≥200,000 cells/mL), while 26 cows had normal SCC (< 200,000 cells/mL) in all quarters.

From the qualifying pool, a subset of 40 dry secretion samples was selected for metabolomic analysis, comprising 10 samples per group: SCM-D2, SCM-D21, H-D2, and H-D21. The selection within each health category was performed without additional stratification criteria beyond SCC classification. Where more than 10 quarters qualified per category, samples were selected using a computer-generated randomization sequence, thereby avoiding subjective allocation bias. To ensure statistical independence of observations, no single cow contributed more than one quarter to any experimental group; where a cow had two qualifying quarters in the same SCC category, one was selected at random. Once healthy and SCM status was determined based on SCC in the pre-dry-off milk samples, the same udder quarters were consistently sampled for dry secretions on D2 and D21, yielding paired longitudinal observations within each health category. This approach ensured that within-quarter comparisons were consistent across all time points. The quarters were selected from the same cohort of 41 cows to increase statistical power for untargeted metabolomics.

### 2.3. Metabolite Extraction and Chemical Isotope Labeling

Dry secretion samples were thawed at room temperature and processed in randomized order to minimize technical variation from sample handling and instrument drift. Metabolites were extracted using a methanol-based protein precipitation protocol. Briefly, 300 µL of methanol was added to 100 µL of dry secretion in a microcentrifuge tube. Samples were vortexed, incubated at −20 °C for 15 min, and centrifuged at 15,000× *g* for 10 min. The supernatant (350 µL) was transferred to a new tube, to which 50 µL of water and 500 µL of dichloromethane were added. After vortexing and allowing phase separation at room temperature for 10 min, 350 µL of the aqueous (upper) phase was collected and dried under vacuum.

Total metabolite concentrations were determined using the NovaMT Sample Normalization Kit (NovaMT Inc., Edmonton, AB, Canada). Samples were normalized to a total concentration of 2 mM prior to labeling; samples with concentrations below 2 mM were used undiluted. Each normalized sample was divided into three aliquots: one for isotope labeling, one as a backup, and one for inclusion in a pooled quality control (QC) reference. The pooled QC was prepared by combining equal volumes from all individual samples with sufficient post-normalization volume.

Chemical isotope labeling (CIL) targeting amine and phenol functional groups was performed according to the manufacturer’s standard operating procedure (Catalog No. NMT-4101-KT, Nova Medical Testing Inc, Edmonton, AB, Canada). For each sample aliquot, 12.5 µL of buffer reagent (Reagent A) and 37.5 µL of labeling reagent (Reagent B) were added. Individual samples and the pooled QC were labeled with ^12^C_2_-reagent, whereas the pooled reference sample was labeled with ^13^C_2_-reagent. Reaction mixtures were vortexed, briefly centrifuged, and incubated at 40 °C for 45 min. Reactions were quenched with 7.5 µL of quenching reagent (Reagent C; 40 °C, 10 min), followed by pH adjustment with 30 µL of Reagent D. Each ^12^C_2_-labeled sample was then mixed with an equal volume of the ^13^C_2_-labeled reference for LC–MS analysis. A QC injection was prepared by combining equal volumes of ^12^C_2_- and ^13^C_2_-labeled pooled reference to monitor instrument performance.

### 2.4. LC–MS Analysis

Metabolomic profiling was performed on the high-performance chemical isotope labeling (HP-CIL) LC–MS platform [23] using an Agilent 1290 Infinity II UHPLC system coupled to an Agilent 6546 quadrupole time-of-flight (QTOF) mass spectrometer (Agilent Technologies, Santa Clara, CA, USA). Chromatographic separation was achieved on an Agilent Eclipse Plus C18 reversed-phase column (150 × 2.1 mm, 1.8 µm particle size) maintained at 40 °C. Mobile phases consisted of (A) 0.1% (*v*/*v*) formic acid in water and (B) 0.1% (*v*/*v*) formic acid in acetonitrile. The gradient program was: 25% B at 0 min, linear ramp to 99% B at 10 min, hold at 99% B until 15 min, return to 25% B at 15.1 min, and re-equilibration for 3 min (total run time: 18 min). The flow rate was 400 µL/min. Mass spectra were acquired in positive electrospray ionization (+ESI) mode over an *m*/*z* range of 220–1000 at an acquisition rate of 1 Hz.

Pooled QC samples were injected at the beginning and end of the analytical batch to monitor retention time stability, mass accuracy, and signal intensity drift.

### 2.5. Metabolite Identification and Data Preprocessing

#### 2.5.1. Peak Detection, Alignment, and Quantification

Raw LC–MS data files were processed using IsoMS Pro for CIL LC-MS (NMT-5101-SW) version 1.2.14 (Nova Medical Testing Inc., Edmonton, AB, Canada; www.novamt.com (accessed on 15 January 2026)). IsoMS Pro is a dedicated software platform for CIL LC–MS metabolomics that integrates peak pair detection, quantification, filtering, alignment, and imputation into a single automated pipeline [24]. Processing consisted of three sequential steps. First, the IsoMS algorithm detected co-eluting ^12^C/^13^C-labelled peak pairs exhibiting the expected mass difference of 2.0067 Da, while filtering out redundant pairs (adduct ions, dimers, and singlet peaks) so that only the protonated ion of each ^12^C_2_/^13^C_2_-labelled metabolite pair was retained [24]. Second, the Zero-fill program retrieved missing peak pairs from the raw spectra when a metabolite was detected in some but not all runs, based on the expected retention time and accurate mass of the missing pair [25]. Third, IsoMS-Quant reconstructed the chromatographic peaks of each light- and heavy-labelled pair and calculated the peak area ratio (^12^C_2_-analyte/^13^C_2_-reference) to produce the metabolite–intensity table for relative quantification [25]. Multiple peak pairs of the same metabolite across different runs were aligned according to retention time and accurate mass. Finally, missing values were imputed using a dedicated scenario-based imputation function embedded in the software to generate the final complete data table.

#### 2.5.2. Metabolite Identification

Metabolite identification followed the three-tiered approach established for the CIL LC–MS platform using the NovaMT Metabolite Databases v2.0 (Nova Medical Testing Inc.,Windsor, ON, Canada):

Tier 1 (positive identification): Peak pairs were matched against the CIL Library of individually labelled authentic standards (≥1500 metabolites) based on accurate mass (≤5 ppm), retention time (within calibrated window), and characteristic ^12^C/^13^C peak pair pattern. Tier 1 identifications correspond to Metabolomics Standards Initiative (MSI) Level 1 [26].

Tier 2 (high-confidence putative identification): Remaining peak pairs were matched against the Linked Identity (LI) Library, containing >9000 pathway-related metabolites with computationally predicted retention times. Matches were based on accurate mass and predicted retention time. Tier 2 identifications have been reported to be approximately 90% accurate [27] and correspond to MSI Level 2.

Tier 3 (mass-matched identification): Any remaining peak pairs were searched against the MyCompoundID (MCID) database (www.mycompoundid.org), which comprises a zero-reaction library of 8021 known human endogenous metabolites, a one-reaction library of 375,809 predicted metabolic products, and a two-reaction library of 10,583,901 predicted compounds [28]. Matches were based on accurate mass only and correspond to MSI Level 3.

A total of 474 metabolites were confidently annotated across the four experimental groups (SCM-D2, H-D2, SCM-D21, H-D21). Of the 474 metabolites, 228 were identified at Tier 1 (CIL Library; matched by accurate mass and retention time) and 246 were identified at Tier 2 (LI Library; matched by accurate mass and predicted retention time). Tier 3 mass-matched features were excluded from downstream analysis. Relative quantification was based on the peak area ratio of each ^12^C_2_-labelled analyte to its corresponding ^13^C_2_-labelled pooled reference sample, ensuring that all intensity values are internally normalized for ionisation efficiency, matrix effects, and instrument drift [29].

#### 2.5.3. Data Filtering and Quality Assessment

The final dataset comprised 474 metabolites × 40 biological samples (18,960 data points) with zero missing values. Complete detection across all samples was achieved because the CIL LC–MS platform detects metabolites as co-eluting ^12^C/^13^C peak pairs, and the Zero-fill algorithm retrieves any pairs initially missed during primary peak picking. No imputation or minimum-value substitution was required.

**Figure 1:**
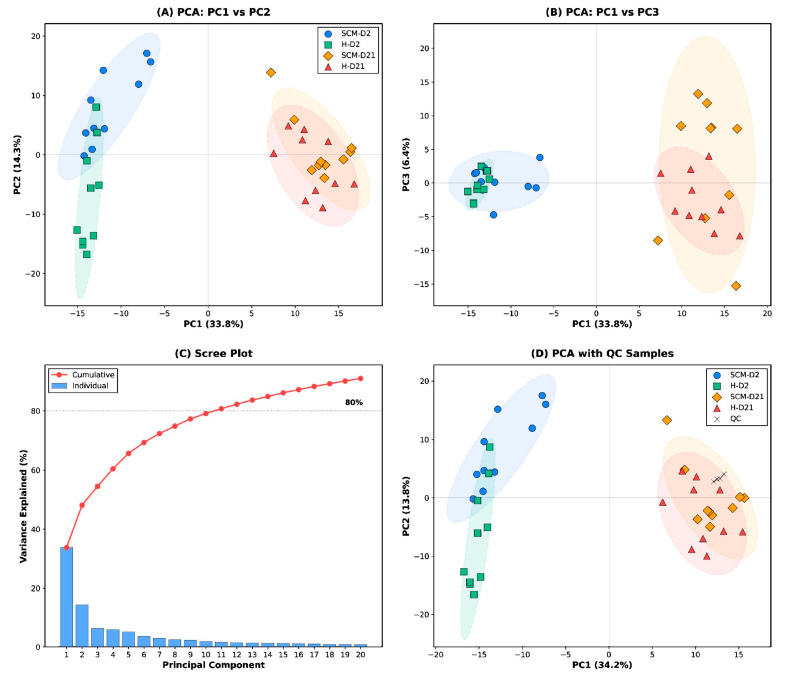
Principal component analysis (PCA) of bovine dry secretion metabolomes (n = 10 per group; 474 metabolites; CIL-LC-MS). (**A**) Score plot of the first two principal components (PC1: 33.8%; PC2: 14.3% of total variance) with 95% confidence ellipses. The dominant separation along PC1 is temporal (Day 2 vs. Day 21), reflecting the large-scale metabolic remodeling of mammary involution. Health-status separation (SCM vs. H) is secondary and most evident on Day 2. (**B**) Score plot of PC1 vs. PC3 (6.4%), providing an alternative view of group clustering. (**C**) Scree plot showing individual (bars) and cumulative (line) variance explained by the first 20 principal components. Five components are required to capture >65% of total variance, consistent with the high dimensionality of the metabolome. (**D**) PCA including quality control (QC) samples (×). The tight clustering of QC samples near the origin confirms analytical reproducibility and instrument stability across the run. Data were auto-scaled (unit variance) prior to decomposition. No group labels were used in the PCA model (unsupervised analysis).

Analytical reproducibility was assessed using pooled QC samples prepared by combining equal-volume aliquots from all individual samples. QC samples were injected at the beginning and end of the analytical batch. QC samples clustered tightly near the origin in PCA score plots (Figure 1D), indicating minimal instrument drift and batch effects over the analytical run. All 474 detected metabolites were retained for statistical analysis without CV-based filtering, as analytical reproducibility was confirmed by PCA-based QC assessment.

#### 2.5.4. Data Preprocessing for Statistical Analysis

The metabolite–intensity table (474 metabolites × 40 samples) was exported from IsoMS Pro as a .csv file formatted for MetaboAnalyst 6.0 [27]; www.metaboanalyst.ca. The file structure consisted of metabolite identifiers in rows and sample identifiers in columns, with a condition label row assigning each sample to one of four groups (SCM-D2, SCM-D21, H-D2, H-D21; n = 10 per group). QC samples were excluded from inferential statistical analyses. Four pairwise .csv files were additionally prepared for the two-group comparisons (20 samples each).

Within MetaboAnalyst, the following preprocessing steps were applied prior to multivariate and univariate analyses: Data were auto-scaled (mean-centred and divided by the standard deviation of each variable) prior to multivariate analyses (PCA and PLS-DA), giving equal weight to all metabolites regardless of absolute abundance.

For univariate analysis (*t*-tests, fold-change calculations), MetaboAnalyst operates on the normalized and transformed (if applicable) data prior to scaling. Fold changes were calculated as the ratio of group means from the unscaled concentration table, ensuring that FC values reflect biologically interpretable ratios rather than standardised units.

### 2.6. Statistical Analysis

#### 2.6.1. Multivariate Analyses

Principal component analysis (PCA) was performed on the auto-scaled data matrix as an unsupervised method to assess overall metabolomic variation, detect outliers, and evaluate batch effects. PCA was computed using singular value decomposition. Explained variance was reported for each component; 95% confidence ellipses were drawn assuming a bivariate normal distribution for each group. Analytical reproducibility was confirmed by tight clustering of QC samples near the origin of the PCA score plot.

Partial least squares–discriminant analysis (PLS-DA) was applied as a supervised method to maximise group separation and identify discriminatory metabolites. Five PLS-DA models were constructed: one four-group model (3 components, dummy-encoded response matrix) and four pairwise models (2 components each, binary encoding): (i) SCM-D2 vs. H-D2, (ii) SCM-D21 vs. H-D21, (iii) SCM-D2 vs. SCM-D21 (SCM temporal), and (iv) H-D2 vs. H-D21 (H temporal). Model quality was assessed by the coefficient of determination R^2^Y (goodness of fit) and the predictive ability Q^2^, estimated by leave-one-out cross-validation (LOOCV). LOOCV was chosen over k-fold cross-validation due to the small per-group sample size (n = 10), following recommendations for metabolomics studies with limited samples [30]. Classification accuracy was calculated as the proportion of correctly classified samples during LOOCV.

Model robustness was assessed by permutation testing (200 permutations per model). In each permutation, group labels were randomly reassigned while the data matrix was held constant, and R^2^ and Q^2^ were recalculated. Empirical *p*-values were computed as (r + 1)/(n_perm_ + 1), where r is the number of permuted values exceeding the observed statistic, following [31]. A *p*-value < 0.05 was considered evidence that the observed separation was not attributable to chance.

To statistically test group separation in multivariate space, permutational multivariate analysis of variance (PERMANOVA) was applied to both PCA and PLS-DA score matrices using Euclidean distance with 999 permutations.

#### 2.6.2. Variable Importance in Projection (VIP)

VIP scores were extracted from each PLS-DA model to rank metabolites by their contribution to group discrimination. The VIP score for the jth variable was calculated as:

VIP_j_ = √(*p* × Σ_a_[SS_a_ × (w_ja_)^2^]/Σ_a_SS_a_)

where *p* is the total number of variables, SS_a_ is the sum of squares explained by component a, and w_ja_ is the weight of variable j on component a. Metabolites with VIP > 1 were considered important contributors to group separation, as a VIP of 1 corresponds to the expected value when all variables contribute equally [32]. The top 20 VIP-ranked metabolites for each pairwise comparison are reported in Supplementary Table S3A–D.

#### 2.6.3. ROC Curve Analysis

Receiver operating characteristic (ROC) curves were constructed for each pairwise comparison to evaluate diagnostic performance. Cross-validated predicted scores from LOOCV were used as the continuous classifier output to avoid overfitting bias. The area under the ROC curve (AUC) was calculated using the trapezoidal rule. ROC curves were generated for models with 1, 2, and 3 PLS components using all 474 metabolites, and additionally for a reduced model using only the top 20 VIP-ranked metabolites (2 components) to evaluate whether a compact biomarker panel retained discriminatory power.

#### 2.6.4. Univariate Statistical Analysis

For each pairwise comparison, fold changes (FC) were calculated as the ratio of group means (test group/reference group). Statistical significance was assessed using Welch’s two-tailed *t*-test (unequal variances). Metabolites were considered significantly altered when meeting dual criteria of *p* < 0.05 and FC > 1.5 (or <1/1.5). Results were visualised as volcano plots with –log_10_(p) plotted against log_2_(FC).

No correction for multiple testing was applied in the primary analysis, consistent with the exploratory and hypothesis-generating nature of untargeted metabolomics [33]. This decision is explicitly justified as follows: with 474 metabolites tested simultaneously at α = 0.05, approximately 24 false positives are expected by chance. Accordingly, all reported *p*-values must be interpreted as nominal, and the identified metabolites represent candidates for future validation rather than confirmed biomarkers. False-positive inflation was mitigated by applying a dual significance criterion (*p* < 0.05 AND FC > 1.5) and by corroborating findings with multivariate PLS-DA models and pathway enrichment analysis. To formally assess robustness, Benjamini–Hochberg false discovery rate (FDR) correction was applied as a post hoc sensitivity analysis: 133 of 186 metabolites (72%) in the SCM-D2 vs. H-D2 comparison, 293 of 316 (93%) in the SCM temporal comparison, and 311 of 316 (98%) in the H temporal comparison survived at q < 0.05, confirming that the primary findings are robust to multiple testing correction. In contrast, only 1 of 36 metabolites survived FDR correction in the SCM-D21 vs. H-D21 comparison, consistent with the weaker group separation at day 21. In all volcano plots and figure captions, metabolites surviving FDR correction (q < 0.05) are visually distinguished from those that are nominally significant only (*p* < 0.05, uncorrected).

Although the temporal comparisons (SCM-D2 vs. SCM-D21; H-D2 vs. H-D21) involve paired samples (same udder quarter sampled at both time points), unpaired *t*-tests were applied for consistency across all four comparisons. This approach is conservative, as it does not leverage within-cow variance reduction. The strong group separation observed in both temporal comparisons despite unpaired testing (Q^2^ > 0.90, accuracy = 100%, PERMANOVA *p* = 0.001) indicates that the temporal metabolic shifts are robust. As an additional sensitivity analysis, paired Welch’s *t*-tests were performed for the two temporal comparisons using within-quarter pairing; the number of significantly altered metabolites remained essentially unchanged, confirming that the unpaired analysis was conservative rather than anti-conservative (Supplementary Tables S6 and S7).

#### 2.6.5. Correlation Network Analysis

A metabolite–metabolite correlation network was constructed from the 80 most variable metabolites (by variance across all biological samples after auto-scaling). Pairwise Pearson correlation coefficients were computed, and edges were retained where |r| > 0.75. The network was visualised using a force-directed (spring) layout algorithm. Node size is proportional to degree (number of connections), and edge colour indicates the sign of the correlation (red = positive; blue = negative). Nodes were coloured by KEGG pathway assignment.

#### 2.6.6. Hierarchical Cluster Analysis

Hierarchical cluster analysis (HCA) was performed on the top 50 metabolites ranked by one-way ANOVA F-statistic across all four experimental groups. Euclidean distance was used as the dissimilarity metric and Ward’s minimum variance method as the agglomeration criterion. The resulting heatmap displays auto-scaled values (Z-scores), with group membership indicated by a colour bar.

#### 2.6.7. Pathway Enrichment Analysis

Quantitative enrichment analysis (QEA) was performed using the metabolite set enrichment analysis (MSEA) module in MetaboAnalyst 6.0. Unlike over-representation analysis, QEA uses the full concentration table and applies the globaltest algorithm [34] to assess whether functionally related metabolite sets show coordinated changes between groups. Enrichment was tested against the Small Molecule Pathway Database (SMPDB) metabolite set library. Pathways were considered significantly enriched at a Holm-adjusted *p*-value < 0.05. Detected metabolites were additionally assigned to metabolic pathways based on the Kyoto Encyclopedia of Genes and Genomes (KEGG) database [35] for pathway-level visualization. In addition, to visualize the pathway-level distribution of significantly altered metabolites across comparisons, metabolites reaching significance (*p* < 0.05, Student’s *t*-test) were assigned to KEGG pathway categories and the number of significant metabolites per pathway was tallied for each comparison.

#### 2.6.8. Software and Reproducibility

Multivariate analyses, ROC curves, univariate tests, hierarchical clustering, correlation network analysis, and all publication-quality figures were generated in Python 3.11 using NumPy v1.26, pandas v2.1, scikit-learn v1.4 [36] for PCA, PLS-DA (Table 1), cross-validation, and ROC analysis, SciPy v1.12 [37] for hierarchical clustering and statistical tests, and Matplotlib v3.8 [38] for figure generation. Metabolite set enrichment analysis was performed within MetaboAnalyst 6.0 [27]. Figures were exported at 600 dpi resolution in both PNG and SVG formats. The metabolomics data processing pipeline (CIL-LC-MS) was performed on the NovaMT platform (NovaMT Inc., Edmonton, AB, Canada).

**Table 1:** * PLS-DA model performance and PERMANOVA results for each pairwise comparison of bovine dry secretion metabolomes (474 metabolites; n = 10 per group).

Comparison	R^2^Y	Q^2^	Accuracy	AUC	*p* (R^2^)	*p* (Q^2^)	PERMANOVA (PCA)	PERMANOVA (PLS-DA)
SCM-D2 vs. H-D2	0.924	0.697	100%	1.00	<0.005	<0.005	F = 4.74, *p* = 0.001	F = 27.1, *p* = 0.001
SCM-D21 vs. H-D21	0.902	0.361	80%	0.87	0.015	0.010	F = 1.57, *p* = 0.084	F = 20.1, *p* = 0.001
SCM-D2 vs. SCM-D21	0.987	0.920	100%	1.00	<0.005	<0.005	F = 10.6, *p* = 0.001	F = 88.2, *p* = 0.001
H-D2 vs. H-D21	0.991	0.972	100%	1.00	<0.005	<0.005	F = 12.3, *p* = 0.001	F = 70.4, *p* = 0.001
4-group (overall)	0.734	0.527	80%	—	<0.005	—	F = 9.16, *p* = 0.001	F = 67.2, *p* = 0.001

* Model quality assessed by R^2^Y (goodness of fit) and Q^2^ (predictive ability, LOOCV). Classification accuracy: proportion of correctly classified samples during LOOCV. AUC: area under the ROC curve. Permutation testing: 200 permutations; empirical *p*-values computed as (r + 1)/(n_perm_ + 1). PERMANOVA: Euclidean distance, 999 permutations applied to both PCA and PLS-DA score matrices. Dash (—) indicates metric not applicable for the 4-group model.

## 3. Results

### 3.1. Overall Metabolome Overview

The HP-CIL LC–MS platform detected and quantified 474 metabolites across the four experimental groups (SCM-D2, SCM-D21, H-D2, and H-D21). PCA of the full dataset revealed clear group separation, with PC1 and PC2 accounting for 33.8% and 14.3% of the total variance, respectively (Figure 1A). Notably, the primary axis of variation (PC1) corresponded to the time dimension (D2 vs. D21), while health status (SCM vs. H) contributed mainly to PC2. Groups sampled at the same time point—particularly SCM-D21 and H-D21—showed partial overlap in PCA space, suggesting that the metabolic differences between health conditions diminish over the dry period. The scree plot indicated that five components were required to capture >65% of total variance, consistent with the high dimensionality of the metabolome (Figure 1C). Quality control samples clustered tightly near the origin, confirming analytical reproducibility (Figure 1D). PERMANOVA (Table 2) confirmed a statistically significant overall group structure (pseudo-F = 9.16, *p* = 0.001; Euclidean distance, 999 permutations).

**Table 2:** * Number of significantly altered metabolites per pairwise comparison of bovine dry secretion metabolomes (Welch’s *t*-test, *p* < 0.05, FC > 1.5 or < 1/1.5).

Comparison	Total Sig.	Direction (↑/↓)	Survive FDR	FDR Survival	VIP > 1	VIP > 2	% of 474
SCM-D2 vs. H-D2	186	120 ↑/66 ↓	133	71.5%	228	0	39.2%
SCM-D21 vs. H-D21	36	26 ↑/10 ↓	1	2.8%	177	4	7.6%
SCM-D2 vs. SCM-D21	316	207 ↑/109 ↓	293	92.7%	246	0	66.7%
H-D2 vs. H-D21	316	224 ↑/92 ↓	311	98.4%	246	0	66.7%

* Direction: number of metabolites with higher (↑) or lower (↓) abundance in the test group relative to the reference group. FDR: Benjamini–Hochberg corrected at q < 0.05. VIP: number of metabolites exceeding the indicated threshold from the corresponding PLS-DA model. % of 474: proportion of the total detected metabolome that was significantly altered. Abbreviations for Table 1 and Table 2: SCM, subclinical mastitis; H, healthy; D2, day 2 of dry period; D21, day 21 of dry period; LOOCV, leave-one-out cross-validation; AUC, area under the curve; FDR, false discovery rate; VIP, variable importance in projection; FC, fold change; PERMANOVA, permutational multivariate analysis of variance.

**Figure 2:**
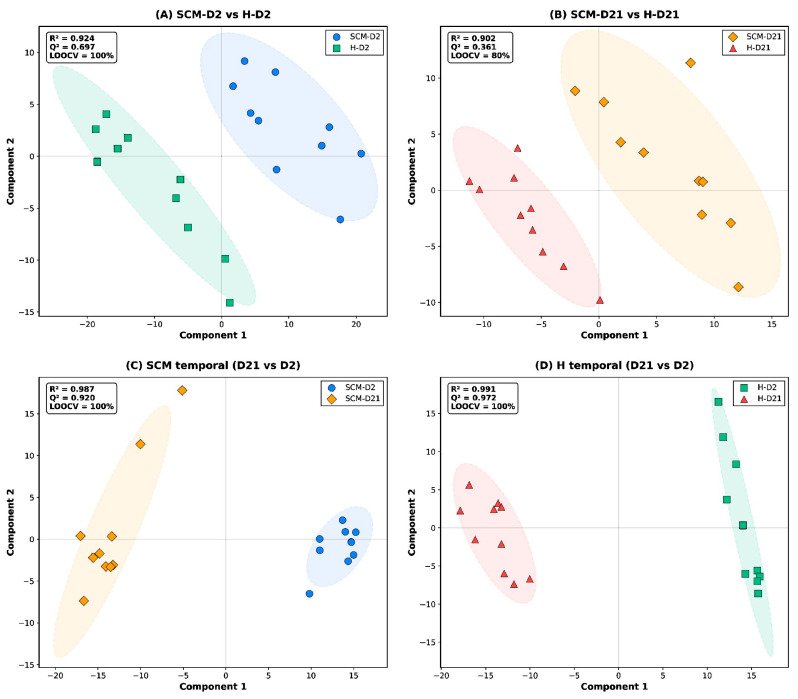
Partial least squares discriminant analysis (PLS-DA) score plots for four pairwise comparisons of bovine dry secretion metabolomes (n = 10 per group; 474 metabolites). Two-component models were fitted for each comparison; 95% confidence ellipses are shown. (**A**) SCM-D2 vs. H-D2: clear separation with strong model performance (R2 = 0.924; Q2 = 0.697; leave-one-out cross-validation [LOOCV] accuracy = 100%). (**B**) SCM-D21 vs. H-D21: reduced separation reflecting the attenuated disease signature by Day 21 (R2 = 0.902; Q2 = 0.361; LOOCV accuracy = 80%). The lower Q2 indicates reduced predictive ability, consistent with only 36 metabolites remaining significantly altered at this time point. (**C**) SCM temporal (D21 vs. D2): excellent discrimination (R2 = 0.987; Q2 = 0.920; LOOCV accuracy = 100%). (**D**) H temporal (D21 vs. D2): the strongest model overall (R2 = 0.991; Q2 = 0.972; LOOCV accuracy = 100%), confirming that involution drives the most reproducible metabolic separation. R2 = goodness of fit; Q2 = predictive ability estimated by LOOCV. Data were auto-scaled prior to modelling.

PLS-DA improved group separation (Figure 2). The three-component PLS-DA model achieved R^2^Y = 0.734 and Q2 = 0.527 (LOOCV), with a cross-validated classification accuracy of 80% across all four groups. Permutation testing (200 permutations) confirmed that the model was statistically significant (*p* < 0.005 for R2). PERMANOVA applied to the PLS-DA score matrix further confirmed significant group separation (pseudo-F = 67.2, *p* = 0.001). The top 20 metabolites contributing to multivariate discrimination among all four groups are listed in Supplementary Table S2 and included uracil (pyrimidine metabolism), N-acetylindoxyl (tryptophan metabolism), L-cysteinylglycine disulfide (glutathione metabolism), and pantothenic acid (CoA biosynthesis).

### 3.2. Health Status-Associated Metabolite Alterations

#### 3.2.1. Early Dry Period (SCM-D2 vs. H-D2)

In the early dry period, PCA explained 39.6% (PC1) and 9.2% (PC2) of the variance, with partial overlap between groups; PLS-DA achieved clearer separation (Figure 2A). The two-component PLS-DA model yielded R2Y = 0.924 and Q2 = 0.697 (LOOCV), with a cross-validated classification accuracy of 100% (AUC = 1.00). Permutation testing confirmed model significance (*p* < 0.005 for both R2 and Q2; Supplementary Figure S1A). PERMANOVA indicated significant group separation on both the PCA score matrix (pseudo-F = 4.74, *p* = 0.001) and the PLS-DA score matrix (pseudo-F = 27.1, *p* = 0.001).

**Figure 3:**
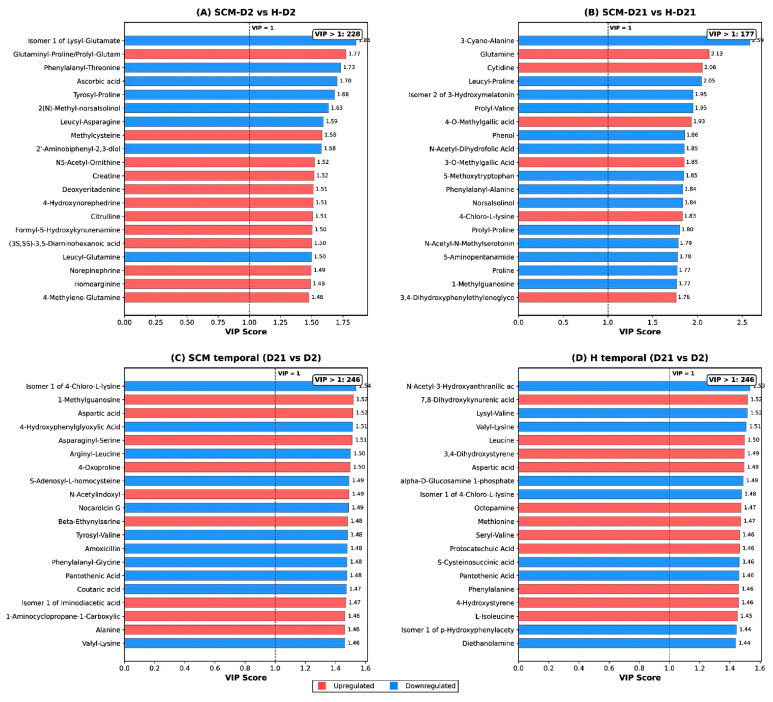
Variable importance in projection (VIP) scores from PLS-DA models: top 20 metabolites per pairwise comparison. VIP scores quantify the contribution of each metabolite to group separation; metabolites with VIP > 1 (dashed line) are considered important discriminators. Bar colour indicates direction of change: red = upregulated in the first-named group; blue = downregulated. (**A**) SCM-D2 vs. H-D2: 228 metabolites exceeded VIP > 1. Top-ranked metabolites include dipeptides (Lysyl-Glutamate, Glutaminyl-Proline/Prolyl-Glutamine) and catecholamine derivatives, consistent with neutrophil-driven proteolysis and adrenergic depletion. (**B**) SCM-D21 vs. H-D21: 177 metabolites exceeded VIP > 1 despite only 36 being individually significant, indicating that multi-metabolite patterns retain discriminatory power when individual effect sizes are small. 3-Cyano-Alanine was the top-ranked discriminator. (**C**) SCM temporal (D21 vs. D2): 246 metabolites exceeded VIP > 1; Isomer 1 of 4-Chloro-L-lysine, 1-Methylguanosine, and Aspartic acid led the ranking. (**D**) H temporal (D21 vs. D2): 246 metabolites exceeded VIP > 1; N-Acetyl-3-Hydroxyanthranilic acid and Lysyl-Valine were the top discriminators. Two-component PLS-DA models; n = 10 per group.

**Figure 4:**
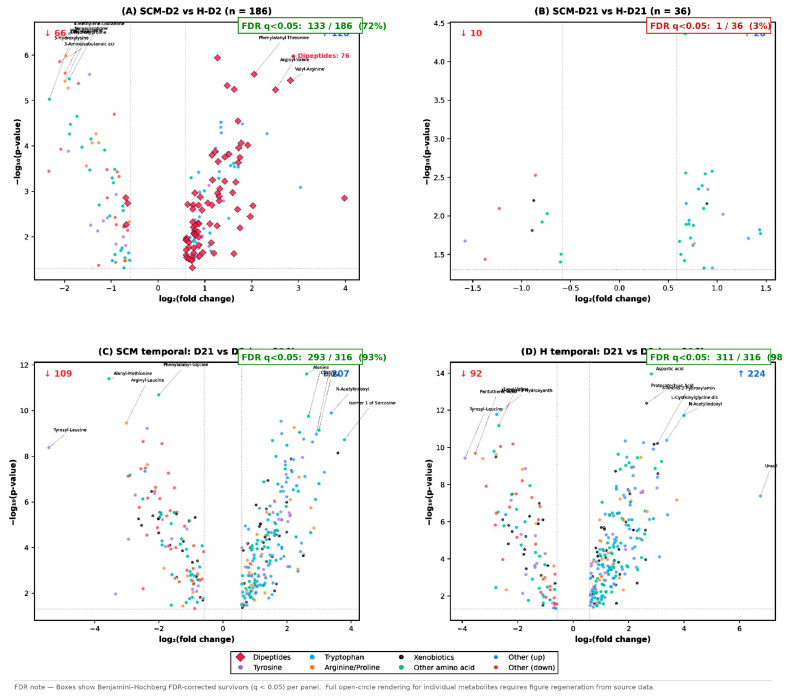
Volcano plots of significantly altered metabolites in bovine dry secretions across four pairwise comparisons. Each point represents one metabolite; the *x*-axis shows log2(fold change) and the *y*-axis shows –log10(*p*-value) from Welch’s *t*-test. Horizontal dashed line: *p* = 0.05; vertical dashed lines: |FC| = 1.5. Filled circles: metabolites surviving Benjamini–Hochberg FDR correction (q < 0.05); open circles: nominally significant only (*p* < 0.05, uncorrected). Statistical criteria: Welch’s *t*-test, *p* < 0.05 and |FC| > 1.5 (primary); FDR q < 0.05 (sensitivity, indicated by filled symbols). (**A**) SCM-D2 vs. H-D2 (n = 186 significant; 133 FDR-corrected): 120 upregulated and 66 downregulated. Dipeptides (rose diamonds) dominated the upregulated fraction (76 of 120; 63%), consistent with neutrophil-driven proteolysis. (**B**) SCM-D21 vs. H-D21 (n = 36 significant; 1 FDR-corrected): 26 upregulated and 10 downregulated. The near-absence of FDR survivors reflects the weaker group separation at Day 21. Note the compressed fold-change scale relative to panel A. (**C**) SCM temporal, D21 vs. D2 (n = 316 significant; 293 FDR-corrected): 207 accumulated and 109 depleted. (**D**) H temporal, D21 vs. D2 (n = 316 significant; 311 FDR-corrected): 224 accumulated and 92 depleted. Note the dramatic scale difference between health comparisons (**A**,**B**) and temporal comparisons (**C**,**D**), reflecting the dominance of involution-driven metabolic remodeling. Metabolites are colour-coded by KEGG pathway. Filled circles: FDR-corrected (q < 0.05); open circles: nominally significant only (*p* < 0.05, uncorrected). n = 10 per group; CIL-LC-MS platform.

Univariate analysis identified 186 significantly altered metabolites (120 upregulated, 66 downregulated) in SCM-D2 relative to H-D2 (*p* < 0.05, FC > 1.5; Figure 3A and Figure 4A; Supplementary Tables S3 and S4). The most striking feature of this comparison was the dominance of dipeptides among the upregulated metabolites: 76 of 120 upregulated metabolites (63%) were dipeptides classified under “protein digestion and absorption,” including valyl-arginine (FC = 7.11, *p* = 3.6 × 10^−6^), phenylalanyl-threonine (FC = 4.15, *p* = 2.7 × 10^−6^), arginyl-valine (FC = 5.69, *p* = 5.8 × 10^−6^), and tyrosyl-leucine (FC = 3.92, *p* = 3.6 × 10^−3^). This pronounced dipeptide surge suggests enhanced proteolytic activity in SCM-affected quarters at the onset of the dry period.

Among the downregulated metabolites, several free amino acids and related metabolites were notable, including norepinephrine (FC = 0.27, *p* = 3.4 × 10^−7^), citrulline (FC = 0.26, *p* = 1.0 × 10^−6^), 4-hydroxyproline (FC = 0.25, *p* = 3.8 × 10^−6^), creatine (FC = 0.40, *p* = 5.4 × 10^−5^), glycine (FC = 0.42, *p* = 2.1 × 10^−4^), and hypotaurine (FC = 0.24, *p* = 5.3 × 10^−6^). The most frequently affected pathways among significant metabolites were protein digestion and absorption (76 metabolites), tyrosine metabolism (18), tryptophan metabolism (12), and arginine and proline metabolism (9). The top 20 VIP-ranked metabolites for this comparison (Figure 3A; Supplementary Table S3) were led by isomer 1 of lysyl-glutamate, glutaminyl-proline/prolyl-glutamine, and phenylalanyl-threonine.

#### 3.2.2. Later Dry Period (SCM-D21 vs. H-D21)

By day 21, the metabolic distinction between SCM and H quarters was markedly attenuated. PCA showed substantial overlap between the two groups (PC1: 27.1%; PC2: 20.2%), and PLS-DA was required to achieve separation (Figure 2B). The two-component PLS-DA model yielded R2Y = 0.902 and Q2 = 0.361 (LOOCV), with a cross-validated classification accuracy of 80% (AUC = 0.87). While the model was statistically significant by permutation testing (*p* = 0.010 for both R2 and Q2; Supplementary Figure S1B), the lower Q2 and reduced accuracy compared with the D2 comparison indicate weaker discriminatory power. Notably, PERMANOVA applied to the PCA score matrix was not significant (pseudo-F = 1.57, *p* = 0.084), confirming the substantial group overlap in unsupervised space, although PERMANOVA on PLS-DA scores was significant (pseudo-F = 20.1, *p* = 0.001).

Only 36 significantly altered metabolites (26 upregulated, 10 downregulated) were identified (Figure 4B; Supplementary Table S5)—a five-fold reduction compared with the D2 comparison. The most significant metabolite was 3-cyano-alanine (Up, FC = 1.60, *p* = 4.3 × 10^−5^; cyanoamino acid metabolism). Several proline-containing dipeptides were upregulated, including prolyl-proline (FC = 2.72), leucyl-proline (FC = 1.93), alanyl-proline (FC = 1.80), and free proline itself (FC = 1.70, *p* = 0.023). The dominant pathway was amino acid metabolism (13 metabolites), followed by tryptophan metabolism (4) and tyrosine metabolism (3). Of the top 20 VIP-ranked metabolites for this comparison (Figure 3B; Supplementary Table S3), 6 did not meet the univariate significance thresholds (*p* < 0.05 and FC > 1.5), reflecting a degree of discordance between multivariate and univariate approaches when group differences are subtle.

#### 3.2.3. Cross-Comparison: Convergence of SCM and H Metabolomes over Time

**Figure 5:**
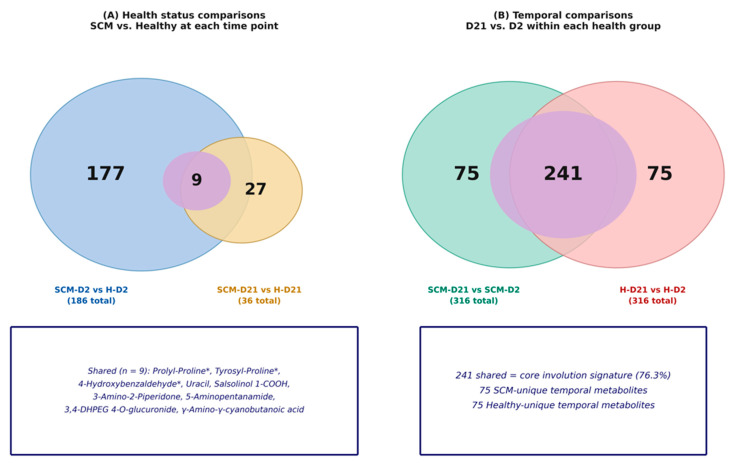
Venn diagrams of significantly altered metabolites (*p* < 0.05, |FC| > 1.5) in bovine dry secretions. (**A**) Overlap between health-status comparisons on Day 2 and Day 21 post-dry-off. Of 186 metabolites significantly altered at D2, only 9 (4.8%) remained significant at D21, demonstrating that 95.2% of SCM-associated metabolite alterations resolved over the dry period. The 9 shared metabolites (listed) include Prolyl-Proline and Tyrosyl-Proline (asterisks: significant in all four pairwise comparisons), 4-Hydroxybenzaldehyde, Uracil, Salsolinol 1-COOH, 3-Amino-2-Piperidone, 5-Aminopentanamide, 3,4-DHPEG 4-O-glucuronide, and γ-Amino-γ-cyanobutanoic acid. (**B**) Overlap between temporal comparisons (D21 vs. D2) within SCM and healthy groups. Of 316 significant metabolites in each comparison, 241 (76.3%) were shared, representing a core involution signature common to both health conditions. Each group exhibited 75 unique temporal metabolites, indicating that SCM modifies approximately 23.7% of the involution-associated metabolic trajectory. n = 10 per group; CIL-LC-MS platform; 474 total metabolites detected. * Significant in all four pairwise comparisons (*p* < 0.05, |FC| > 1.5).

A comparison of the two health-status analyses revealed a striking temporal pattern: the number of SCM-associated metabolite alterations decreased from 186 at D2 to 36 at D21, and only 9 metabolites were significantly altered in both comparisons (Figure 5A), including uracil, prolyl-proline, tyrosyl-proline, 4-hydroxybenzaldehyde/3-hydroxybenzaldehyde, and salsolinol 1-carboxylic acid. This convergence was paralleled by a decline in PLS-DA model strength: R2Y decreased modestly from 0.924 to 0.902, but Q2 fell sharply from 0.697 to 0.361, and classification accuracy dropped from 100% to 80%, indicating that the metabolic separation between SCM and H quarters became less robust as the dry period progressed.

**Figure 6:**
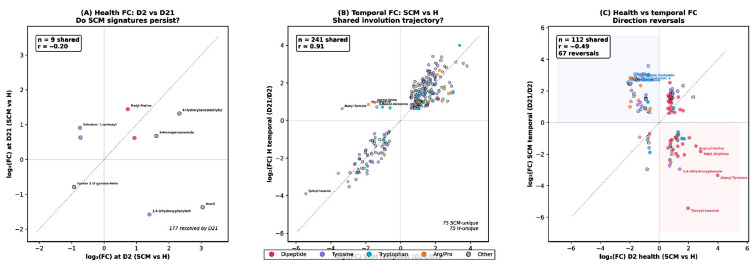
Fold-change correlation plots across comparisons. Metabolites are colour-coded by class: dipeptides (rose), tyrosine derivatives (purple), tryptophan metabolites (blue), arginine/proline metabolites (orange), and other (grey). (**A**) Health-status fold change at D2 vs. D21: Do SCM signatures persist? Each point represents a metabolite significant in both health-status comparisons (n = 9 shared). The weak negative correlation (r = −0.20) and the clustering of metabolites near the origin on the D21 axis indicate that fold-change magnitudes diminished substantially by Day 21. Annotation “177 resolved by D21” indicates metabolites that lost significance. (**B**) Temporal fold change in SCM vs. H: Shared involution trajectory? Each point represents a metabolite significant in both temporal comparisons (n = 241 shared). The tight diagonal correlation (r = 0.91) confirms that the direction and magnitude of involution-driven changes are highly conserved between health groups. 75 metabolites were unique to each trajectory. (**C**) D2 health-status FC vs. SCM temporal FC: Direction reversals. Each point represents a metabolite significant in both comparisons (n = 112 shared). The negative correlation (r = −0.49) indicates that metabolites depleted in SCM at D2 tended to accumulate during involution and vice versa. Shaded quadrants highlight 67 direction reversals: blue = metabolites depleted in SCM at D2 that accumulated during involution; red = metabolites elevated in SCM at D2 that were subsequently depleted. n = 10 per group. Note: 75 metabolites were unique to each temporal comparison and are not included in this plot.

Fold-change correlation analysis (Figure 6A) confirmed this convergence quantitatively: the 9 shared metabolites showed a weak negative correlation between their D2 and D21 fold changes (r = −0.20), with most metabolites clustering near the origin on the D21 axis, indicating loss of effect magnitude. The remaining 177 metabolites that were significant at D2 but not at D21 are annotated in the plot. This convergence suggests that the metabolic signature of subclinical mastitis in dry secretions is most pronounced during the early dry period and diminishes substantially by day 21, potentially reflecting the resolution of active inflammatory processes or the dominance of involution-related metabolic shifts that mask health-status differences.

### 3.3. Temporal Metabolite Alterations During the Dry Period

#### 3.3.1. Temporal Shift Within SCM Quarters (SCM-D2 vs. SCM-D21)

The temporal comparison within SCM cows revealed profound metabolic remodeling. PCA explained 55.9% (PC1) and 8.6% (PC2) of the total variance, and both PCA and PLS-DA showed complete separation between D2 and D21 (Figure 2C), indicating that time-related metabolic changes far exceeded health-status differences in magnitude. The PLS-DA model was exceptional: R2Y = 0.987, Q2 = 0.920 (LOOCV), accuracy = 100%, AUC = 1.00; permutation testing confirmed significance (*p* < 0.005 for both R2 and Q2; Supplementary Figure S1C). PERMANOVA was significant on both the PCA score matrix (pseudo-F = 10.6, *p* = 0.001) and PLS-DA scores (pseudo-F = 88.2, *p* = 0.001).

A total of 316 significantly altered metabolites (207 upregulated, 109 downregulated) were identified in SCM-D21 relative to SCM-D2 (Figure 4C; Supplementary Table S6)—representing 65.6% of the entire detected metabolome. The most significant changes included aspartic acid (Up, FC = 6.13, *p* = 2.5 × 10^−12^), N-acetylindoxyl (Up, FC = 10.45, *p* = 1.3 × 10^−10^), glycine (Up, FC = 8.0, *p* = 7.3 × 10^−10^), and alanine (Up, FC = 6.37, *p* = 1.8 × 10^−10^). Downregulated metabolites included several dipeptides such as alanyl-methionine (FC = 0.085, *p* = 4.1 × 10^−12^) and tyrosyl-leucine (FC = 0.023, *p* = 4.1 × 10^−9^). The affected pathways spanned xenobiotics metabolism (53 metabolites), arginine and proline metabolism (33), tyrosine metabolism (32), lysine metabolism (30), alanine/aspartate/glutamate metabolism (26), and tryptophan metabolism (26). The top 20 VIP-ranked metabolites (Figure 3C; Supplementary Table S3) were led by isomer 1 of 4-chloro-L-lysine, 1-methylguanosine, and aspartic acid.

#### 3.3.2. Temporal Shift Within Healthy Quarters (H-D2 vs. H-D21)

The temporal metabolic shift in healthy cows was equally extensive. PCA explained 54.2% (PC1) and 12.0% (PC2) of the total variance with complete group separation in both PCA and PLS-DA (Figure 2D). The PLS-DA model achieved the highest performance of all comparisons: R2Y = 0.991, Q2 = 0.972 (LOOCV), accuracy = 100%, AUC = 1.00; permutation testing confirmed significance (*p* < 0.005; Supplementary Figure S1D). PERMANOVA was highly significant on both PCA scores (pseudo-F = 12.3, *p* = 0.001) and PLS-DA scores (pseudo-F = 70.4, *p* = 0.001).

Univariate analysis identified 316 significantly altered metabolites (224 upregulated, 92 downregulated) in H-D21 relative to H-D2 (Figure 4D; Supplementary Table S7), a number identical to the SCM temporal comparison. The most highly significant metabolites were aspartic acid (Up, FC = 7.07, *p* = 1.1 × 10^−14^), protocatechuic acid (Up, FC = 6.28, *p* = 4.3 × 10^−13^), N-acetylindoxyl (Up, FC = 16.00, *p* = 2.0 × 10^−12^), and L-cysteinylglycine disulfide (Up, FC = 10.38, *p* = 4.2 × 10^−11^). Pathway distributions closely mirrored those of the SCM temporal shift: xenobiotics metabolism (53), alanine/aspartate/glutamate metabolism (34), lysine metabolism (31), arginine and proline metabolism (29), and BCAA metabolism (28). The top 20 VIP-ranked metabolites (Figure 3D; Supplementary Table S3) were led by N-acetyl-3-hydroxyanthranilic acid, 7,8-dihydroxykynurenic acid, and lysyl-valine.

#### 3.3.3. Cross-Comparison: Shared vs. Unique Temporal Changes

Of the 316 significant metabolites in each temporal comparison, 241 (76.3%) were shared between SCM and H cows (Figure 5B), indicating that the dominant D2→D21 metabolic shift reflects physiological mammary involution processes common to both healthy and SCM-affected quarters. However, each group also exhibited 75 unique temporal metabolites not significantly altered in the other, suggesting that subclinical mastitis modifies the trajectory of involution-related metabolic remodeling in a subset of pathways.

The temporal fold-change correlation between the two health groups (Figure 6B) was strongly positive (r = 0.91, n = 241 shared metabolites), confirming that the direction and magnitude of involution-driven changes were highly conserved. In contrast, the correlation between D2 health-status fold changes and SCM temporal fold changes (Figure 6C) was negative (r = −0.49, n = 112 shared metabolites), with 67 direction reversals identified—metabolites depleted in SCM at D2 that accumulated during involution, or vice versa. This reversal pattern is consistent with a compensatory dynamic in which early SCM-associated depletions are corrected during involution-driven remodeling.

**Figure 7:**
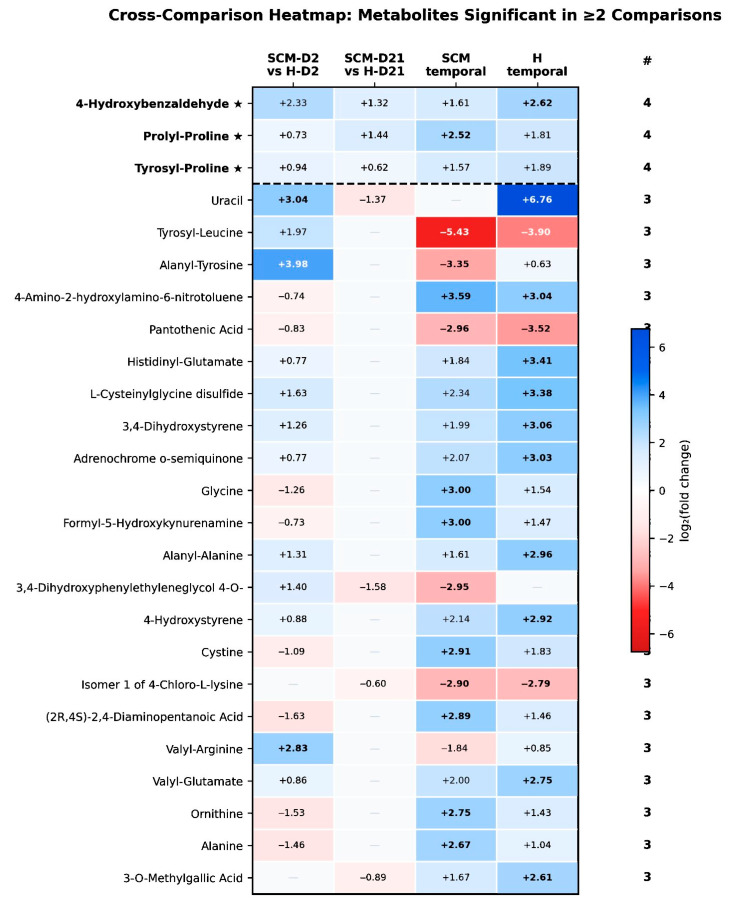
Cross-comparison heatmap of log2(fold change) for metabolites significantly altered in two or more pairwise comparisons. Columns represent the four pairwise comparisons; rows represent individual metabolites. Blue indicates upregulation; red indicates downregulation; grey dashes (—) denote non-significant results. Bold values indicate |log_2_FC| ≥ 2.5. The starred (★) metabolites—4-Hydroxybenzaldehyde, Prolyl-Proline, and Tyrosyl-Proline—were significant in all four comparisons, representing the most robust cross-condition markers. A dashed line separates four-comparison metabolites (top) from those significant in exactly three comparisons (bottom). The rightmost column (#) indicates the number of comparisons reaching significance (*p* < 0.05, uncorrected). Notable direction reversals are apparent for Glycine, Ornithine, Alanine, and Cystine, which were depleted in SCM at D2 but accumulated during involution in both health groups. n = 10 per group; 474 total metabolites.

A cross-comparison heatmap (Figure 7) of metabolites significant in two or more pairwise comparisons identified three metabolites—4-hydroxybenzaldehyde, prolyl-proline, and tyrosyl-proline—that were significantly altered in all four comparisons, representing the most robust cross-condition markers. Notable direction reversals were apparent for glycine, ornithine, alanine, and cystine, which were depleted in SCM at D2 but accumulated during involution in both health groups. The near-identical PLS-DA performance (SCM temporal: R2Y = 0.987, Q2 = 0.920; H temporal: R2Y = 0.991, Q2 = 0.976) further confirms that temporal metabolic remodeling is the dominant source of variation in both health conditions, consistent with the PCA observation that PC1 captured the time axis.

### 3.4. Quantitative Enrichment Analysis

Metabolite set enrichment analysis (MSEA) was performed to identify biologically relevant pathways enriched in each comparison. In the SCM-D2 vs. H-D2 comparison, all top 25 enriched pathways were statistically significant after Holm correction (*p* < 0.05), with *p*-values reaching 1 × 10^−7^ for the most enriched pathways. The five most represented pathways by metabolite count were protein digestion and absorption (78 metabolites), tyrosine metabolism (22), xenobiotics metabolism (21), arginine and proline metabolism (17), and tryptophan metabolism (17). Protein digestion and absorption was driven almost entirely by dipeptides, consistent with neutrophil-derived proteolytic activity in SCM-affected quarters. In contrast, the SCM-D21 vs. H-D21 comparison yielded only two significantly enriched pathways after Holm correction—phenylacetate metabolism and sulfate/sulfite metabolism—and far fewer significant metabolites per pathway (protein digestion and absorption: 14; xenobiotics metabolism: 10; tryptophan metabolism: 9), consistent with the reduced number of individually significant metabolites at Day 21.

Both temporal comparisons showed highly significant enrichment across all top 25 pathways, with Holm-adjusted *p*-values ranging from 7 × 10^−8^ to 6 × 10^−12^ for the SCM temporal comparison and 5 × 10^−8^ to 4 × 10^−13^ for the H temporal comparison (Supplementary Figure S3). In the SCM temporal comparison, the top five pathways were protein digestion and absorption (102 metabolites), xenobiotics metabolism (51), arginine and proline metabolism (28), tyrosine metabolism (26), and tryptophan metabolism (23). The H temporal comparison followed a similar hierarchy: protein digestion and absorption (114), xenobiotics metabolism (52), tyrosine metabolism (23), tryptophan metabolism (22), and lysine metabolism (22).

**Figure 8:**
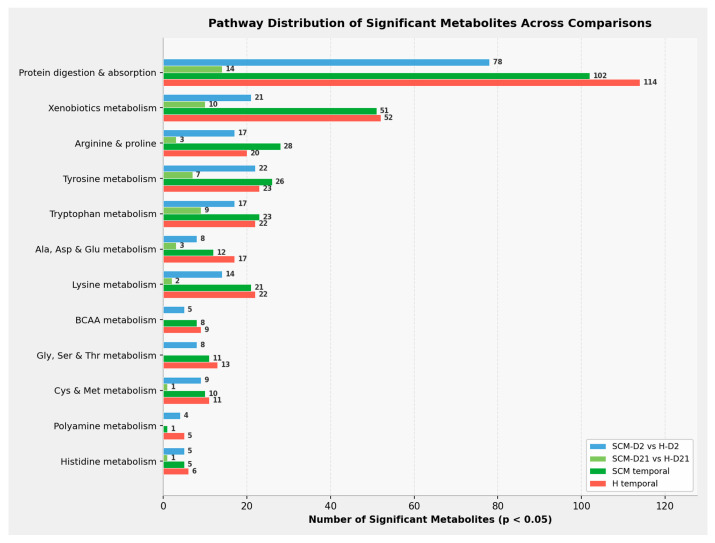
Grouped horizontal bar chart showing the pathway-level distribution of significantly altered metabolites (*p* < 0.05, Student’s *t*-test) across four pairwise comparisons. Bars represent the number of significant metabolites assigned to each of 12 KEGG-based pathway categories. Protein digestion and absorption (dipeptides) and xenobiotics metabolism were the most represented pathways. Temporal comparisons (D21 vs. D2) yielded higher metabolite counts across nearly all pathways than health-status comparisons, while SCM-D21 vs. H-D21 showed the fewest significant metabolites, confirming attenuation of the disease signature by Day 21. n = 10 per group; 474 metabolites; CIL-LC-MS platform.

The pathway-level distribution of significantly altered metabolites across all four comparisons (Figure 8) confirmed this asymmetry between health-status and temporal analyses. Temporal comparisons yielded the highest metabolite counts across nearly all pathways, while xenobiotics metabolism showed near-equivalent enrichment in both temporal comparisons (51–52 metabolites), reflecting accumulation of microbial-origin metabolites during involution irrespective of health status. The SCM-D21 vs H-D21 comparison yielded the fewest significant metabolites across all pathways, confirming the attenuation of the disease signature by Day 21.

### 3.5. Multivariate Validation and Integrative Analyses

To validate the PLS-DA models and provide additional integrative perspective, a suite of complementary analyses was performed. PLS-DA models were validated by permutation testing (200 permutations per model; Supplementary Figure S1) and receiver operating characteristic analysis (Supplementary Figure S2). All four models were statistically significant: permutation *p*-values were <0.005 for R^2^ and Q^2^ in three comparisons (SCM-D2 vs. H-D2, SCM temporal, H temporal), and 0.010 for the SCM-D21 vs. H-D21 comparison. ROC-AUC values were 1.00 for three comparisons and 0.87 for the attenuated D21 health comparison. Reduced 20-VIP metabolite panels retained high discriminatory power (AUC ≥ 0.96), suggesting that compact biomarker panels capture the essential discriminatory information.

**Figure 9:**
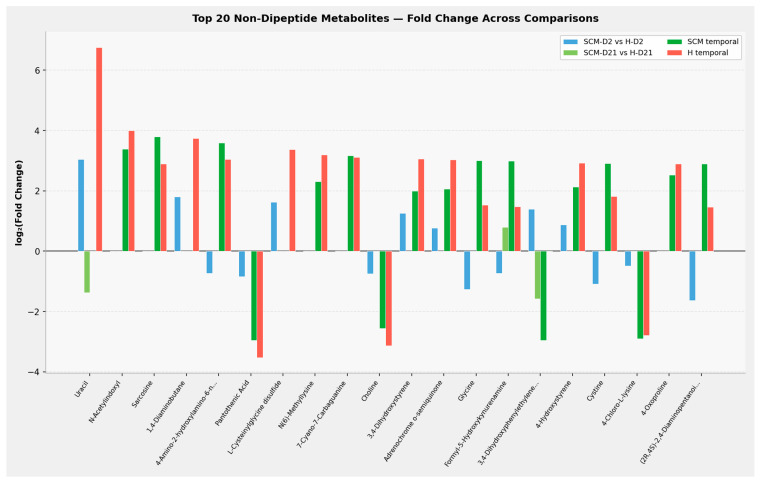
Grouped bar chart of log_2_(fold change) for the top 20 non-dipeptide metabolites ranked by maximum absolute fold change across four pairwise comparisons. Only metabolites significant (*p* < 0.05, Student’s *t*-test) in at least two comparisons are shown; bars are omitted where significance was not reached. Positive values indicate upregulation in the first-named group. Uracil exhibited the largest fold change (log_2_FC = 6.76, H temporal). Pantothenic acid and choline were consistently downregulated across comparisons (Supplementary Table S4), while several tyrosine derivatives showed concordant temporal accumulation in both health groups. n = 10 per group; CIL-LC-MS platform. Full metabolite names for abbreviated x-axis labels are provided in Supplementary Table S4.

Individual metabolite fold changes. To characterise the magnitude and direction of change for individual metabolites beyond the dipeptide class, the top 20 non-dipeptide metabolites were ranked by maximum absolute log_2_(fold change) across all four comparisons (Figure 9). Only metabolites significant (*p* < 0.05) in at least two comparisons were included. Uracil exhibited the largest fold change (log_2_FC = 6.76, H temporal), with marked accumulation during involution in healthy cows. Pantothenic acid and choline were consistently downregulated across comparisons, suggesting depletion of cofactor and one-carbon metabolism intermediates during the dry period. Several tyrosine derivatives (3,4-dihydroxystyrene, adrenochrome o-semiquinone, 4-hydroxystyrene) showed concordant temporal accumulation in both health groups, reflecting involution-driven catecholamine metabolism. Glycine and cystine exhibited direction reversals—depleted in SCM at D2 but accumulated during involution—consistent with the recovery pattern identified in the fold-change correlation analysis (Figure 6).

**Figure 10:**
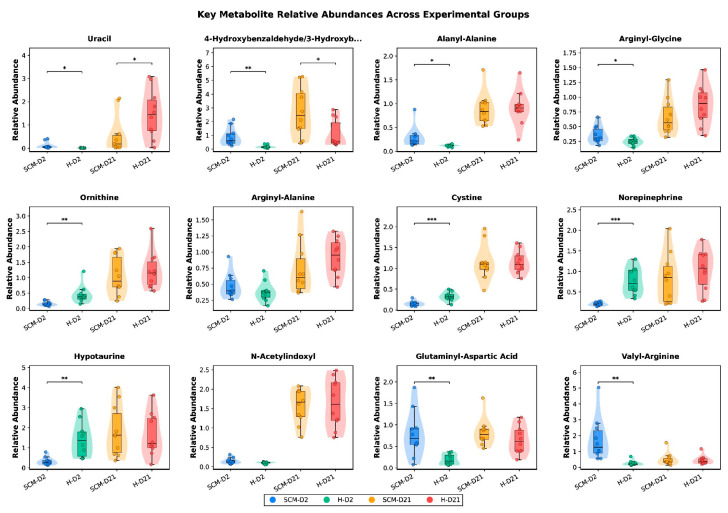
Box-violin plots of relative abundances for 12 representative metabolites spanning the major affected pathways. Each panel shows the distribution across four experimental groups (SCM-D2, blue; H-D2, green; SCM-D21, orange; H-D21, red). Violin shapes depict kernel density estimates; boxes show median and interquartile range; individual data points are overlaid. Significance brackets: * *p* < 0.05; ** *p* < 0.01; *** *p* < 0.001 (Welch’s *t*-test). Selected metabolites illustrate key biological themes: Uracil (pyrimidine metabolism) shows a dramatic increase in H-D21 (FC = 108.36); 4-Hydroxybenzaldehyde/3-Hydroxybenzaldehyde was significant in all four comparisons; Alanyl-Alanine and Valyl-Arginine (dipeptides) were elevated in SCM-D2, reflecting proteolytic activity; Arginyl-Glycine and Arginyl-Alanine (dipeptides) were elevated in SCM-D2, consistent with the proteolytic signature;Ornithine and Cystine exemplify direction reversals (depleted in SCM at D2, accumulating during involution); Norepinephrine and Hypotaurine were strongly depleted in SCM-D2, suggesting catecholamine depletion and oxidative stress; N-Acetylindoxyl (tryptophan/xenobiotics) accumulated prominently during involution in both groups. n = 10 per group.

Individual metabolite exemplars. Box-violin plots for 12 representative metabolites spanning the major affected pathways illustrate the distributions underlying the group-level statistics (Figure 10). Uracil showed the most dramatic temporal increase in healthy cows (FC = 108.36), while 4-hydroxybenzaldehyde was one of only three metabolites significant in all four comparisons. Dipeptides such as alanyl-alanine and valyl-arginine were elevated in SCM-D2, while norepinephrine and hypotaurine were strongly depleted, illustrating the proteolytic and catecholamine-depletion signatures, respectively. Ornithine and cystine exemplified direction reversals, being depleted in SCM at D2 but accumulating during involution in both groups.

**Figure 11:**
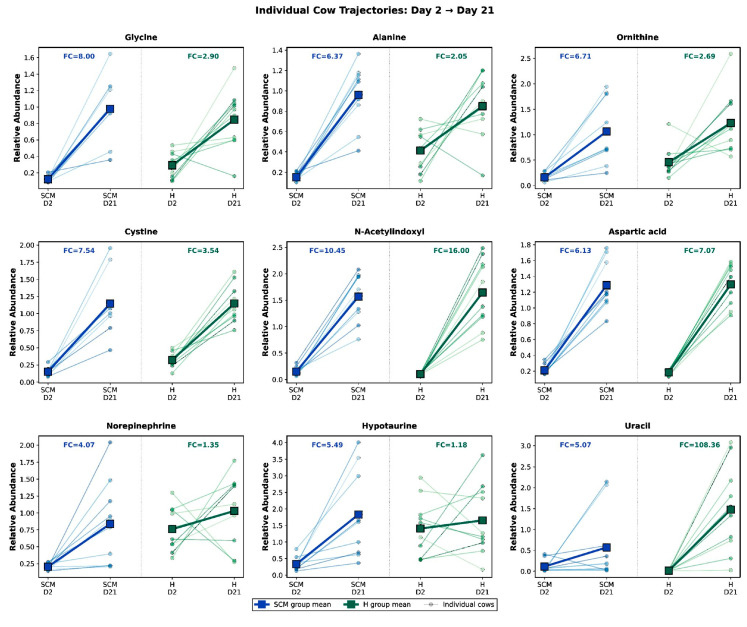
Individual cow trajectories from Day 2 to Day 21 for nine representative metabolites. Each thin line represents one cow (light blue = SCM; light green = H); bold lines with square markers show group means for SCM (blue) and H (green). Dashed vertical line separates SCM (left) from H (right) trajectories within each panel. Fold-change values are annotated for each group (blue = SCM; teal = H). For most metabolites, the direction of temporal change was highly consistent across individuals within each health group. Glycine, Alanine, and Ornithine showed steeper accumulation trajectories in SCM cows (FC = 8.00, 6.37, 6.71) compared with healthy cows (FC = 2.90, 2.05, 2.69), consistent with the direction-reversal pattern. N-Acetylindoxyl showed comparable fold changes in both groups (SCM FC = 10.45; H FC = 16.00), reflecting the shared involution programme. Norepinephrine and Hypotaurine accumulated more steeply in SCM (FC = 4.07, 5.49) than H (FC = 1.35, 1.18), indicating recovery from catecholamine depletion. Uracil exhibited the most divergent trajectories: SCM FC = 5.07 vs. H FC = 108.36, driven by very low D2 baseline levels in healthy cows. n = 10 per group.

Individual cow trajectories. Paired D2→D21 trajectory plots for individual cows (Figure 11) revealed that while the direction of temporal change was highly consistent across individuals for most metabolites (e.g., glycine, alanine, and aspartic acid universally increased from D2 to D21 in both groups), the magnitude of change differed between health conditions. Several metabolites showed steeper accumulation trajectories in SCM cows compared with healthy cows—glycine (SCM FC = 8.00 vs. H FC = 2.90), ornithine (6.71 vs. 2.69), and hypotaurine (5.49 vs. 1.18)—consistent with the compensatory reversal of early SCM-associated depletions. Uracil exhibited the most divergent trajectories (SCM FC = 5.07 vs. H FC = 108.36), driven by extremely low baseline levels in healthy cows at D2.

**Figure 12:**
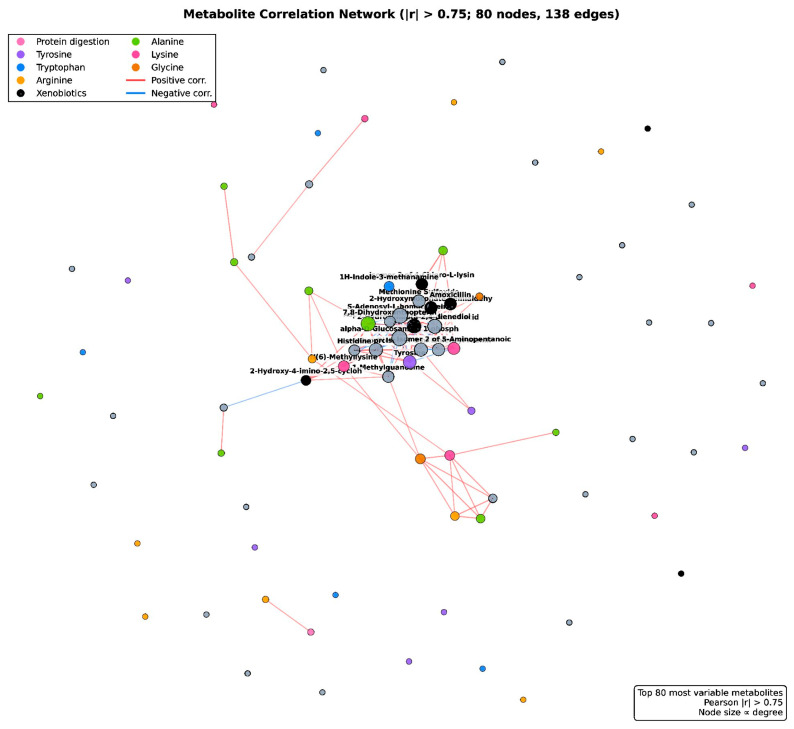
Metabolite–metabolite correlation network constructed from the top 80 most variable metabolites across all samples. Edges represent Pearson correlations with |r| > 0.75 (80 nodes, 138 edges). Red edges: positive correlations; blue edges: negative correlations. Node size is proportional to degree (number of connections). Nodes are colour-coded by KEGG pathway: protein digestion (light pink/rose), tyrosine (purple), tryptophan (blue), arginine (orange), xenobiotics (grey), alanine (green), lysine (magenta), and glycine (dark orange/brown). Two major co-regulation modules are evident: (i) a densely connected central cluster of free amino acids and xenobiotic metabolites that increased coordinately from D2 to D21, including 1H-Indole-3-methanamine, S-Adenosyl-L-homocysteine, and alpha-D-Glucosamine 1-phosphate; and (ii) a peripheral cluster of dipeptides and catecholamine-related metabolites that decreased coordinately. Cross-module negative correlations connect these two clusters, consistent with a shift from dipeptide accumulation (proteolysis) toward free amino acid release during mammary involution. n = 40 samples (10 per group); Pearson correlation on log2-transformed relative abundances.

Correlation network. A metabolite–metabolite correlation network (|r| > 0.75; Figure 12) comprising 80 nodes and 138 edges revealed two major co-regulation modules: (i) a densely connected central cluster of free amino acids and xenobiotic metabolites that increased coordinately from D2 to D21, and (ii) a peripheral cluster of dipeptides and catecholamine-related metabolites that decreased coordinately over time. Cross-module negative correlations connected these two clusters, consistent with a shift from dipeptide accumulation (proteolysis) toward free amino acid release during mammary involution.

## 4. Discussion

This study presents the first untargeted metabolomic characterization of bovine mammary dry secretion fluid. It reveals that subclinical mastitis (SCM) profoundly alters the amine/phenol submetabolome in the early dry period, but that these perturbations are largely resolved by day 21. Of 474 detected metabolites, 186 were significantly altered between SCM and healthy (H) quarters at day 2. This decreased to just 36 by day 21—an 80.6% attenuation that establishes the temporal arc of metabolic recovery. The significant metabolites spanned multiple biochemical classes—dipeptides, free amino acids, tryptophan catabolites, catecholamines, glutathione-cycle intermediates, and vitamins/cofactors—indicating that SCM perturbs interconnected metabolic networks rather than isolated pathways. While several studies have applied metabolomics to lactating milk from mastitic cows [12,14,39,40], no prior study has examined the metabolome of dry secretion fluid. This matrix reflects both mammary involution and immune activity without the dilution effect of active milk synthesis. In the discussion below, findings are distinguished between robust observations (those surviving FDR correction and corroborated by the existing literature) and exploratory observations (nominally significant candidates requiring validation in larger cohorts). We discuss the major biological themes emerging from these data in the context of existing mastitis and mammary involution literature.

### 4.1. Proteolytic Activation: The Dipeptide–Amino Acid Mirror

The most noticeable feature of the SCM-D2 metabolome was the large upregulation of dipeptides, which accounted for 76 of 120 upregulated metabolites (63%). This dipeptide signature—with fold changes reaching 4.2 (phenylalanyl-threonine), 3.3 (seryl-lysine), and 3.1 (leucyl-glutamine)—is consistent with elevated proteolytic activity in SCM quarters. Neutrophil-derived serine proteases (elastase, cathepsin G) and matrix metalloproteinases have been extensively documented in bovine mastitis milk. Prin-Mathieu et al. [41] demonstrated that polymorphonuclear neutrophils (PMNs) recruited during LPS-induced mastitis release proteases with caseinolytic activity at both neutral and acidic pH. Hinz et al. [42] showed that mastitic milk contains at least six distinct caseolytic activities, four of which differ from plasmin, with peak proteolytic activity coinciding with maximal tissue damage at 6–12 h post-challenge. Le Roux et al. [43] confirmed that PMN proteolysis is the primary driver of peptide accumulation in mastitic milk. They described two distinct waves: an early plasmin-driven phase and a later PMN protease-driven phase. This finding is robust: dipeptide accumulation at D2 survives FDR correction (q < 0.05) and is the single most numerically dominant signal in the entire dataset.

Our dipeptide findings align closely with and substantially extend the peptidomic observations of Thomas et al. [44], who reported increases in di-, tri-, and tetra-peptides during experimental *Streptococcus uberis* mastitis using untargeted LC-MS metabolomics. Similarly, Xi et al. [14] found that oligopeptides (Leu-Ala, Phe-Pro-Ile, Asn-Arg-Ala-Ile, and Val-Phe-Val-Tyr) increased by over 7.95-fold in clinical mastitis milk compared to healthy controls. However, neither study quantified the extent to which peptides dominated the altered metabolome. Our finding that dipeptides constituted 62% of all upregulated metabolites in dry secretion fluid establishes proteolysis as the single most prominent metabolic consequence of SCM, at least within the amine/phenol submetabolome captured by CIL-LC-MS.

Critically, the dipeptide elevation occurred against a backdrop of widespread free amino acid depletion. Citrulline (FC = 0.26), homoarginine (FC = 0.26), 4-hydroxyproline (FC = 0.25), creatine (FC = 0.40), alanine (FC = 0.36), glycine (FC = 0.42), sarcosine (FC = 0.37), and glutamine (FC = 0.45) were all significantly reduced at D2 in SCM quarters. This simultaneous dipeptide accumulation and amino acid depletion—a “dipeptide–amino acid mirror”—has not been previously reported in dairy mastitis studies and provides a coherent metabolic narrative: protein degradation releases dipeptide fragments (detected as elevated dipeptides), while the liberated amino acids are rapidly consumed by activated immune cells for energy production, nitric oxide synthesis, and antimicrobial peptide generation.

The depletion of citrulline and homoarginine is particularly consistent with this interpretation, as both are substrates for the arginine–nitric oxide synthase (NOS) pathway, which is activated during the bovine mammary innate immune response [45,46]. Citrulline is recycled to arginine via argininosuccinate synthetase, and its depletion suggests that the arginine–citrulline cycle is operating at high flux to sustain NO production. Interestingly, [14] reported that arginine was elevated in both clinical and subclinical mastitis milk during lactation, suggesting that the arginine–NOS pathway may operate differently in dry secretion fluid, where the absence of active milk synthesis removes a major competing sink for amino acid utilization.

The depletion of 4-hydroxyproline (FC = 0.25) is noteworthy. Hydroxyproline is a major component of collagen and a specific marker of collagen turnover. Its depletion in SCM quarters, combined with the elevation of epsilon-(gamma-glutamyl)-lysine (FC = 2.41, a transglutaminase crosslink product), suggests active extracellular matrix remodeling in the inflamed mammary tissue, consistent with the tissue damage observed during bovine mastitis [43,47].

The temporal resolution of this pattern was almost complete. By D21, citrulline recovered from FC = 0.26 to 0.94 (not significant), alanine from 0.36 to 1.13, glycine from 0.42 to 1.15, and norepinephrine from 0.27 to 0.81. Overall, 177 of 186 (95.2%) D2-significant metabolites were no longer significant at D21. The recovery was accompanied by large temporal increases in free amino acids within SCM cows (D2→D21: citrulline FC = 7.15, glycine FC = 8.00, alanine FC = 6.37), which substantially exceeded the corresponding increases in healthy cows (citrulline FC = 1.94, glycine FC = 2.90, alanine FC = 2.05). This asymmetry indicates that SCM quarters undergo a dual process: normal D2→D21 involution-related amino acid accumulation (which also occurs in healthy cows) plus recovery from inflammation-induced depletion, producing amplified temporal fold changes. Shamay et al. [48] demonstrated that plasmin, plasminogen, and plasminogen activator activities are all significantly elevated during the first 21 days of bovine mammary involution, providing the enzymatic basis for the progressive protein degradation and amino acid release that we observe metabolomically.

### 4.2. Tryptophan–Kynurenine Axis: Immune Activation and Indole Metabolite Divergence

Eleven tryptophan pathway metabolites were significantly altered in SCM-D2 quarters, revealing a bidirectional perturbation that implicates immune-mediated tryptophan catabolism. The kynurenine branch was depleted: N-formylkynurenine (FC = 0.27), kynurenine (FC = 0.59), 5-hydroxy-N-formylkynurenine (FC = 0.51), and formyl-5-hydroxykynurenamine (FC = 0.60). Simultaneously, the indole branch was elevated: 5-hydroxykynurenamine (FC = 3.28), 2,3-dihydroxyindole (FC = 2.93), 5,6-dihydroxyindole (FC = 2.46), and 1H-indole-3-methanamine (FC = 1.99). This pattern is consistent with the activation of indoleamine 2,3-dioxygenase (IDO), the rate-limiting enzyme in the kynurenine pathway, which is induced by pro-inflammatory cytokines (IFN-γ, TNF-α) during the innate immune response [49,50].

Bochniarz and colleagues have provided the most direct evidence for kynurenine pathway activation in bovine mastitis through a series of targeted studies. In subclinical mastitis caused by coagulase-negative staphylococci, tryptophan, kynurenine, and kynurenic acid concentrations were significantly lower in milk of affected cows compared to controls [51]. In *Streptococcus* spp. mastitis, IDO activity (calculated as the KYN/TRP ratio) was markedly elevated in milk, while tryptophan was depleted in both serum and milk [52]. In *Prototheca* mastitis, the same pattern was observed: tryptophan and kynurenine were significantly lower in mastitic milk, while IDO activity was significantly higher [53]. The authors proposed that upon infection, leukocytes and macrophages at the site of inflammation release IDO-inducing pro-inflammatory cytokines, diverting tryptophan toward the kynurenine pathway [53].

Our dry secretion fluid data are consistent with—but also extend—these targeted milk findings in several important ways. First, we detect the same directionality: kynurenine-branch metabolites are depleted, confirming high-flux catabolism. This finding is robust and survives FDR correction. Second, by capturing a broader metabolite panel through untargeted CIL-LC-MS, we reveal a simultaneous elevation of the indole branch (dihydroxyindoles, hydroxykynurenamine) that has not been reported in bovine mastitis before. The bidirectional split—kynurenine branch down, indole branch up—suggests preferential shunting through alternative tryptophan degradation routes. This may reflect accumulation of indoleamine pathway intermediates due to downstream bottlenecks; however, this mechanistic inference is exploratory and requires targeted validation. Third, we show that this perturbation is entirely resolved by D21, with all 11 metabolites returning to non-significant levels. The transient nature aligns with the known kinetics of IDO expression, which peaks during acute inflammation and returns to baseline as the immune response resolves [49]. These findings extend the tryptophan–kynurenine paradigm, well-characterized in human inflammatory disease [50] and recently established in bovine mastitis milk [51,52,54], to bovine dry-period mammary pathophysiology for the first time.

### 4.3. Catecholamine Depletion and Neuroimmune Signaling

Norepinephrine was among the most depleted metabolites at D2 (FC = 0.27, *p* = 4.0 × 10^−5^), accompanied by the depletion of 4-aminocatechol (FC = 0.27) and 3-methoxy-4-hydroxyphenylglycolaldehyde (FC = 0.54), a norepinephrine degradation product. Conversely, 3,4-dihydroxyphenylacetaldehyde (DOPAL; FC = 2.13), the aldehyde intermediate of catecholamine catabolism by monoamine oxidase, was elevated, as was di-hydroxymelatonin (FC = 2.54). This constellation—catecholamine depletion combined with catabolic intermediate elevation—indicates accelerated catecholamine turnover in SCM quarters.

Catecholamine–immune interactions in the mammary gland have received limited attention. Norepinephrine is a functional immunomodulator: β-adrenergic signaling regulates neutrophil chemotaxis, macrophage cytokine production, and epithelial tight junction integrity [54]. Madden et al. [55] demonstrated in rodent models that chemical sympathectomy depresses T-cell immunity, establishing a direct link between catecholamine signaling and immune function. In the bovine mammary gland, sympathetic innervation regulates blood flow and epithelial permeability. Both of these processes are disrupted during mastitis [45]. The substantial depletion of norepinephrine in SCM quarters may reflect consumption by immune cells, reduced local sympathetic nerve release due to tissue damage, or both. The concurrent elevation of DOPAL suggests that monoamine oxidase activity is increased, consistent with a high-turnover state. To our knowledge, this is the first report of catecholamine perturbation in bovine mammary secretion fluid. It suggests a neuroimmune dimension to the SCM response that warrants further investigation. This finding should be considered exploratory: norepinephrine depletion was the most significantly depleted individual metabolite and survived FDR correction (q < 0.05), but the mechanistic interpretation requires independent validation with targeted assays.

Like the amino acid and tryptophan perturbations, catecholamine depletion was temporally resolved. Norepinephrine recovered from FC = 0.27 at D2 to FC = 0.81 (not significant) at D21, with a temporal increase within SCM cows (D2→D21 FC = 4.07) that was absent in healthy cows (FC = 1.35). This SCM-specific temporal recovery parallels the asymmetric amino acid recovery discussed above and further supports the interpretation that catecholamine depletion is an inflammation-specific phenomenon that normalizes during the dry period.

### 4.4. Oxidative Stress, Glutathione Turnover, and Antioxidant Depletion

The oxidative stress signature in SCM quarters was defined by the depletion of hypotaurine (FC = 0.24) and the elevation of L-cysteinylglycine disulfide (FC = 3.10). Hypotaurine is a potent intracellular antioxidant synthesized from cysteine. Its depletion at D2 is consistent with consumption during scavenging of reactive oxygen species (ROS) generated by the neutrophil oxidative burst. L-Cysteinylglycine disulfide is the oxidized product of cysteinylglycine, itself a degradation product of glutathione via gamma-glutamyltranspeptidase. Its elevation indicates active glutathione catabolism—a hallmark of oxidative stress in inflammatory tissue.

These metabolomic findings align with an extensive body of evidence that oxidative stress is a central feature of bovine mastitis pathophysiology. Sordillo and Aitken [56] provided a comprehensive framework for understanding how oxidative stress impairs immune function and promotes tissue damage in dairy cattle, documenting the depletion of antioxidant reserves including glutathione during mastitis. Lauzon et al. [57] demonstrated that glutathione ethyl ester significantly reduced neutrophil-induced cytotoxicity in bovine mammary epithelial cells, establishing a direct link between glutathione availability and mammary tissue protection. At the metabolomic level, several studies have reported perturbations in oxidative metabolism pathways in clinical mastitis milk, while the multifluid metabolomic study by [39] found decreased glutamine and O-phosphocholine in feces of mastitic cows, consistent with systemic oxidative stress.

The antioxidant depletion extended to vitamins: pantothenic acid (vitamin B5; FC = 0.56) and choline (FC = 0.60) were reduced at D2. Pantothenic acid is the precursor of coenzyme A, essential for fatty acid metabolism and acetylation reactions, and its depletion may impair energy metabolism in the inflamed gland. Interestingly, pantothenic acid and choline continued to decline sharply during the D2→D21 transition in both SCM (FC = 0.13 and 0.17, respectively) and H cows (FC = 0.09 and 0.11). This pattern suggests that their temporal decline reflects normal involution-related changes—likely the cessation of milk fat synthesis and membrane remodeling—rather than inflammation per se. In contrast, thiamine (vitamin B1) was elevated at D2 in SCM quarters (FC = 2.80), potentially reflecting release from damaged cells during acute inflammation. The identification of specific vitamin/cofactor perturbations in dry secretion fluid adds a previously uncharacterized dimension to the relationship between antioxidant status and mammary health during the dry period [58,59].

Hypotaurine showed complete recovery by D21 (FC = 1.11), with a temporal increase within SCM cows (FC = 5.49) but not healthy cows (FC = 1.18), paralleling the asymmetric recovery observed for amino acids and catecholamines. This SCM-specific temporal recovery of the antioxidant buffer is consistent with resolution of the oxidative burst as neutrophil infiltration subsides.

### 4.5. The Involution Metabolome: Temporal Dominance and the SCM Overlay

A key contribution of this study is the first metabolomic characterization of the temporal trajectory in bovine dry secretion fluid. In both SCM and healthy quarters, 316 metabolites were significantly altered between D2 and D21—far exceeding the 186 altered between SCM and H at D2. PLS-DA models for the temporal comparisons achieved near-perfect performance (R^2^Y > 0.98, Q^2^ > 0.90, 100% accuracy, AUC = 1.00). PERMANOVA pseudo-F statistics were 10.6–12.3 (*p* = 0.001), substantially exceeding those for health comparisons (F = 1.57–4.74). These results establish that the D2→D21 metabolic shift during mammary involution is the dominant source of variation in dry secretion fluid, exceeding even the impact of subclinical mastitis.

This metabolomic dominance of involution is consistent with transcriptomic and proteomic evidence from several groups. Wang et al. [60] identified over 3300 differentially expressed genes between late lactation and early involution, with upregulated pathways spanning cytoskeleton degradation, cell death, and immune response. At the protein level, Boggs et al. [61] documented changes in 45 milk-protein fragments and 36 host-defence proteins during the first 8 days of drying off, with proteolysis and immune-protein induction as the dominant signatures. Shamay et al. [48] further showed that plasmin and plasminogen activator activities peak during the first three weeks of involution, providing the enzymatic basis for the protein degradation products we observe. Our metabolomic data complement these prior studies by showing that the downstream metabolite landscape mirrors the same biology: progressive accumulation of amino acids (aspartic acid: FC = 6–7 in both groups), tryptophan catabolites (N-acetylindoxyl: FC = 10–16), and protein degradation products.

Of the 316 temporally significant metabolites, 241 (76.3%) were shared between SCM and H cows, indicating that the core involution metabolome is conserved regardless of health status. The shared decline of pantothenic acid and choline in both groups (FC = 0.09–0.17) points to fundamental alterations in CoA biosynthesis and phospholipid metabolism during involution, most likely reflecting the cessation of milk fat synthesis and epithelial cell membrane remodeling [60,62]. The 75 metabolites unique to each temporal comparison included sarcosine (temporal increase uniquely in SCM, FC = 1.65) and homoarginine (temporal increase uniquely in SCM, FC = 2.52)—both of which were depleted at D2 in the health comparison. These SCM-unique temporal metabolites likely represent a recovery component: their trajectory reflects normalization from inflammation-induced depletion rather than involution per se.

### 4.6. Convergence, Candidate Biomarkers, and Practical Implications

The dramatic reduction from 186 to 36 significant metabolites between D2 and D21 (80.6% attenuation), with a 95.2% recovery rate among individual metabolites, demonstrates that the mammary gland’s metabolic landscape largely normalizes during the first three weeks of the dry period, even in quarters with a history of subclinical mastitis. This convergence is further supported by the PLS-DA model metrics: the D21 health comparison (R^2^Y = 0.902, Q^2^ = 0.361, accuracy = 80%) was markedly weaker than the D2 comparison (R^2^Y = 0.924, Q^2^ = 0.697, accuracy = 100%), and PERMANOVA on PCA scores was not significant at D21 (F = 1.57, *p* = 0.084) compared to D2 (F = 4.74, *p* = 0.001).

From a candidate biomarker perspective, six metabolites with the largest effect sizes at D2 merit further investigation: citrulline (FC = 0.26), norepinephrine (FC = 0.27), N-formylkynurenine (FC = 0.27), 4-hydroxyproline (FC = 0.25), hypotaurine (FC = 0.24), and uracil (FC = 8.25). Unlike somatic cell count, which measures cellular infiltration nonspecifically, these metabolites reflect distinct biochemical processes—arginine–NO metabolism, neuroimmune signaling, collagen turnover, antioxidant depletion, and nucleotide salvage—and could potentially distinguish SCM from other causes of elevated SCC. The 100% classification accuracy (AUC = 1.00) of the PLS-DA model at D2 confirms strong discriminatory power, though this must be interpreted cautiously given the small sample size (n = 10 per group). Previous metabolomic biomarker studies in lactating milk have proposed citrate, hippurate, and carnitine as SCM markers [12,14,39,40], but these compounds are influenced by active milk synthesis and may not transfer to the dry-period context. The non-overlapping nature of the biomarker candidates identified in dry secretion fluid versus lactating milk underscores the biochemical distinctiveness of the dry-period mammary gland.

Practically, the convergence by D21 suggests that metabolomic monitoring of dry secretion fluid is most informative in the early dry period. The 11 persistently altered metabolites at D21—which include some dipeptides, proline, and pyrimidine metabolites—warrant further investigation as candidate markers of incomplete resolution that may predict postcalving outcomes. This finding aligns with the concept that the dry period represents a critical window for mammary health, and that incomplete resolution of inflammation during this window may predispose quarters to new infections at calving [4,5]. The binary SCC threshold of 200,000 cells/mL used in this study is internationally standardized, but may obscure dose-dependent metabolic responses. Introducing three SCC subgroups (<200,000; 200,001–400,000; >400,000 cells/mL) directly into the analysis might have benefitted the study. However, the present cohort (n = 10 per group) does not provide sufficient statistical power for a three-way split within the current SCM group—dividing 10 SCM quarters into two or three SCC tiers would yield cells of n = 3–5, precluding meaningful multivariate or univariate analysis. We have therefore conducted the binary classification and recommend that future studies with larger sample sizes (n ≥ 30 per SCC tier) adopt a three-tier SCC stratification (<200,000; 200,001–400,000; >400,000 cells/mL) to fully characterize potential dose–response relationships between SCC and the dry secretion metabolome.

### 4.7. Limitations

Several limitations should be acknowledged. First, the sample size (n = 10 per group) limits statistical power, particularly for the D21 health comparison where only 36 metabolites were significant and the PERMANOVA on PCA scores was not significant (*p* = 0.084). While Benjamini–Hochberg FDR correction was performed as a sensitivity analysis (133/186 metabolites survived at q < 0.05 for D2 health, but only 1/36 for D21 health), the primary analysis used uncorrected *p*-values with the established FC > 1.5 threshold. Larger cohorts are needed to confirm the D21 findings. Second, all samples originated from a single commercial herd. Herd-level factors—genetics, nutrition, management, pathogen profile—may influence the metabolomic response, and multi-herd validation is essential before generalizing these findings. Third, pathogen identification was not performed. SCM was defined by SCC alone, and different pathogens may elicit distinct metabolic responses; Bannerman et al. [39] demonstrated that *Escherichia coli* and *Staphylococcus aureus* elicit markedly different innate immune responses in the bovine mammary gland, and Wang et al. [60] showed pathogen-specific associations between milk microbiota and metabolite profiles in mastitic cows. Fourth, the CIL-LC-MS platform, while providing excellent coverage of amine- and phenol-containing metabolites (474 features), captures only the amine/phenol submetabolome. Lipids, short-chain fatty acids, and sugar metabolites require separate analytical channels and are not represented in this dataset. The dominant perturbations in lactating mastitis milk detected by other platforms—including decreased lactose, citrate, and hippurate [12,14]—could not be assessed here. The dipeptide, amino acid, and tryptophan findings should therefore be interpreted as a partial view of the metabolomic landscape. Fifth, temporal comparisons used unpaired statistical tests despite the paired experimental design (same cow, contralateral quarters). Paired tests could provide greater power but require assumptions about metabolite-level sample independence that may not hold uniformly; a paired sensitivity analysis confirmed that results were robust a paired sensitivity analysis confirmed that results were robust, with the number of significantly altered metabolites remaining essentially unchanged (Supplementary Tables S6 and S7). Sixth, cytological examination of dry secretion samples was not performed. Differential cell counting would have enabled attribution of specific metabolic signatures—particularly the dipeptide accumulation and elevated nucleotide metabolites observed in SCM quarters—to specific immune cell populations such as neutrophils, macrophages, or lymphocytes. Future studies should integrate cytological profiling alongside metabolomics to enable cell-type-resolved mechanistic interpretation. Seventh, the parity range of enrolled cows (1–7) was broad; although first-parity animals showed no systematic separation from multiparous cows in PCA, the influence of parity on dry secretion metabolomics warrants investigation in parity-stratified cohorts. Finally, there was no independent validation cohort. The discriminant metabolites and pathway perturbations identified here should be considered hypothesis-generating until confirmed in larger, multi-herd studies with pathogen-stratified designs and complementary analytical platforms.

## 5. Conclusions

This study provides the first comprehensive metabolomic characterization of bovine dry secretion fluid, establishing baseline metabolic profiles for both healthy and subclinical mastitis-affected mammary quarters during the dry period. Using CIL-LC–MS, 474 metabolites were positively identified, revealing three principal findings. First, subclinical mastitis at the onset of the dry period (D2) imposed a substantial metabolic perturbation, with 186 significantly altered metabolites dominated by dipeptide accumulation and catecholamine depletion. The marked downregulation of norepinephrine (FC = 0.27, *p* = 3.37 × 10^−7^) represents a novel finding that implicates local neuroimmune exhaustion as a feature of subclinical intramammary infection. Second, by D21, the SCM-associated metabolic signature attenuated substantially (36 metabolites), with minimal overlap with the D2 profile, indicating that the metabolic consequences of subclinical mastitis are largely transient within the dry period. Third, and most importantly, temporal changes driven by mammary involution overwhelmed disease-related differences, with over 316 metabolites shifting significantly from D2 to D21 in both health groups. This hierarchy—involution dominates, disease modulates—was confirmed by both unsupervised (PCA) and supervised (PLS-DA) multivariate analyses, and establishes mammary involution as the primary metabolic event shaping the dry secretion environment. The dipeptide-dominated proteolytic signature at D2, the tryptophan–IDO pathway activation, and the persistent oxidative stress markers collectively suggest that SCM quarters undergo an exaggerated inflammatory and catabolic response superimposed on the normal involution program. These metabolic pathways offer candidate targets for interventions aimed at supporting mammary gland health during the dry period beyond conventional antibiotic therapy. Several limitations should be acknowledged. The study used a relatively small sample size (n = 10 per group), pathogen identification was not performed, and bacteriological culture data were not available to distinguish pathogen-specific metabolic signatures. Furthermore, the use of blanket dry-cow therapy means that samples collected at D2 reflect an early post-antibiotic environment, which may have influenced the metabolite profiles observed at that time point. Future studies should validate the candidate markers identified here—particularly norepinephrine, dipeptides, and tryptophan metabolites—in larger, pathogen-characterized cohorts and evaluate their diagnostic utility for on-farm detection of subclinical mastitis at dry-off. Integration of these dry secretion metabolomics data with our previously reported serum and urine metabotyping results may enable the development of multi-compartment diagnostic panels for early identification of cows at risk for persistent intramammary infection across the dry period. Collectively, these findings establish that the dry secretion metabolome is a rich, accessible, and disease-informative matrix: subclinical mastitis superimposes a proteolytic and neuroimmune disruption onto the physiological process of mammary involution, and the candidate markers identified here—particularly dipeptides and norepinephrine at dry-off—provide a compelling basis for the development of rapid, quarter-level diagnostic tools that could transform the management of intramammary infection during the dry period.

## Data Availability

The metabolomics data supporting the findings of this study are not publicly available due to intellectual property restrictions associated with an ongoing research project. Requests for access to the data can be directed to the corresponding author, subject to applicable confidentiality agreements.

## Supplementary Materials

The following supporting information can be downloaded at: https://www.mdpi.com/article/10.3390/vetsci13040345/s1, Figure S1: Permutation Test Validation of PLS-DA Models Across Four Pairwise Comparisons (200 Permutations). Figure S2: ROC Curves for Leave-One-Out Cross-Validated PLS-DA Classification Models Across Four Pairwise Comparisons. Figure S3: Radar Chart of Significantly Altered Metabolites per KEGG Pathway Across Four Pairwise Comparisons. Figure S4: Waterfall Plots of Significantly Altered Metabolites Ranked by log_2_(Fold Change), Color-Coded by KEGG Pathway. Table S1: Summary of pairwise metabolomic comparisons in bovine dry secretions. Table S2: Top 20 metabolites by PLS-DA VIP across all four comparisons. Table S3: Top 20 metabolites by PLS-DA VIP for each individual pairwise comparison: (A) SCM-D2 vs. H-D2; (B) SCM-D21 vs. H-D21; (C) SCM-D21 vs. SCM-D2; (D) H-D21 vs. H-D2. Table S4: All significantly altered metabolites in SCM-D2 vs. H-D2, ranked by *p*-value. Table S5: All significantly altered metabolites in SCM-D21 vs. H-D21, ranked by *p*-value Table S6: Longitudinal changes: SCM-D21 vs. SCM-D2 (paired), ranked by *p*-value. Table S7: Longitudinal changes: H-D21 vs. H-D2 (paired), ranked by *p*-value.

## References

[1] Capuco A.V., Akers R.M., Smith J.J. (2007). Mammary growth in Holstein cows during the dry period: Quantification of nucleic acids and histology. J. Dairy Sci..

[2] Katthöfer P., Zhang Y., Wente N., Preine F., Nitz J., Krömker V. (2024). The influence of milk leakage, udder pressure and further risk factors on the development of new intramammary infections during the dry period of dairy cows. Pathogens.

[3] Ruegg P.L. (2012). New perspectives in udder health management. Vet. Clin. N. Am. Food Anim. Pract..

[4] Bradley A.J., Green M.J. (2004). The importance of the nonlactating period in the epidemiology of intramammary infection and strategies for prevention. Vet. Clin. N. Am. Food Anim. Pract..

[5] Green M.J., Green L.E., Medley G.F., Schukken Y.H., Bradley A.J. (2002). Influence of dry period bacterial intramammary infection on clinical mastitis in dairy cows. J. Dairy Sci..

[6] Ruegg P.L. (2017). A 100-year review: Mastitis detection, management, and prevention. J. Dairy Sci..

[7] Halasa T., Huijps K., Østerås O., Hogeveen H. (2007). Economic effects of bovine mastitis and mastitis management: A review. Vet. Q..

[8] Canadian Dairy Information Centre (CDIC) Culling and Replacement Rates in Dairy Herds in Canada. https://agriculture.canada.ca/en/sector/animal-industry/canadian-dairy-information-centre/statistics-market-information/dairy-animal-genetics/culling-replacement.

[9] Dervishi E., Zhang G., Hailemariam D., Dunn S.M., Ametaj B.N. (2015). Innate immunity and carbohydrate metabolism alterations precede occurrence of subclinical mastitis in transition dairy cows. J. Anim. Sci. Technol..

[10] Dervishi E., Zhang G., Dunn S.M., Mandal R., Wishart D.S., Ametaj B.N. (2017). GC–MS metabolomics identifies metabolite alterations that precede subclinical mastitis in the blood of transition dairy cows. J. Proteome Res..

[11] Zwierzchowski G., Zhang G., Mandal R., Wishart D.S., Ametaj B.N. (2020). Mass-spec-based urinary metabotyping around parturition identifies screening biomarkers for subclinical mastitis in dairy cows. Res. Vet. Sci..

[12] Sundekilde U.K., Poulsen N.A., Larsen L.B., Bertram H.C. (2013). Nuclear magnetic resonance metabonomics reveals strong association between milk metabolites and somatic cell count in bovine milk. J. Dairy Sci..

[13] Tong J., Zhang H., Zhang Y., Xiong B., Jiang L. (2019). Microbiome and metabolome analyses of milk from dairy cows with subclinical *Streptococcus agalactiae* mastitis—Potential biomarkers. Front. Microbiol..

[14] Xi X., Kwok L.Y., Wang Y., Ma C., Mi Z., Zhang H. (2017). Ultra-performance liquid chromatography-quadrupole-time of flight mass spectrometry MSE-based untargeted milk metabolomics in dairy cows with subclinical or clinical mastitis. J. Dairy Sci..

[15] Hettinga K.A., Van Valenberg H.J.F., Lam T.J.G.M., Van Hooijdonk A.C.M. (2008). Detection of mastitis pathogens by analysis of volatile bacterial metabolites. J. Dairy Sci..

[16] Wishart D.S. (2019). Metabolomics for investigating physiological and pathophysiological processes. Physiol. Rev..

[17] Huan T., Li L. (2015). Quantitative metabolome analysis based on chromatographic peak reconstruction in chemical isotope labeling liquid chromatography mass spectrometry. Anal. Chem..

[18] Huang Y., Shen L., Jiang J., Xu Q., Luo Z., Luo Q., Yu S., Yao X., Ren Z., Hu Y. (2019). Metabolomic profiles of bovine mammary epithelial cells stimulated by lipopolysaccharide. Sci. Rep..

[19] Edmonson A.J., Lean I.J., Weaver L.D., Farver T., Webster G. (1989). A body condition scoring chart for Holstein dairy cows. J. Dairy Sci..

[20] CCAC (2009). CCAC Guidelines on: The Care and Use of Farm Animals in Research.

[21] Schukken Y.H., Wilson D.J., Welcome F., Garrison-Tikofsky L., Gonzalez R.N. (2003). Monitoring udder health and milk quality using somatic cell counts. Vet. Res..

[22] Sargeant J.M., Leslie K.E., Shirley J.E., Pulkrabek B.J., Lim G.H. (2001). Sensitivity and specificity of somatic cell count and California Mastitis Test for identifying intramammary infection in early lactation. J. Dairy Sci..

[23] Zhao S., Li H., Han W., Chan W., Li L. (2019). Metabolomic Coverage of Chemical-Group-Submetabolome Analysis: Group Classification and 4-Channel Chemical Isotope Labeling LC-MS. Anal. Chem..

[24] Zhou R., Tseng C.-L., Huan T., Li L. (2014). IsoMS: Automated processing of LC-MS data generated by a chemical isotope labeling metabolomics platform. Anal. Chem..

[25] Huan T., Li L. (2015). Counting missing values in a metabolite-intensity data set for measuring the analytical performance of a metabolomics platform. Anal. Chem..

[26] Sumner L.W., Amberg A., Barrett D., Beale M.H., Beger R., Daykin C.A., Fan T.W.-M., Fiehn O., Goodacre R., Griffin J.L. (2007). Proposed minimum reporting standards for chemical analysis Chemical Analysis Working Group (CAWG) Metabolomics Standards Initiative (MSI). Metabolomics.

[27] Pang Z., Lu Y., Zhou G., Hui F., Xu L., Viau C., Gavber A.F., Ha B., Bhavsar S., Xia J. (2024). MetaboAnalyst 6.0: Towards a unified platform for metabolomics data processing, analysis and interpretation. Nucleic Acids Res..

[28] Li L., Li R., Zhou J., Zuniga A., Stanislaus A.E., Wu Y., Huan T., Zheng J., Shi Y., Wishart D.S. (2013). MyCompoundID: Using an evidence-based metabolome library for metabolite identification. Anal. Chem..

[29] Guo K., Li L. (2009). Differential ^12^C-/^13^C-isotope dansylation labeling and fast liquid chromatography/mass spectrometry for absolute and relative quantification of the metabolome. Anal. Chem..

[30] Szymanska E., Saccenti E., Smilde A.K., Westerhuis J.A. (2012). Double-check: Validation of diagnostic statistics for PLS-DA models in metabolomics studies. Metabolomics.

[31] Phipson B., Smyth G.K. (2010). Permutation p-values should never be zero: Calculating exact p-values when permutations are randomly drawn. Stat. Appl. Genet. Mol. Biol..

[32] Chong I.-G., Jun C.-H. (2005). Performance of some variable selection methods when multicollinearity is present. Chemom. Intell. Lab. Syst..

[33] Saccenti E., Hoefsloot H.C.J., Smilde A.K., Westerhuis J.A., Hendriks M.M.W.B. (2014). Reflections on univariate and multivariate analysis of metabolomics data. Metabolomics.

[34] Goeman J.J., van de Geer S.A., de Kort F., van Houwelingen H.C. (2004). A global test for groups of genes: Testing association with a clinical outcome. Bioinformatics.

[35] Kanehisa M., Goto S. (2000). KEGG: Kyoto encyclopedia of genes and genomes. Nucleic Acids Res..

[36] Pedregosa F., Varoquaux G., Gramfort A., Michel V., Thirion B., Grisel O., Blondel M., Prettenhofer P., Weiss R., Dubourg V. (2011). Scikit-learn: Machine learning in Python. J. Mach. Learn. Res..

[37] Virtanen P., Gommers R., Oliphant T.E., Haberland M., Reddy T., Cournapeau D., Burovski E., Peterson P., Weckesser W., Bright J. (2020). SciPy 1.0: Fundamental algorithms for scientific computing in Python. Nat. Methods.

[38] Hunter J.D. (2007). Matplotlib: A 2D graphics environment. Comput. Sci. Eng..

[39] Wang Y., Nan X., Zhao Y., Jiang L., Wang H., Zhang F., Hua D., Liu J., Yang L., Yao J. (2020). Coupling 16S rDNA sequencing and untargeted mass spectrometry for milk microbial composition and metabolites from dairy cows with clinical and subclinical mastitis. J. Agric. Food Chem..

[40] Zhu C., Zhao Y., Yang F., Zhang Q., Zhao X., Yang Z., Dao X., Laghi L. (2024). Microbiome and metabolome analyses of milk and feces from dairy cows with healthy, subclinical, and clinical mastitis. Front. Microbiol..

[41] Prin-Mathieu C., Le Roux Y., Faure G.C., Laurent F., Béné M.C., Moussaoui F. (2002). Enzymatic activities of bovine peripheral blood leukocytes and milk polymorphonuclear neutrophils during intramammary inflammation caused by lipopolysaccharide. Clin. Diagn. Lab. Immunol..

[42] Hinz K., Larsen L.B., Wellnitz O., Bruckmaier R.M., Kelly A.L. (2012). Proteolytic and proteomic changes in milk at quarter level following infusion with Escherichia coli lipopolysaccharide. J. Dairy Sci..

[43] Le Roux Y., Laurent F., Moussaoui F. (2003). Polymorphonuclear proteolytic activity and milk composition change. Vet. Res..

[44] Thomas F.C., Mullen W., Tassi R., Ramírez-Torres A., Mudaliar M., McNeilly T.N., Zadoks R.N., Burchmore R., Eckersall P.D. (2016). Mastitomics, the integrated omics of bovine milk in an experimental model of *Streptococcus uberis* mastitis: 3. Untargeted metabolomics. Mol. BioSyst..

[45] Bannerman D.D., Paape M.J., Lee J.W., Zhao X., Hope J.C., Rainard P. (2004). *Escherichia coli* and *Staphylococcus aureus* elicit differential innate immune responses following intramammary infection. Clin. Vaccine Immunol..

[46] Rainard P., Riollet C. (2006). Innate immunity of the bovine mammary gland. Vet. Res..

[47] Mehrzad J., Desrosiers C., Lauzon K., Robitaille G., Zhao X., Lacasse P. (2005). Proteases involved in mammary tissue damage during endotoxin-induced mastitis in dairy cows. J. Dairy Sci..

[48] Shamay A., Homans R., Fuerman Y., Levin I., Barash H., Silanikove N., Mabjeesh S.J. (1997). Proteolysis of milk proteins during involution of the bovine mammary gland. J. Dairy Sci..

[49] Cervenka I., Agudelo L.Z., Ruas J.L. (2017). Kynurenines: Tryptophan’s metabolites in exercise, inflammation, and mental health. Science.

[50] Moffett J.R., Namboodiri M.A. (2003). Tryptophan and the immune response. Immunol. Cell Biol..

[51] Bochniarz M., Kocki T., Dąbrowski R., Szczubiał M., Wawron W., Turski W.A. (2018). Tryptophan, kynurenine, kynurenic acid concentrations and indoleamine 2,3-dioxygenase activity in serum and milk of dairy cows with subclinical mastitis caused by coagulase-negative staphylococci. Reprod. Domest. Anim..

[52] Bochniarz M., Dąbrowski R., Kocki T., Błaszczyk P., Szczubiał M., Brodzki P., Krakowski L., Turski W.A. (2022). Content of tryptophan and kynurenines in serum and milk of dairy cows with mastitis caused by *Streptococcus* spp.. Reprod. Domest. Anim..

[53] Bochniarz M., Piech T., Kocki T., Iskra M., Krukowski H., Jagielski T. (2021). Tryptophan, kynurenine and kynurenic acid concentrations in milk and serum of dairy cows with *Prototheca* mastitis. Animals.

[54] Sternberg E.M. (2006). Neural regulation of innate immunity: A coordinated nonspecific host response to pathogens. Nat. Rev. Immunol..

[55] Madden K.S., Felten S.Y., Felten D.L., Sundaresan P.R., Livnat S. (1989). Sympathetic neural modulation of the immune system. I. Depression of T cell immunity in vivo and in vitro following chemical sympathectomy. Brain Behav. Immun..

[56] Sordillo L.M., Aitken S.L. (2009). Impact of oxidative stress on the health and immune function of dairy cattle. Vet. Immunol. Immunopathol..

[57] Lauzon K., Zhao X., Bouetard A., Delbecchi L., Paquette B., Bhaskara Lacasse P. (2005). Antioxidants to prevent bovine neutrophil-induced mammary epithelial cell damage. J. Dairy Sci..

[58] Sordillo L.M. (2016). Nutritional strategies to optimize dairy cattle immunity. J. Dairy Sci..

[59] Pascottini O.B., Leroy J.L.M.R., Opsomer G. (2020). Metabolic stress in the transition period of dairy cows: Focusing on the prepartum period. Animals.

[60] Dado-Senn B., Skibiel A.L., Fabris T.F., Zhang Y., Dahl G.E., Peñagaricano F., Laporta J. (2018). RNA-Seq reveals novel genes and pathways involved in bovine mammary involution during the dry period and under environmental heat stress. Sci. Rep..

[61] Boggs I., Hine B., Smolenski G., Hettinga K., Zhang L., Wheeler T.T. (2016). Proteomics data in support of the quantification of the changes of bovine milk proteins during mammary gland involution. Data Brief.

[62] Capuco A.V., Akers R.M. (2009). The origin and evolution of lactation. J. Biol..

