# Supplementary material for: The Dry Secretion Metabolome: LC-MS Profiling Distinguishes Subclinical Mastitis from Healthy Udder Quarters Across the Dry Period in Dairy Cows
Source: Vet Sci. 2026 Apr 2;13(4):345. doi: 10.3390/vetsci13040345 (PMC13120032; doi:10.3390/vetsci13040345)
Supplement: Supplementary file 1 [file vetsci-13-00345-s001.zip › Supplementary Tables S1-S7.pdf]

Supplementary Tables

The Dry Secretion Metabolome: LC-MS Profiling Distinguishes Subclinical Mastitis from Healthy Udder Quarters Across the Dry Period in Dairy Cows

High Performance Chemical Isotope Labeling LC-MS (TMIC028P)

Table S1: Summary of pairwise metabolomic comparisons in bovine dry secretions.

Table S2: Top 20 metabolites by PLS-DA VIP score across all four groups (4-group model).

Table S3: Top 20 metabolites by PLS-DA VIP score for each individual pairwise comparison (A–D).

Table S4: All significantly altered metabolites in SCM-D2 vs H-D2 (n = 186), ranked by p-value.

Table S5: All significantly altered metabolites in SCM-D21 vs H-D21 (n = 36), ranked by p-value.

Table S6: Longitudinal changes: SCM-D21 vs SCM-D2 (n = 316), ranked by p-value.

Table S7: Longitudinal changes: H-D21 vs H-D2 (n = 316), ranked by p-value.

Abbreviations: SCM = Subclinical Mastitis (HSCC); H = Healthy; D2 = Day 2 of dry period; D21 = Day 21 of dry period. FC = Fold Change. FDR = False Discovery Rate (Benjamini–Hochberg). VIP = Variable Importance in Projection. PLS-DA = Partial Least Squares Discriminant Analysis. KEGG = Kyoto Encyclopedia of Genes and Genomes. n = 10 cows per group; 474 total metabolites (228 Tier 1 + 246 Tier 2).

Table S1. Summary of pairwise metabolomic comparisons in bovine dry secretions (Welch's t-test, $p < 0.05$, FC > 1.5 or < 1/1.5; n = 10 per group; 474 metabolites).

Comparison	Total sig.	Up (↑)	Down (↓)	VIP > 1 (PLS-DA)	Survive FDR (q<0.05)	% of 474 metabolites
SCM-D2 vs H-D2	174	105	69	228	133	36.7%
SCM-D21 vs H-D21	35	25	10	177	1	7.4%
SCM-D21 vs SCM-D2	301	197	104	246	293	63.5%
H-D21 vs H-D2	306	212	94	246	311	64.6%

Significance criteria: Welch's two-tailed t-test, $p < 0.05$, |FC| > 1.5. FDR: Benjamini–Hochberg correction ($q < 0.05$). VIP > 1: metabolites exceeding threshold from corresponding PLS-DA model. % of 474: proportion of the total detected metabolome significantly altered. Abbreviations: SCM = Subclinical Mastitis; H = Healthy; D2 = Day 2; D21 = Day 21.

Table S2. Top 20 metabolites by PLS-DA VIP score across all four groups (4-group model; 3 components; 474 metabolites; n = 10 per group). Dir. = direction relative to SCM vs H overall mean.

Rank	Metabolite	Pathway	VIP	Dir.
1	3-Cyano-Alanine	Alanine, aspartate & glutamate metabolism	1.911	↑
2	Leucyl-Proline	Protein digestion & absorption	1.585	↑
3	Isomer 2 of 3-Hydroxymelatonin	Amino acid metabolism	1.556	↑
4	Cytidine	Amino acid metabolism	1.554	↓
5	4-O-Methylgallic acid	Protein digestion & absorption	1.545	↓
6	Isomer 1 of 3-Hydroxymelatonin	Amino acid metabolism	1.518	↑
7	5-Methoxytryptophan	Protein digestion & absorption	1.502	↑
8	Norsalsolinol	Amino acid metabolism	1.497	↑
9	3-O-Methylgallic Acid	Protein digestion & absorption	1.484	↓
10	Salsolinol 1-carboxylic acid	Amino acid metabolism	1.442	↓
11	N-Acetyl-Dihydrofolic Acid	Amino acid metabolism	1.440	↑
12	Prolyl-Proline	Protein digestion & absorption	1.431	↑
13	4-Hydroxy-4-methylglutamic acid	Amino acid metabolism	1.426	↓
14	Prolyl-Lysine	Protein digestion & absorption	1.424	↑
15	Proline	Amino acid metabolism	1.409	↑
16	Beta-Alanine	Alanine, aspartate & glutamate metabolism	1.406	↓
17	Leucyl-Asparagine	Protein digestion & absorption	1.401	↑
18	Aspartyl-Methionine	Protein digestion & absorption	1.385	↑
19	Homoarginine	Amino acid metabolism	1.385	↓
20	Phenylalanyl-Threonine	Protein digestion & absorption	1.380	↑

VIP scores extracted from PLS-DA models (2 components per pairwise comparison; 3 components for 4-group model). Metabolites with VIP > 1 are considered important discriminators. Dir. = direction of change (first-named group vs second). FC and p-value from Welch's t-test. Pathway annotations based on KEGG Bos taurus metabolic library.

Table S3. Top 20 metabolites by PLS-DA VIP score for each individual pairwise comparison (A–D). Two-component models; n = 10 per group; 474 metabolites.

SCM-D2 vs H-D2 (A)					
**Rank**	**Metabolite**	**Pathway**	**VIP**	**FC**	**p-value**
1	Isomer 1 of Lysyl-Glutamate	Protein digestion & absorption	1.855	2.783	2.69E−5
2	Glutaminyl-Proline/Prolyl-Glutamine	Protein digestion & absorption	1.774	0.621	2.16E−3
3	Phenylalanyl-Threonine	Protein digestion & absorption	1.732	4.154	1.35E−6
4	Ascorbic acid	Amino acid metabolism	1.703	1.336	3.84E−2
5	Tyrosyl-Proline	Protein digestion & absorption	1.682	1.916	4.90E−4
6	2(N)-Methyl-norsalsolinol	Protein digestion & absorption	1.634	1.837	9.89E−3
7	Leucyl-Asparagine	Protein digestion & absorption	1.592	2.411	1.94E−5
8	Methylcysteine	Protein digestion & absorption	1.582	0.253	1.96E−5
9	2'-Aminobiphenyl-2,3-diol	Amino acid metabolism	1.578	1.814	1.48E−2
10	N5-Acetyl-Ornithine	Amino acid metabolism	1.523	0.416	6.24E−5
11	Creatine	Amino acid metabolism	1.518	0.399	1.73E−5
12	Deoxyeritadenine	Amino acid metabolism	1.511	0.523	6.30E−5
13	4-Hydroxynorephedrine	Amino acid metabolism	1.511	0.363	3.70E−5
14	Citrulline	Arginine & proline metabolism	1.508	0.256	3.60E−5
15	Formyl-5-Hydroxykynurenamine	Amino acid metabolism	1.503	0.604	3.01E−3
16	(3S,5S)-3,5-Diaminohexanoic acid2	Amino acid metabolism	1.500	0.688	1.48E−1
17	Leucyl-Glutamine	Protein digestion & absorption	1.499	3.092	3.52E−5
18	Norepinephrine	Tyrosine metabolism	1.495	0.271	3.96E−5
19	Homoarginine	Amino acid metabolism	1.490	0.264	3.65E−5
20	4-Methylene-Glutamine	Protein digestion & absorption	1.477	0.268	5.81E−5
**SCM-D21 vs H-D21 (B)**					
**Rank**	**Metabolite**	**Pathway**	**VIP**	**FC**	**p-value**
1	3-Cyano-Alanine	Alanine, aspartate & glutamate metabolism	2.589	1.596	2.33E−5
2	Glutamine	Amino acid metabolism	2.132	0.752	1.49E−3

3	Cytidine	Amino acid metabolism	2.056	0.551	2.17E−3
4	Leucyl-Proline	Protein digestion & absorption	2.046	1.929	1.86E−3
5	Isomer 2 of 3-Hydroxymelatonin	Amino acid metabolism	1.953	1.752	3.51E−3
6	Prolyl-Valine	Protein digestion & absorption	1.953	1.838	4.31E−3
7	4-O-Methylgallic acid	Protein digestion & absorption	1.933	0.545	6.01E−3
8	Phenol	Amino acid metabolism	1.857	1.859	7.46E−3
9	N-Acetyl-Dihydrofolic Acid	Amino acid metabolism	1.854	1.684	6.92E−3
10	3-O-Methylgallic Acid	Protein digestion & absorption	1.853	0.538	1.04E−2
11	5-Methoxytryptophan	Protein digestion & absorption	1.849	2.702	1.37E−2
12	Phenylalanyl-Alanine	Alanine, aspartate & glutamate metabolism	1.837	1.818	9.16E−3
13	Norsalsolinol	Amino acid metabolism	1.836	2.083	6.86E−3
14	4-Chloro-L-lysine	Amino acid metabolism	1.832	0.599	8.62E−3
15	Prolyl-Proline	Protein digestion & absorption	1.803	2.719	1.93E−2
16	N-Acetyl-N-Methylserotonin	Protein digestion & absorption	1.786	1.605	9.85E−3
17	5-Aminopentanamide	Amino acid metabolism	1.778	1.600	9.28E−3
18	Proline	Amino acid metabolism	1.772	1.699	1.36E−2
19	1-Methylguanosine	Protein digestion & absorption	1.770	1.343	1.85E−2
20	3,4-Dihydroxyphenylethyleneglycol 4-O-glucuronide	Amino acid metabolism	1.764	0.335	2.83E−2
**SCM-D21 vs SCM-D2 (C)**					
**Rank**	**Metabolite**	**Pathway**	**VIP**	**FC**	**p-value**
1	Isomer 1 of 4-Chloro-L-lysine	Amino acid metabolism	1.537	0.134	2.53E−10
2	1-Methylguanosine	Protein digestion & absorption	1.517	3.527	1.62E−9
3	Aspartic acid	Alanine, aspartate & glutamate metabolism	1.516	6.127	1.66E−9
4	4-Hydroxyphenylglyoxylic Acid	Amino acid metabolism	1.514	0.150	2.19E−9
5	Asparaginyl-Serine	Protein digestion & absorption	1.511	3.156	2.61E−9
6	Arginyl-Leucine	Protein digestion & absorption	1.499	0.124	6.07E−9
7	4-Oxoproline	Amino acid metabolism	1.498	5.742	6.64E−9
8	S-Adenosyl-L-homocysteine	Amino acid metabolism	1.490	0.193	1.14E−8

9	N-Acetylindoxyl	Tryptophan metabolism	1.490	10.453	1.13E−8
10	Nocardicin G	Amino acid metabolism	1.488	0.220	1.38E−8
11	Beta-Ethynylserine	Amino acid metabolism	1.480	5.261	2.08E−8
12	Tyrosyl-Valine	Protein digestion & absorption	1.480	0.195	2.58E−8
13	Amoxicillin	Amino acid metabolism	1.479	0.214	2.30E−8
14	Phenylalanyl-Glycine	Glycine, serine & threonine metabolism	1.476	0.250	2.84E−8
15	Pantothenic Acid	Pantothenate & CoA biosynthesis	1.475	0.129	2.86E−8
16	Coutaric acid	Amino acid metabolism	1.472	0.307	6.27E−8
17	Isomer 1 of Iminodiacetic acid	Amino acid metabolism	1.469	2.922	4.09E−8
18	1-Aminocyclopropane-1-Carboxylic Acid	Amino acid metabolism	1.463	3.861	5.70E−8
19	Alanine	Alanine, aspartate & glutamate metabolism	1.461	6.371	7.67E−8
20	Valyl-Lysine	Protein digestion & absorption	1.461	0.262	7.51E−8
**H-D21 vs H-D2 (D)**					
**Rank**	**Metabolite**	**Pathway**	**VIP**	**FC**	**p-value**
1	N-Acetyl-3-Hydroxyanthranilic acid	Tryptophan metabolism	1.533	0.148	2.68E−14
2	7,8-Dihydroxykynurenic acid	Tryptophan metabolism	1.516	4.052	2.12E−12
3	Lysyl-Valine	Protein digestion & absorption	1.515	0.156	1.77E−12
4	Valyl-Lysine	Protein digestion & absorption	1.508	0.139	6.50E−12
5	Leucine	Amino acid metabolism	1.496	5.821	3.65E−11
6	3,4-Dihydroxystyrene	Amino acid metabolism	1.495	8.330	5.51E−11
7	Aspartic acid	Alanine, aspartate & glutamate metabolism	1.493	7.073	5.42E−11
8	alpha-D-Glucosamine 1-phosphate	Protein digestion & absorption	1.488	0.220	1.04E−10
9	Isomer 1 of 4-Chloro-L-lysine	Amino acid metabolism	1.476	0.145	3.76E−10
10	Octopamine	Amino acid metabolism	1.474	4.464	4.95E−10
11	Methionine	Amino acid metabolism	1.470	4.292	6.54E−10
12	Seryl-Valine	Protein digestion & absorption	1.464	3.084	1.19E−9
13	Protocatechuic Acid	Amino acid metabolism	1.463	6.283	1.34E−9
14	S-Cysteinosuccinic acid	Protein digestion & absorption	1.462	0.223	1.33E−9

15	Pantothenic Acid	Pantothenate & CoA biosynthesis	1.460	0.087	1.58E−9
16	Phenylalanine	Alanine, aspartate & glutamate metabolism	1.458	5.000	1.96E−9
17	4-Hydroxystyrene	Amino acid metabolism	1.456	7.559	2.46E−9
18	L-Isoleucine	Amino acid metabolism	1.450	4.483	3.98E−9
19	Isomer 1 of p-Hydroxyphenylacetylglycine	Glycine, serine & threonine metabolism	1.442	0.305	6.62E−9
20	Diethanolamine	Amino acid metabolism	1.438	0.163	9.22E−9

VIP scores extracted from PLS-DA models (2 components per pairwise comparison; 3 components for 4-group model). Metabolites with VIP > 1 are considered important discriminators. Dir. = direction of change (first-named group vs second). FC and p-value from Welch's t-test. Pathway annotations based on KEGG Bos taurus metabolic library.

Table S4. All significantly altered named metabolites in SCM-D2 vs H-D2 (n = 186), ranked by ascending p-value (Welch's t-test, $p < 0.05$, FC > 1.5 or < 1/1.5).

Rank	Dir.	Metabolite	Pathway	FC	$\log_2$(FC)	p-value	$-\log_{10}$(p)
1	↓	Norepinephrine	Tyrosine metabolism	0.2707	-1.885	**3.37E−7**	6.472
2	↓	Citrulline	Arginine & proline metabolism	0.2556	-1.968	**1.04E−6**	5.985
3	↑	Leucyl-Asparagine	Alanine, aspartate & glutamate metabolism	2.4112	1.270	**1.15E−6**	5.941
4	↓	3-Aminoisobutanoic acid	Amino acid metabolism	0.2329	-2.102	**1.40E−6**	5.855
5	↓	Methylcysteine	Cysteine & methionine metabolism	0.2534	-1.981	**2.51E−6**	5.600
6	↑	Phenylalanyl-Threonine	Glycine, serine & threonine metabolism	4.1541	2.054	**2.65E−6**	5.577
7	↓	4-Hydroxynorephedrine	Amino acid metabolism	0.3628	-1.463	**2.67E−6**	5.574
8	↓	4-Methylene-Glutamine	Alanine, aspartate & glutamate metabolism	0.2683	-1.898	**3.36E−6**	5.474
9	↑	Valyl-Arginine	Protein digestion & absorption	7.1094	2.830	**3.62E−6**	5.441
10	↓	4-Hydroxyproline	Arginine & proline metabolism	0.2526	-1.985	**3.77E−6**	5.424
11	↓	(2R,3R,4R)-2-Amino-4-hydroxy-3-methylpentanoic acid	Amino acid metabolism	0.3077	-1.700	**4.24E−6**	5.372
12	↑	Isomer 1 of Lysyl-Glutamate	Alanine, aspartate & glutamate metabolism	2.7830	1.477	**4.71E−6**	5.327
13	↓	Homoarginine	Arginine & proline metabolism	0.2642	-1.920	**5.31E−6**	5.274
14	↑	Leucyl-Glutamine	Alanine, aspartate & glutamate metabolism	3.0919	1.628	**5.71E−6**	5.243
15	↑	Arginyl-Valine	Valine, leucine & isoleucine metabolism	5.6930	2.509	**5.84E−6**	5.234
16	↓	5-Hydroxylysine	Lysine degradation	0.2003	-2.319	**9.44E−6**	5.025
17	↓	Deoxyeritadenine	Amino acid metabolism	0.5231	-0.935	**2.01E−5**	4.696
18	↓	Beta-Alanine	Alanine, aspartate & glutamate metabolism	0.3008	-1.733	**2.24E−5**	4.650
19	↑	Leucyl-Threonine	Glycine, serine & threonine metabolism	3.2673	1.708	**2.85E−5**	4.546
20	↑	di-Hydroxymelatonin	Amino acid metabolism	2.5417	1.346	**3.03E−5**	4.519
21	↑	1,4-Diaminobutane	Amino acid metabolism	3.4919	1.804	**3.29E−5**	4.483
22	↓	Isomer 1 of N(6)-Methyllysine	Lysine degradation	0.2735	-1.870	**3.36E−5**	4.474
23	↑	2'-Aminoacetophenone	Amino acid metabolism	2.5355	1.342	**3.90E−5**	4.409
24	↑	p-Coumaroylputrescine	Amino acid metabolism	2.5553	1.354	**5.22E−5**	4.282

Rank	Dir.	Metabolite	Pathway	FC	$\log_2$(FC)	p-value	$-\log_{10}$(p)
25	↑	4-Hydroxybenzaldehyde/3-Hydroxybenzaldehyde	Tyrosine metabolism	5.0158	2.326	**5.41E−5**	4.267
26	↓	Creatine	Arginine & proline metabolism	0.3990	-1.326	**5.43E−5**	4.265
27	↓	N-formylkynurenine	Tryptophan metabolism	0.2695	-1.892	**5.50E−5**	4.259
28	↓	Sarcosine	Glycine, serine & threonine metabolism	0.3706	-1.432	**7.09E−5**	4.149
29	↓	N5-Acetyl-Ornithine	Arginine & proline metabolism	0.4159	-1.266	**8.56E−5**	4.068
30	↓	Symmetric dimethylarginine	Amino acid metabolism	0.3771	-1.407	**8.60E−5**	4.066
31	↑	Glycyl-Leucine	Valine, leucine & isoleucine metabolism	3.4310	1.779	**8.70E−5**	4.061
32	↑	Lysyl-Threonine	Glycine, serine & threonine metabolism	3.7761	1.917	**9.68E−5**	4.014
33	↓	(2R,4S)-2,4-Diaminopentanoic Acid	Amino acid metabolism	0.3241	-1.626	**1.07E−4**	3.971
34	↑	Seryl-Lysine	Lysine degradation	3.2968	1.721	**1.11E−4**	3.955
35	↑	3-Hydroxymonoethylglycinexylidide	Glycine, serine & threonine metabolism	2.3513	1.234	**1.13E−4**	3.946
36	↓	Hypotaurine	Taurine & hypotaurine metabolism	0.2371	-2.076	**1.19E−4**	3.924
37	↓	Glutamine	Alanine, aspartate & glutamate metabolism	0.4538	-1.140	**1.24E−4**	3.907
38	↓	4-Aminocatechol	Amino acid metabolism	0.2649	-1.916	**1.32E−4**	3.880
39	↑	Aspartyl-Methionine	Cysteine & methionine metabolism	2.3243	1.217	**1.34E−4**	3.872
40	↑	Epsilon-(Gamma-Glutamyl)-Lysine	Lysine degradation	2.4092	1.268	**1.35E−4**	3.869
41	↑	Thiamine	Amino acid metabolism	2.7991	1.485	**1.43E−4**	3.846
42	↑	Seryl-Isoleucine	Valine, leucine & isoleucine metabolism	2.8591	1.516	**1.53E−4**	3.815
43	↑	Glutamyl-Leucine	Valine, leucine & isoleucine metabolism	2.2210	1.151	**1.61E−4**	3.793
44	↑	Lysyl-Alanine	Alanine, aspartate & glutamate metabolism	2.6881	1.427	**1.77E−4**	3.753
45	↑	Prolyl-Isoleucine	Valine, leucine & isoleucine metabolism	3.3600	1.748	**1.80E−4**	3.746
46	↑	Threoninyl-Glutamate	Alanine, aspartate & glutamate metabolism	2.4338	1.283	**2.23E−4**	3.652
47	↑	Histidinyl-Glutamine	Alanine, aspartate & glutamate metabolism	3.2816	1.714	**2.34E−4**	3.631
48	↑	5-Aminopentanamide	Amino acid metabolism	3.0613	1.614	**2.43E−4**	3.615
49	↑	2,3-Dihydroxyindole	Amino acid metabolism	2.9271	1.550	**2.72E−4**	3.566
50	↓	Ornithine	Arginine & proline metabolism	0.3464	-1.530	**2.77E−4**	3.558

Rank	Dir.	Metabolite	Pathway	FC	$\log_2$(FC)	p-value	$-\log_{10}$(p)
51	↑	L-Cysteinylglycine disulfide	Glutathione metabolism	3.1023	1.633	**2.89E−4**	3.539
52	↑	5-Hydroxykynurenamine	Tryptophan metabolism	3.2798	1.714	**2.93E−4**	3.534
53	↓	Isomer 1 of gamma-Amino-gamma-cyanobutanoic acid	Amino acid metabolism	0.5277	-0.922	**3.31E−4**	3.480
54	↓	Cystine	Cysteine & methionine metabolism	0.4699	-1.090	**3.36E−4**	3.473
55	↓	Alanine	Alanine, aspartate & glutamate metabolism	0.3642	-1.457	**3.43E−4**	3.465
56	↓	Uric acid	Purine metabolism	0.1989	-2.330	**3.61E−4**	3.442
57	↑	2-(3-Carboxy-3-Aminopropyl)-L-Histidine	Histidine metabolism	2.4801	1.310	**3.71E−4**	3.431
58	↓	(S)-2-Aminobutanoic Acid	Amino acid metabolism	0.5476	-0.869	**3.77E−4**	3.423
59	↑	N-Acetylputrescine	Amino acid metabolism	1.8246	0.868	**3.84E−4**	3.415
60	↓	Pantothenic Acid	Pantothenate & CoA biosynthesis	0.5609	-0.834	**4.72E−4**	3.326
61	↑	O-Acetyl-Homoserine	Glycine, serine & threonine metabolism	1.6328	0.707	**5.06E−4**	3.295
62	↓	Isomer 3 of 5-Aminopentanoic acid	Amino acid metabolism	0.5078	-0.978	**5.10E−4**	3.292
63	↑	5,6-Dihydroxyindole	Amino acid metabolism	2.4558	1.296	**5.18E−4**	3.285
64	↑	Isoleucyl-Alanine	Alanine, aspartate & glutamate metabolism	2.2417	1.165	**5.67E−4**	3.246
65	↑	Alanyl-Glutamic Acid	Protein digestion & absorption	2.6862	1.426	**6.04E−4**	3.219
66	↑	Arginyl-Phenylalanine	Phenylalanine metabolism	3.1622	1.661	**6.31E−4**	3.200
67	↓	Pipecolic Acid	Amino acid metabolism	0.5160	-0.955	**6.54E−4**	3.184
68	↑	3,4-Dihydroxyphenylacetaldehyde	Amino acid metabolism	2.1273	1.089	**7.47E−4**	3.126
69	↑	Uracil	Pyrimidine metabolism	8.2465	3.044	**8.25E−4**	3.083
70	↑	Threoninyl-Lysine	Lysine degradation	2.4977	1.321	**8.81E−4**	3.055
71	↑	1H-Indole-3-methanamine	Amino acid metabolism	1.9867	0.990	**9.85E−4**	3.006
72	↑	N-gamma-Glutamyl-S-allylcysteine	Protein digestion & absorption	1.8518	0.889	1.05E−3	2.980
73	↑	Isoleucyl-Isoleucine	Valine, leucine & isoleucine metabolism	3.0190	1.594	1.07E−3	2.969
74	↑	Seryl-Tyrosine	Protein digestion & absorption	1.7249	0.786	1.10E−3	2.959
75	↑	Tyrosyl-Glutamine	Alanine, aspartate & glutamate metabolism	2.4162	1.273	1.10E−3	2.957
76	↓	3-Methoxy-4-hydroxyphenylglycolaldehyde	Protein digestion & absorption	0.5381	-0.894	1.18E−3	2.930

Rank	Dir.	Metabolite	Pathway	FC	log₂(FC)	p-value	−log₁₀(p)
77	↑	3,4-Dihydroxystyrene	Amino acid metabolism	2.3987	1.262	1.29E−3	2.890
78	↑	Alanyl-Alanine	Alanine, aspartate & glutamate metabolism	2.4743	1.307	1.31E−3	2.884
79	↑	Isoleucyl-Glutamate	Alanine, aspartate & glutamate metabolism	1.8700	0.903	1.34E−3	2.874
80	↓	Glutaminyl-Proline/Prolyl-Glutamine	Arginine & proline metabolism	0.6210	-0.687	1.39E−3	2.856
81	↓	L-Norleucine	Valine, leucine & isoleucine metabolism	0.4717	-1.084	1.40E−3	2.853
82	↑	Alanyl-Tyrosine	Protein digestion & absorption	15.8070	3.982	1.42E−3	2.848
83	↑	Alanyl-Valine	Valine, leucine & isoleucine metabolism	2.4742	1.307	1.62E−3	2.791
84	↑	3,4-Dihydroxyphenylethyleneglycol 4-O-glucuronide	Amino acid metabolism	2.6419	1.402	1.64E−3	2.784
85	↑	Threoninyl-Glutamine	Alanine, aspartate & glutamate metabolism	2.0900	1.064	1.82E−3	2.741
86	↓	Isomer 1 of Isoleucyl-Alanine	Alanine, aspartate & glutamate metabolism	0.6374	-0.650	1.85E−3	2.733
87	↑	Seryl-Valine	Valine, leucine & isoleucine metabolism	1.5511	0.633	1.95E−3	2.709
88	↑	4'-hydroxypropanolol	Amino acid metabolism	1.6588	0.730	1.99E−3	2.701
89	↓	Glycine	Glycine, serine & threonine metabolism	0.4187	-1.256	2.01E−3	2.697
90	↑	Tyrosyl-Glycine	Glycine, serine & threonine metabolism	1.6838	0.752	2.01E−3	2.696
91	↑	Threoninyl-Valine	Valine, leucine & isoleucine metabolism	2.2260	1.154	2.03E−3	2.692
92	↑	Valyl-Threonine	Glycine, serine & threonine metabolism	1.8230	0.866	2.03E−3	2.692
93	↑	Glutaminyl-Aspartic Acid	Alanine, aspartate & glutamate metabolism	4.0826	2.030	2.08E−3	2.681
94	↓	N-Ethylglycine	Glycine, serine & threonine metabolism	0.5938	-0.752	2.13E−3	2.671
95	↑	Valyl-Leucine	Valine, leucine & isoleucine metabolism	1.6730	0.742	2.48E−3	2.605
96	↑	Lysyl-Glutamine	Alanine, aspartate & glutamate metabolism	3.2433	1.698	2.51E−3	2.600
97	↑	Tyrosyl-Proline	Arginine & proline metabolism	1.9163	0.938	2.59E−3	2.586
98	↓	Homoserine	Glycine, serine & threonine metabolism	0.5970	-0.744	2.67E−3	2.573
99	↓	Threonine	Glycine, serine & threonine metabolism	0.4900	-1.029	3.50E−3	2.456
100	↑	Tyrosyl-Leucine	Valine, leucine & isoleucine metabolism	3.9155	1.969	3.61E−3	2.442
101	↑	4-Hydroxystyrene	Amino acid metabolism	1.8445	0.883	3.69E−3	2.433
102	↓	Allocystathionine	Amino acid metabolism	0.5304	-0.915	3.79E−3	2.422

Rank	Dir.	Metabolite	Pathway	FC	log_2(FC)	p-value	$-log_{10}$(p)
103	↓	3,4-Dihydroxybenzylamine	Amino acid metabolism	0.4737	-1.078	3.86E−3	2.414
104	↑	5-[[(4,7-Dihydroxy-2-Oxo-2H-1-Benzopyran-3-Yl)Amino]Carbonyl]-4-Methyl-1H-Pyrrole-3-Carboxylic Acid	Protein digestion & absorption	2.8553	1.514	3.94E−3	2.404
105	↑	Methionine	Cysteine & methionine metabolism	1.7321	0.792	4.28E−3	2.368
106	↓	(E)-4-Hydroxyphenylacetaldehyde Oxime	Amino acid metabolism	0.4443	-1.170	4.51E−3	2.346
107	↓	Isomer 1 of Ornithine	Arginine & proline metabolism	0.6536	-0.614	4.72E−3	2.326
108	↑	Glutamyl-Histidine	Histidine metabolism	1.6709	0.741	4.79E−3	2.320
109	↑	Valyl-Glutamate	Alanine, aspartate & glutamate metabolism	1.8184	0.863	5.07E−3	2.295
110	↑	Lysyl-Isoleucine	Valine, leucine & isoleucine metabolism	1.8316	0.873	5.22E−3	2.282
111	↑	Isoleucyl-Lysine	Lysine degradation	2.1529	1.106	5.24E−3	2.280
112	↓	Aspartyl-Glycine	Glycine, serine & threonine metabolism	0.6255	-0.677	5.44E−3	2.264
113	↓	Cystathionine	Amino acid metabolism	0.5379	-0.895	5.49E−3	2.261
114	↓	Tyrosinamide	Amino acid metabolism	0.3690	-1.438	5.59E−3	2.253
115	↑	Lysyl-Methionine/Methionyl-Lysine	Cysteine & methionine metabolism	1.7322	0.793	5.73E−3	2.242
116	↑	Glutamyl-Glutamate	Alanine, aspartate & glutamate metabolism	2.3981	1.262	5.80E−3	2.237
117	↓	Formyl-5-Hydroxykynurenamine	Tryptophan metabolism	0.6043	-0.727	5.92E−3	2.228
118	↓	Asparagine	Alanine, aspartate & glutamate metabolism	0.6177	-0.695	5.97E−3	2.224
119	↓	4-Amino-2-hydroxylamino-6-nitrotoluene	Amino acid metabolism	0.6008	-0.735	6.15E−3	2.211
120	↑	Threoninyl-Glycine	Glycine, serine & threonine metabolism	1.6902	0.757	6.24E−3	2.205
121	↑	Lysyl-Asparagine/Asparaginyl-Lysine	Alanine, aspartate & glutamate metabolism	3.3676	1.752	6.43E−3	2.192
122	↑	1,2-Dihydroxynaphthalene-6-sulfonic acid	Amino acid metabolism	1.9684	0.977	6.60E−3	2.180
123	↓	Isomer 1 of 1,2-Dihydroxynaphthalene-6-sulfonic acid	Amino acid metabolism	0.6382	-0.648	7.30E−3	2.137
124	↑	Glutamyl-Alanine	Alanine, aspartate & glutamate metabolism	1.7235	0.785	7.38E−3	2.132
125	↓	Synephrine	Amino acid metabolism	0.4093	-1.289	7.49E−3	2.126
126	↑	Alanyl-Glutamine	Alanine, aspartate & glutamate metabolism	1.8005	0.848	7.84E−3	2.106
127	↑	N2-Acetyl-5'-Hydroxykynurenamine	Amino acid metabolism	2.1548	1.108	8.32E−3	2.080

Rank	Dir.	Metabolite	Pathway	FC	log₂(FC)	p-value	−log₁₀(p)
128	↑	Lysyl-Leucine	Valine, leucine & isoleucine metabolism	1.7015	0.767	8.39E−3	2.076
129	↑	Threoninyl-Aspartate	Protein digestion & absorption	1.7148	0.778	8.88E−3	2.052
130	↑	Isoleucyl-Glycine	Glycine, serine & threonine metabolism	1.7532	0.810	9.40E−3	2.027
131	↑	Tyrosine	Amino acid metabolism	1.9259	0.946	9.61E−3	2.017
132	↓	Salsolinol 1-carboxylic acid	Tyrosine metabolism	0.5985	-0.741	1.02E−2	1.993
133	↑	Histidinyl-Serine	Glycine, serine & threonine metabolism	1.8239	0.867	1.02E−2	1.991
134	↑	3-Amino-3-(4-hydroxyphenyl)propanoic acid	Amino acid metabolism	1.9185	0.940	1.03E−2	1.986
135	↑	2-Hydroxyhepta-2,4-dienedioic acid	Amino acid metabolism	1.6100	0.687	1.04E−2	1.983
136	↑	Seryl-Alanine	Alanine, aspartate & glutamate metabolism	1.5277	0.611	1.07E−2	1.970
137	↑	2-Aminoadipate 6-Semialdehyde	Amino acid metabolism	1.7095	0.774	1.16E−2	1.936
138	↑	Adrenochrome o-semiquinone	Tyrosine metabolism	1.7091	0.773	1.16E−2	1.934
139	↑	Aspartyl-Lysine	Lysine degradation	1.5111	0.596	1.18E−2	1.929
140	↑	Leucyl-Arginine	Protein digestion & absorption	1.5282	0.612	1.21E−2	1.916
141	↑	Hydroxy-lacosamide	Amino acid metabolism	1.6017	0.680	1.23E−2	1.911
142	↑	Allysine	Lysine degradation	1.6876	0.755	1.26E−2	1.901
143	↑	Hydroquinone	Amino acid metabolism	1.7528	0.810	1.33E−2	1.876
144	↑	Tryptophyl-Glycine	Glycine, serine & threonine metabolism	1.5771	0.657	1.35E−2	1.870
145	↑	Asparaginyl-Lysine	Lysine degradation	2.2003	1.138	1.36E−2	1.865
146	↓	p-Hydroxyphenylacetylglycine	Glycine, serine & threonine metabolism	0.5357	-0.900	1.46E−2	1.835
147	↑	N-Acetyl-Formyl-5-hydroxykynurenamine	Tryptophan metabolism	1.8903	0.919	1.53E−2	1.816
148	↑	Arginyl-Isoleucine	Valine, leucine & isoleucine metabolism	1.8294	0.871	1.55E−2	1.809
149	↓	L-2-Amino-3-(4-aminophenyl)propanoic acid	Amino acid metabolism	0.6193	-0.691	1.57E−2	1.803
150	↑	N8-Acetylspermidine	Amino acid metabolism	2.1265	1.088	1.62E−2	1.790
151	↑	Lysyl-Serine	Glycine, serine & threonine metabolism	1.5759	0.656	1.74E−2	1.760
152	↑	Leucine	Valine, leucine & isoleucine metabolism	1.7382	0.798	1.76E−2	1.756
153	↑	Histidinyl-Glutamate	Alanine, aspartate & glutamate metabolism	1.7001	0.766	1.77E−2	1.752

Rank	Dir.	Metabolite	Pathway	FC	$\log_2$(FC)	p-value	$-\log_{10}$(p)
154	↓	Kynurenine	Tryptophan metabolism	0.5872	-0.768	1.79E−2	1.747
155	↑	2(N)-Methyl-norsalsolinol	Tyrosine metabolism	1.8369	0.877	1.80E−2	1.744
156	↑	Lysyl-Glutamate	Alanine, aspartate & glutamate metabolism	1.5191	0.603	1.97E−2	1.706
157	↓	Isomer 1 of 3-Amino-2-piperidone	Amino acid metabolism	0.5767	-0.794	2.03E−2	1.692
158	↑	Chlorohydroquinone	Amino acid metabolism	2.1910	1.132	2.08E−2	1.683
159	↑	Seryl-Methionine	Cysteine & methionine metabolism	1.9415	0.957	2.24E−2	1.649
160	↓	3-Hydroxykynurenamine	Amino acid metabolism	0.6467	-0.629	2.29E−2	1.641
161	↑	Leucyl-Tryptophan	Tryptophan metabolism	1.6990	0.765	2.30E−2	1.638
162	↑	Glutaminyl-Serine/Seryl-Glutamine	Alanine, aspartate & glutamate metabolism	2.2900	1.195	2.33E−2	1.633
163	↑	Lysyl-Histidine	Histidine metabolism	3.0774	1.622	2.38E−2	1.623
164	↑	Phloroglucinol	Amino acid metabolism	1.7151	0.778	2.41E−2	1.618
165	↑	4-Hydroxyphenylglyoxylic Acid	Amino acid metabolism	1.6367	0.711	2.47E−2	1.607
166	↓	L-Homophenylalanine	Phenylalanine metabolism	0.5291	-0.918	2.49E−2	1.604
167	↑	Isomer 1 of Lysyl-Proline	Arginine & proline metabolism	1.5189	0.603	2.55E−2	1.593
168	↑	2'-Aminobiphenyl-2,3-diol	Amino acid metabolism	1.8136	0.859	2.58E−2	1.589
169	↑	Bis(Glutathionyl)Spermine	Protein digestion & absorption	1.7203	0.783	2.67E−2	1.573
170	↑	Glutaminyl-Glutamate	Alanine, aspartate & glutamate metabolism	1.8426	0.882	2.71E−2	1.567
171	↑	Methionine Sulfoxide	Cysteine & methionine metabolism	1.7977	0.846	2.80E−2	1.552
172	↑	Isomer 1 of Seryl-Tyrosine	Protein digestion & absorption	1.5300	0.614	2.90E−2	1.537
173	↑	Tyrosyl-Valine	Valine, leucine & isoleucine metabolism	1.6582	0.730	2.94E−2	1.531
174	↓	Isomer 2 of 5-Aminopentanoic acid	Amino acid metabolism	0.6134	-0.705	2.96E−2	1.529
175	↑	p-Coumaroylagmatine	Amino acid metabolism	1.8173	0.862	2.99E−2	1.524
176	↑	Valyl-Asparagine	Alanine, aspartate & glutamate metabolism	1.5857	0.665	3.14E−2	1.503
177	↑	Asparaginyl-Threonine	Glycine, serine & threonine metabolism	1.6537	0.726	3.30E−2	1.481
178	↑	Glycyl-Threonine	Glycine, serine & threonine metabolism	1.6475	0.720	3.33E−2	1.478
179	↓	Serine	Glycine, serine & threonine metabolism	0.6562	-0.608	3.34E−2	1.476

Rank	Dir.	Metabolite	Pathway	FC	$\log_2$(FC)	p-value	$-\log_{10}$(p)
180	↓	L-Targinine	Amino acid metabolism	0.6223	-0.684	3.37E−2	1.472
181	↓	5-Hydroxy-N-Formylkynurenine	Tryptophan metabolism	0.5089	-0.974	3.38E−2	1.471
182	↓	Spermine dialdehyde	Amino acid metabolism	0.6107	-0.712	3.44E−2	1.463
183	↓	2,8-Dihydroxyadenine	Amino acid metabolism	0.5334	-0.907	3.68E−2	1.434
184	↓	Isomer 1 of 4-Aminobutyraldehyde	Amino acid metabolism	0.4153	-1.268	4.31E−2	1.365
185	↑	Prolyl-Proline	Arginine & proline metabolism	1.6638	0.734	4.84E−2	1.315
186	↓	3-Amino-2-Piperidone	Amino acid metabolism	0.6035	-0.729	4.92E−2	1.308

Significance criteria: $p < 0.05$ (Welch's two-tailed t-test) AND |FC| > 1.5. Metabolites ranked by ascending p-value. FC = fold change of first group relative to second. ↑ = increased; ↓ = decreased. $\log_2$(FC) and $-\log_{10}$(p) provided for volcano plot reference. Pathway annotations based on KEGG Bos taurus metabolic library. Source data: metaboan2.csv (n = 10 per group; 474 metabolites after Tier 3 exclusions).

Table S5. All significantly altered named metabolites in SCM-D21 vs H-D21 (n = 36), ranked by ascending p-value.

Rank	Dir.	Metabolite	Pathway	FC	$\log_2$(FC)	p-value	$-\log_{10}$(p)
1	↑	3-Cyano-Alanine	Cyanoamino acid metabolism	1.5962	0.675	**4.34E−5**	4.363
2	↑	Leucyl-Proline	Arginine & proline metabolism	1.9289	0.948	2.64E−3	2.578
3	↑	5-Aminopentanamide	Amino acid metabolism	1.5998	0.678	2.79E−3	2.554
4	↑	Prolyl-Valine	Valine, leucine & isoleucine metabolism	1.8380	0.878	2.86E−3	2.543
5	↓	Cytidine	Pyrimidine metabolism	0.5513	-0.859	2.98E−3	2.526
6	↑	Alanyl-Proline	Arginine & proline metabolism	1.7974	0.846	4.06E−3	2.392
7	↑	Isomer 2 of 3-Hydroxymelatonin	Amino acid metabolism	1.7519	0.809	4.48E−3	2.348
8	↑	Salsolinol 1-carboxylic acid	Tyrosine metabolism	1.8728	0.905	4.54E−3	2.343
9	↓	4-O-Methylgallic acid	Protein digestion & absorption	0.5448	-0.876	6.33E−3	2.199
10	↑	N-Acetyl-N-Methylserotonin	Tryptophan metabolism	1.6048	0.682	6.92E−3	2.160
11	↑	Phenol	Amino acid metabolism	1.8592	0.895	6.96E−3	2.157
12	↑	Phenylalanyl-Alanine	Alanine, aspartate & glutamate metabolism	1.8181	0.862	8.03E−3	2.096
13	↓	4-Hydroxy-4-methylglutamic acid	Amino acid metabolism	0.4271	-1.227	8.06E−3	2.094
14	↓	4-Chloro-L-lysine	Lysine degradation	0.5993	-0.739	9.39E−3	2.027
15	↑	Norsalsolinol	Tyrosine metabolism	2.0831	1.059	9.55E−3	2.020
16	↑	Isomer 1 of 3-Hydroxymelatonin	Amino acid metabolism	1.6387	0.713	1.14E−2	1.942
17	↓	Isomer 1 of gamma-Amino-gamma-cyanobutanoic acid	Amino acid metabolism	0.5784	-0.790	1.20E−2	1.919
18	↑	Isoleucylproline	Arginine & proline metabolism	1.6359	0.710	1.28E−2	1.893
19	↑	Prolyl-Lysine	Lysine degradation	1.6039	0.682	1.29E−2	1.888
20	↑	Glutamylproline	Arginine & proline metabolism	1.6894	0.757	1.33E−2	1.876
21	↑	5-Methoxytryptophan	Tryptophan metabolism	2.7018	1.434	1.52E−2	1.818
22	↓	3-O-Methylgallic Acid	Protein digestion & absorption	0.5385	-0.893	1.55E−2	1.810
23	↑	Prolyl-Proline	Arginine & proline metabolism	2.7194	1.443	1.70E−2	1.770
24	↑	Alanyl-Phenylalanine	Phenylalanine metabolism	1.6557	0.727	1.93E−2	1.714

Rank	Dir.	Metabolite	Pathway	FC	$\log_2$(FC)	p-value	$-\log_{10}$(p)
25	↑	4-Hydroxybenzaldehyde/3-Hydroxybenzaldehyde	Tyrosine metabolism	2.4949	1.319	1.96E−2	1.709
26	↓	3,4-Dihydroxyphenylethyleneglycol 4-O-glucuronide	Amino acid metabolism	0.3347	-1.579	2.12E−2	1.673
27	↑	Tyrosyl-Proline	Arginine & proline metabolism	1.5322	0.616	2.14E−2	1.670
28	↑	Proline	Arginine & proline metabolism	1.6987	0.764	2.30E−2	1.639
29	↑	N-Acetyl-Dihydrofolic Acid	Amino acid metabolism	1.6839	0.752	2.42E−2	1.617
30	↓	Lysyl-Phenylalanine	Phenylalanine metabolism	0.6615	-0.596	3.14E−2	1.503
31	↑	3-Amino-2-Piperidone	Amino acid metabolism	1.5455	0.628	3.16E−2	1.500
32	↓	Uracil	Pyrimidine metabolism	0.3859	-1.374	3.65E−2	1.437
33	↑	Lysyl-Proline	Arginine & proline metabolism	1.5876	0.667	3.81E−2	1.419
34	↓	Isomer 1 of 4-Chloro-L-lysine	Lysine degradation	0.6584	-0.603	3.96E−2	1.402
35	↑	4-Hydroxybenzoic acid	Amino acid metabolism	1.9326	0.951	4.74E−2	1.324
36	↑	Aspartyl-Proline	Arginine & proline metabolism	1.8218	0.865	4.75E−2	1.324

Significance criteria: $p < 0.05$ (Welch's two-tailed t-test) AND |FC| > 1.5. Metabolites ranked by ascending p-value. FC = fold change of first group relative to second. ↑ = increased; ↓ = decreased. $\log_2$(FC) and $-\log_{10}$(p) provided for volcano plot reference. Pathway annotations based on KEGG Bos taurus metabolic library. Source data: metaboan2.csv (n = 10 per group; 474 metabolites after Tier 3 exclusions).

Table S6. Longitudinal changes in SCM quarters: SCM-D21 vs SCM-D2 (n = 316 significantly altered metabolites), ranked by ascending p-value.

Rank	Dir.	Metabolite	Pathway	FC	$\log_2$(FC)	p-value	$-\log_{10}$(p)
1	↑	Aspartic acid	Alanine, aspartate & glutamate metabolism	6.1271	2.615	**2.50E−12**	11.603
2	↓	Alanyl-Methionine	Cysteine & methionine metabolism	0.0848	-3.559	**4.05E−12**	11.392
3	↓	Phenylalanyl-Glycine	Glycine, serine & threonine metabolism	0.2496	-2.002	**2.06E−11**	10.685
4	↑	N-Acetylindoxyl	Tryptophan metabolism	10.4530	3.386	**1.28E−10**	9.893
5	↑	Alanine	Alanine, aspartate & glutamate metabolism	6.3707	2.671	**1.81E−10**	9.743
6	↑	1-Methylguanosine	Purine metabolism	3.5266	1.818	**2.99E−10**	9.525
7	↓	Arginyl-Leucine	Valine, leucine & isoleucine metabolism	0.1238	-3.013	**3.48E−10**	9.458
8	↑	Cis-4-Hydroxy-D-Proline	Arginine & proline metabolism	4.6854	2.228	**5.48E−10**	9.261
9	↓	Tyrosyl-Valine	Valine, leucine & isoleucine metabolism	0.1945	-2.362	**5.96E−10**	9.225
10	↑	Glycine	Glycine, serine & threonine metabolism	8.0000	3.000	**7.27E−10**	9.138
11	↑	Beta-Ethynylserine	Glycine, serine & threonine metabolism	5.2615	2.396	**9.02E−10**	9.045
12	↑	gamma-Glutamyl-Proline	Arginine & proline metabolism	3.5720	1.837	**1.01E−9**	8.995
13	↑	Cystine	Cysteine & methionine metabolism	7.5388	2.914	**1.08E−9**	8.966
14	↑	4-Oxoproline	Arginine & proline metabolism	5.7415	2.521	**1.75E−9**	8.756
15	↑	Isomer 1 of Sarcosine	Glycine, serine & threonine metabolism	13.8840	3.795	**1.89E−9**	8.724
16	↑	Isomer 1 of Iminodiacetic acid	Amino acid metabolism	2.9219	1.547	**2.05E−9**	8.687
17	↓	7,8-Dihydroxanthopterin	Amino acid metabolism	0.1781	-2.489	**2.25E−9**	8.648
18	↑	Histidine	Histidine metabolism	6.9668	2.800	**2.36E−9**	8.626
19	↓	Leucyl-Leucine	Valine, leucine & isoleucine metabolism	0.2677	-1.901	**2.79E−9**	8.554
20	↑	Lysyl-Aspartate	Protein digestion & absorption	3.9939	1.998	**3.30E−9**	8.482
21	↓	Tyrosyl-Leucine	Valine, leucine & isoleucine metabolism	0.0232	-5.429	**4.14E−9**	8.383
22	↑	4-Amino-2-hydroxylamino-6-nitrotoluene	Amino acid metabolism	12.0590	3.592	**7.17E−9**	8.145
23	↑	Asparaginyl-Serine	Glycine, serine & threonine metabolism	3.1560	1.658	**1.01E−8**	7.996
24	↑	4-Aminocatechol	Amino acid metabolism	5.6065	2.487	**1.12E−8**	7.949

Rank	Dir.	Metabolite	Pathway	FC	log_2(FC)	p-value	$-log_{10}$(p)
25	↑	4-Hydroxystyrene	Amino acid metabolism	4.3959	2.136	**1.31E−8**	7.881
26	↑	Dehydroalanine	Alanine, aspartate & glutamate metabolism	4.6111	2.205	**1.39E−8**	7.857
27	↑	Valyl-Alanine	Alanine, aspartate & glutamate metabolism	3.8466	1.944	**1.69E−8**	7.772
28	↓	Pyrrolidine	Amino acid metabolism	0.1951	-2.358	**2.33E−8**	7.633
29	↑	1-Aminocyclopropane-1-Carboxylic Acid	Amino acid metabolism	3.8613	1.949	**2.40E−8**	7.620
30	↓	Diethanolamine	Glycerophospholipid metabolism	0.1875	-2.415	**3.25E−8**	7.488
31	↓	Choline	Glycerophospholipid metabolism	0.1698	-2.558	**3.56E−8**	7.449
32	↑	Phenylalanine	Phenylalanine metabolism	3.8499	1.945	**3.60E−8**	7.444
33	↑	Salicylamide	Amino acid metabolism	4.4178	2.143	**4.25E−8**	7.372
34	↓	N-Acetyl-3-Hydroxyanthranilic acid	Tryptophan metabolism	0.1854	-2.432	**4.55E−8**	7.342
35	↑	p-Aminobenzoic acid	Amino acid metabolism	4.4761	2.162	**4.88E−8**	7.311
36	↑	7-Cyano-7-Carbaguanine	Amino acid metabolism	8.9761	3.166	**5.04E−8**	7.298
37	↓	Coutaric acid	Amino acid metabolism	0.3074	-1.702	**5.49E−8**	7.260
38	↑	Octopamine	Tyrosine metabolism	3.9329	1.976	**6.23E−8**	7.206
39	↓	Isomer 1 of 4-Chloro-L-lysine	Lysine degradation	0.1340	-2.900	**6.65E−8**	7.177
40	↓	Pantothenic Acid	Pantothenate & CoA biosynthesis	0.1289	-2.956	**7.48E−8**	7.126
41	↑	3-Aminoisobutanoic acid	Amino acid metabolism	5.8964	2.560	**7.72E−8**	7.112
42	↑	Proline	Arginine & proline metabolism	5.1971	2.378	**8.18E−8**	7.087
43	↑	Tryptophan	Tryptophan metabolism	4.0458	2.016	**8.76E−8**	7.057
44	↑	2-Aminophenol	Amino acid metabolism	2.6533	1.408	**1.14E−7**	6.942
45	↑	N-Methyl aspartate	Protein digestion & absorption	2.4265	1.279	**1.33E−7**	6.878
46	↑	L-Isoleucine	Valine, leucine & isoleucine metabolism	3.9483	1.981	**1.36E−7**	6.865
47	↑	Formyl-5-Hydroxykynurenamine	Tryptophan metabolism	7.9843	2.997	**1.59E−7**	6.800
48	↑	Glutamic acid	Amino acid metabolism	3.2862	1.716	**1.67E−7**	6.777
49	↑	N(6)-Methyllysine	Lysine degradation	4.9361	2.303	**1.95E−7**	6.710
50	↑	N-Acetyl-Dihydrofolic Acid	Amino acid metabolism	3.5934	1.845	**2.17E−7**	6.663

Rank	Dir.	Metabolite	Pathway	FC	$\log_2$(FC)	p-value	$-\log_{10}$(p)
51	↓	Pyridoxal	Vitamin B6 metabolism	0.2327	-2.103	**2.32E−7**	6.634
52	↓	Leucyl-Asparagine	Alanine, aspartate & glutamate metabolism	0.3512	-1.510	**2.41E−7**	6.618
53	↑	3-Hydroxymelatonin	Amino acid metabolism	4.5834	2.196	**2.95E−7**	6.531
54	↑	Alanyl-Phenylalanine	Phenylalanine metabolism	5.0983	2.350	**3.43E−7**	6.465
55	↑	Leucine	Valine, leucine & isoleucine metabolism	3.7046	1.889	**3.44E−7**	6.463
56	↓	Histidinyl-Lysine	Lysine degradation	0.2292	-2.125	**3.45E−7**	6.462
57	↓	Amoxicillin	Amino acid metabolism	0.2137	-2.226	**3.49E−7**	6.458
58	↑	(E)-6'-Hydroxyferulate	Amino acid metabolism	3.4946	1.805	**3.49E−7**	6.458
59	↓	Tyrosyl-Glutamine	Alanine, aspartate & glutamate metabolism	0.2282	-2.132	**4.24E−7**	6.372
60	↓	4-Hydroxyphenylglyoxylic Acid	Amino acid metabolism	0.1502	-2.736	**5.01E−7**	6.300
61	↑	Valyl-Glutamate	Alanine, aspartate & glutamate metabolism	3.9865	1.995	**5.99E−7**	6.222
62	↓	Leucocyanidin	Amino acid metabolism	0.1964	-2.348	**6.84E−7**	6.165
63	↓	Threoninyl-Valine	Valine, leucine & isoleucine metabolism	0.2554	-1.969	**7.21E−7**	6.142
64	↓	Isoglutamine	Alanine, aspartate & glutamate metabolism	0.1769	-2.499	**7.40E−7**	6.131
65	↑	(2R,4S)-2,4-Diaminopentanoic Acid	Amino acid metabolism	7.4166	2.891	**7.90E−7**	6.102
66	↑	4-Guanidinobutanal	Amino acid metabolism	4.2953	2.103	**8.06E−7**	6.094
67	↑	Isomer 2 of Prolyl-Alanine	Alanine, aspartate & glutamate metabolism	3.0509	1.609	**8.68E−7**	6.061
68	↑	Tyrosyl-Glycine	Glycine, serine & threonine metabolism	3.0653	1.616	**9.80E−7**	6.009
69	↑	3,4-Dihydroxystyrene	Amino acid metabolism	3.9805	1.993	**1.04E−6**	5.983
70	↑	2-Hydroxy-4-imino-2,5-cyclohexadienone	Amino acid metabolism	2.0518	1.037	**1.37E−6**	5.862
71	↑	Methionine	Cysteine & methionine metabolism	2.7789	1.474	**1.51E−6**	5.820
72	↑	Aspartyl-Valine	Valine, leucine & isoleucine metabolism	3.9411	1.979	**1.58E−6**	5.802
73	↓	Ethanolamine	Glycerophospholipid metabolism	0.1636	-2.612	**1.74E−6**	5.759
74	↑	Ornithine	Arginine & proline metabolism	6.7100	2.746	**1.81E−6**	5.742
75	↑	6-Lactoyl-5,6,7,8-Tetrahydropterin	Amino acid metabolism	2.2040	1.140	**1.91E−6**	5.718
76	↑	2-Hydroxyhepta-2,4-dienedioic acid	Amino acid metabolism	2.5608	1.357	**2.04E−6**	5.689

Rank	Dir.	Metabolite	Pathway	FC	$\log_2$(FC)	p-value	$-\log_{10}$(p)
77	↓	S-Cysteinosuccinic acid	Protein digestion & absorption	0.3199	-1.644	**2.42E−6**	5.616
78	↑	Seryl-Tyrosine	Protein digestion & absorption	3.5050	1.809	**2.72E−6**	5.565
79	↓	Histidinyl-Isoleucine	Valine, leucine & isoleucine metabolism	0.3706	-1.432	**2.82E−6**	5.550
80	↓	Isomer 2 of 4-Chloro-L-lysine	Lysine degradation	0.2556	-1.968	**2.88E−6**	5.541
81	↑	Tyrosyl-Proline	Arginine & proline metabolism	2.9666	1.569	**2.93E−6**	5.533
82	↑	4-Aminobutyraldehyde	Amino acid metabolism	2.5856	1.370	**2.93E−6**	5.532
83	↓	2-Aminoadipate 6-Semialdehyde	Amino acid metabolism	0.3480	-1.523	**2.97E−6**	5.528
84	↓	Allysine	Lysine degradation	0.3525	-1.504	**2.98E−6**	5.526
85	↑	Adrenochrome o-semiquinone	Tyrosine metabolism	4.2011	2.071	**3.05E−6**	5.515
86	↓	Lysyl-Valine	Valine, leucine & isoleucine metabolism	0.2630	-1.927	**3.08E−6**	5.511
87	↓	Atenolol	Amino acid metabolism	0.3660	-1.450	**3.36E−6**	5.473
88	↑	Seryl-Alanine	Alanine, aspartate & glutamate metabolism	3.2725	1.710	**3.52E−6**	5.454
89	↑	Glutamyl-Aspartate	Protein digestion & absorption	2.7426	1.456	**3.53E−6**	5.452
90	↑	Prolyl-Alanine	Alanine, aspartate & glutamate metabolism	4.3190	2.111	**3.67E−6**	5.435
91	↓	Valyl-Lysine	Lysine degradation	0.2622	-1.931	**3.76E−6**	5.425
92	↑	Norsalsolinol	Tyrosine metabolism	4.2556	2.089	**4.08E−6**	5.390
93	↓	Nocardicin G	Amino acid metabolism	0.2196	-2.187	**4.10E−6**	5.387
94	↓	alpha-D-Glucosamine 1-phosphate	Protein digestion & absorption	0.2974	-1.750	**4.16E−6**	5.381
95	↓	Carbapenem biosynthesis intermediate 3	Amino acid metabolism	0.2525	-1.986	**4.84E−6**	5.315
96	↓	1,2,3-Trihydroxybenzene	Amino acid metabolism	0.5489	-0.865	**4.88E−6**	5.312
97	↓	2-Hydroxymuconate Semialdehyde	Amino acid metabolism	0.1614	-2.631	**5.52E−6**	5.258
98	↓	Mangiferin	Flavonoid biosynthesis	0.2476	-2.014	**5.59E−6**	5.253
99	↑	p-Hydroxyphenylacetylglycine	Glycine, serine & threonine metabolism	3.3634	1.750	**5.81E−6**	5.236
100	↑	Tyrosine	Amino acid metabolism	3.5443	1.826	**5.85E−6**	5.233
101	↓	3,4-Dihydroxyphenylethyleneglycol 3-O-glucuronide	Amino acid metabolism	0.2700	-1.889	**5.97E−6**	5.224
102	↑	Tryptophyl-Aspartate	Protein digestion & absorption	2.6519	1.407	**6.11E−6**	5.214

Rank	Dir.	Metabolite	Pathway	FC	$\log_2$(FC)	p-value	$-\log_{10}$(p)
103	↑	3-Amino-3-(4-hydroxyphenyl)propanoic acid	Amino acid metabolism	3.5736	1.837	**6.28E−6**	5.202
104	↓	Aspartyl-Methionine	Cysteine & methionine metabolism	0.4882	-1.034	**6.76E−6**	5.170
105	↓	2,3-Dihydroxycarbamazepine	Amino acid metabolism	0.4804	-1.058	**6.92E−6**	5.160
106	↑	N-Acetyl-L-Tyrosine	Protein digestion & absorption	4.0774	2.028	**7.34E−6**	5.134
107	↑	Lysyl-Serine	Glycine, serine & threonine metabolism	3.0682	1.617	**8.10E−6**	5.092
108	↑	2,6-Dihydroxypseudooxynicotine	Amino acid metabolism	2.2452	1.167	**8.96E−6**	5.048
109	↓	Isomer 1 of Lysyl-Proline	Arginine & proline metabolism	0.3269	-1.613	**8.96E−6**	5.048
110	↑	2,6-Diamino-4-hydroxy-5-N-methylformamidopyrimidine	Amino acid metabolism	3.1346	1.648	**9.19E−6**	5.037
111	↑	2-Amino-2-methyl-1,3-propanediol	Amino acid metabolism	2.2609	1.177	**9.49E−6**	5.022
112	↓	Phloroglucinol	Amino acid metabolism	0.1722	-2.537	**1.08E−5**	4.966
113	↑	Homoserine	Glycine, serine & threonine metabolism	1.6277	0.703	**1.14E−5**	4.943
114	↓	Leucyl-Glycine	Glycine, serine & threonine metabolism	0.4372	-1.194	**1.17E−5**	4.933
115	↑	4-Aminohippuric acid	Purine metabolism	2.2017	1.139	**1.20E−5**	4.920
116	↓	Histidinyl-Glutamine	Alanine, aspartate & glutamate metabolism	0.2428	-2.042	**1.36E−5**	4.867
117	↑	Citrulline	Arginine & proline metabolism	7.1511	2.838	**1.41E−5**	4.850
118	↓	Isomer 1 of p-Hydroxyphenylacetylglycine	Glycine, serine & threonine metabolism	0.4606	-1.118	**1.54E−5**	4.812
119	↑	Neopterin	Amino acid metabolism	1.7446	0.803	**1.57E−5**	4.805
120	↑	Uridine	Pyrimidine metabolism	2.5502	1.351	**1.60E−5**	4.795
121	↑	Beta-Alanine	Alanine, aspartate & glutamate metabolism	3.1764	1.667	**1.72E−5**	4.764
122	↑	Isoleucyl-Glycine	Glycine, serine & threonine metabolism	3.0840	1.625	**1.82E−5**	4.741
123	↑	N-gamma-Glutamyl-S-allylcysteine	Protein digestion & absorption	3.2805	1.714	**1.85E−5**	4.733
124	↑	Echinatin	Amino acid metabolism	3.9784	1.992	**2.11E−5**	4.676
125	↑	Seryl-Cysteine	Protein digestion & absorption	2.6708	1.417	**2.14E−5**	4.670
126	↑	Iminodiacetic acid	Amino acid metabolism	2.6772	1.421	**2.19E−5**	4.660
127	↑	Methionine Sulfoxide	Cysteine & methionine metabolism	2.8439	1.508	**2.20E−5**	4.657
128	↑	4-Methylene-Glutamine	Alanine, aspartate & glutamate metabolism	6.6845	2.741	**2.22E−5**	4.653

Rank	Dir.	Metabolite	Pathway	FC	$\log_2$(FC)	p-value	$-\log_{10}$(p)
129	↑	Alanyl-Alanine	Alanine, aspartate & glutamate metabolism	3.0439	1.606	**2.28E−5**	4.642
130	↓	2'-Aminobiphenyl-2,3-diol	Amino acid metabolism	0.2496	-2.002	**2.30E−5**	4.638
131	↑	Isomer 1 of Arginine	Amino acid metabolism	2.0914	1.064	**2.67E−5**	4.574
132	↑	3-O-Methylgallic Acid	Protein digestion & absorption	3.1766	1.668	**2.78E−5**	4.556
133	↓	S-Adenosyl-L-homocysteine	Cysteine & methionine metabolism	0.1931	-2.372	**2.84E−5**	4.547
134	↑	Symmetric dimethylarginine	Amino acid metabolism	2.6473	1.404	**2.90E−5**	4.538
135	↑	Seryl-Valine	Valine, leucine & isoleucine metabolism	2.5573	1.355	**2.94E−5**	4.532
136	↑	Aspartyl-Aspartate	Protein digestion & absorption	3.0480	1.608	**2.99E−5**	4.524
137	↑	Isomer 1 of Benzocaine	Amino acid metabolism	3.3974	1.764	**3.13E−5**	4.504
138	↑	Serylglutamic Acid	Protein digestion & absorption	4.8660	2.283	**3.46E−5**	4.460
139	↑	2'-Hydroxyacetophenone	Amino acid metabolism	2.4861	1.314	**3.47E−5**	4.460
140	↓	L-2-Amino-3-(4-aminophenyl)propanoic acid	Amino acid metabolism	0.3310	-1.595	**4.16E−5**	4.381
141	↓	Isomer 2 of 5-Aminopentanoic acid	Amino acid metabolism	0.4096	-1.288	**4.17E−5**	4.380
142	↑	5-Hydroxyectoine	Amino acid metabolism	2.7336	1.451	**4.32E−5**	4.365
143	↓	3,4-Dihydroxyphenylethyleneglycol 4-O-glucuronide	Amino acid metabolism	0.1296	-2.948	**4.36E−5**	4.361
144	↑	Prolyl-Lysine	Lysine degradation	2.5612	1.357	**4.54E−5**	4.343
145	↑	Threo-3-Methylaspartic Acid	Alanine, aspartate & glutamate metabolism	2.1181	1.083	**4.83E−5**	4.316
146	↓	Galactosylhydroxylysine	Lysine degradation	0.2682	-1.898	**5.19E−5**	4.285
147	↓	2-Hydroxy-6-oxonona-2,4-diene-1,9-dioic acid	Amino acid metabolism	0.4268	-1.228	**5.40E−5**	4.268
148	↑	Alanyl-Glutamine	Alanine, aspartate & glutamate metabolism	2.3452	1.230	**5.59E−5**	4.253
149	↑	2-Hydroxy-2,4-Pentadienoic Acid	Amino acid metabolism	2.4045	1.266	**6.03E−5**	4.220
150	↑	Hypotaurine	Taurine & hypotaurine metabolism	5.4879	2.456	**6.43E−5**	4.192
151	↑	3,4-Dihydroxybenzeneacetic acid	Amino acid metabolism	1.7032	0.768	**6.80E−5**	4.168
152	↑	Butylated hydroxytoluene	Amino acid metabolism	1.7147	0.778	**6.83E−5**	4.165
153	↑	N2'-Acetyl-3'-Hydroxy-L-kynurenine	Tryptophan metabolism	2.0815	1.058	**7.02E−5**	4.154
154	↓	Lysyl-Leucine	Valine, leucine & isoleucine metabolism	0.3992	-1.325	**7.11E−5**	4.148

Rank	Dir.	Metabolite	Pathway	FC	$\log_2$(FC)	p-value	$-\log_{10}$(p)
155	↑	D-histidine methyl ester	Histidine metabolism	3.3415	1.740	**7.57E−5**	4.121
156	↓	3-Hydroxykynurenamine	Amino acid metabolism	0.2687	-1.896	**7.71E−5**	4.113
157	↓	Benzene-1,2,4-Triol	Amino acid metabolism	0.3179	-1.654	**7.85E−5**	4.105
158	↑	Seryl-Threonine	Glycine, serine & threonine metabolism	1.8875	0.917	**8.32E−5**	4.080
159	↓	7-Methylguanine	Protein digestion & absorption	0.5312	-0.913	**8.32E−5**	4.080
160	↓	Lysine	Lysine degradation	0.6385	-0.647	**8.46E−5**	4.073
161	↓	Seryl-Methionine	Cysteine & methionine metabolism	0.3083	-1.698	**8.79E−5**	4.056
162	↑	Kynurenine	Tryptophan metabolism	2.4563	1.296	**9.16E−5**	4.038
163	↓	2(N)-Methyl-norsalsolinol	Tyrosine metabolism	0.2876	-1.798	**9.22E−5**	4.035
164	↑	5-Hydroxykynurenine	Tryptophan metabolism	3.5768	1.839	**9.58E−5**	4.019
165	↑	Leucyl-Aspartate	Protein digestion & absorption	2.6568	1.410	**1.01E−4**	3.998
166	↓	Leucyl-Histidine	Histidine metabolism	0.3608	-1.470	**1.01E−4**	3.996
167	↑	p-Hydroxyphenylacetic acid	Amino acid metabolism	1.5113	0.596	**1.01E−4**	3.996
168	↑	2,6-Dihydroxybenzoic acid	Amino acid metabolism	1.8063	0.853	**1.01E−4**	3.994
169	↑	Histidinyl-Glutamate	Alanine, aspartate & glutamate metabolism	3.5876	1.843	**1.13E−4**	3.948
170	↑	1H-Indole-3-methanamine	Amino acid metabolism	1.8922	0.920	**1.13E−4**	3.948
171	↑	Glycyl-Asparagine	Alanine, aspartate & glutamate metabolism	2.1040	1.073	**1.15E−4**	3.940
172	↑	4-O-Methylgallic acid	Protein digestion & absorption	2.3545	1.236	**1.15E−4**	3.940
173	↓	Glutaminyl-Histidine	Histidine metabolism	0.3243	-1.625	**1.18E−4**	3.929
174	↓	Leucyl-Tryptophan	Tryptophan metabolism	0.4531	-1.142	**1.20E−4**	3.920
175	↑	Aspartyl-Lysine	Lysine degradation	1.8576	0.893	**1.29E−4**	3.890
176	↑	Isomer 1 of 1,2-Dihydroxynaphthalene-6-sulfonic acid	Amino acid metabolism	1.5549	0.637	**1.34E−4**	3.872
177	↓	1,4-Dihydroxy-6-naphthoic acid	Amino acid metabolism	0.4180	-1.258	**1.38E−4**	3.860
178	↑	Methylcysteine	Cysteine & methionine metabolism	5.3687	2.425	**1.51E−4**	3.820
179	↓	Pyridoxamine	Amino acid metabolism	0.6587	-0.602	**1.53E−4**	3.814
180	↑	Homocitrulline	Arginine & proline metabolism	2.3164	1.212	**1.98E−4**	3.704

Rank	Dir.	Metabolite	Pathway	FC	$\log_2$(FC)	p-value	$-\log_{10}$(p)
181	↑	Prolyl-Proline	Arginine & proline metabolism	5.7339	2.520	**2.00E−4**	3.699
182	↑	Isomer 2 of 3-Hydroxymelatonin	Amino acid metabolism	1.9635	0.973	**2.12E−4**	3.673
183	↑	7,8-Dihydroxykynurenic acid	Tryptophan metabolism	2.7941	1.482	**2.18E−4**	3.662
184	↑	Glutamyl-Histidine	Histidine metabolism	2.9423	1.557	**2.28E−4**	3.642
185	↑	(R)-1-Aminopropan-2-Ol	Amino acid metabolism	5.6750	2.505	**2.29E−4**	3.640
186	↑	4-Hydroxybenzoic acid	Amino acid metabolism	3.7470	1.906	**2.44E−4**	3.613
187	↓	Isomer 1 of 3-Amino-2-piperidone	Amino acid metabolism	0.4663	-1.101	**2.45E−4**	3.611
188	↑	Glutamylproline	Arginine & proline metabolism	2.2843	1.192	**2.45E−4**	3.610
189	↑	Protocatechuic Acid	Benzoate degradation	2.9110	1.542	**2.56E−4**	3.592
190	↑	N-Acetyl-N-Methylserotonin	Tryptophan metabolism	1.9650	0.974	**2.92E−4**	3.534
191	↑	(2R,3R,4R)-2-Amino-4-hydroxy-3-methylpentanoic acid	Amino acid metabolism	3.5142	1.813	**3.13E−4**	3.505
192	↑	Arginine	Amino acid metabolism	1.7005	0.766	**3.61E−4**	3.443
193	↑	Aspartyl-Proline	Arginine & proline metabolism	3.1681	1.664	**3.64E−4**	3.439
194	↑	Alanyl-Aspartic Acid	Alanine, aspartate & glutamate metabolism	2.7777	1.474	**3.83E−4**	3.417
195	↓	4-Chloro-L-lysine	Lysine degradation	0.5774	-0.792	**3.84E−4**	3.416
196	↑	Deoxyeritadenine	Amino acid metabolism	1.9097	0.933	**3.84E−4**	3.416
197	↑	Threoninyl-Aspartate	Protein digestion & absorption	2.5533	1.352	**4.00E−4**	3.398
198	↑	2,3-Diaminosalicylic acid	Amino acid metabolism	2.5697	1.362	**4.11E−4**	3.386
199	↓	Aminofurantoin	Amino acid metabolism	0.5178	-0.949	**4.48E−4**	3.348
200	↑	Cystathionine	Amino acid metabolism	2.7668	1.468	**5.00E−4**	3.301
201	↑	(R)-3,4-Dihydroxymandelonitrile	Amino acid metabolism	1.8141	0.859	**5.11E−4**	3.292
202	↑	Isomer 1 of Ornithine	Arginine & proline metabolism	1.8424	0.882	**5.55E−4**	3.256
203	↑	Tetrahydrobiopterin/L-Threo-Tetrahydrobiopterin	Protein digestion & absorption	2.6751	1.420	**6.08E−4**	3.216
204	↑	5-Methoxytryptophan	Tryptophan metabolism	4.2302	2.081	**6.88E−4**	3.162
205	↑	Norepinephrine	Tyrosine metabolism	4.0731	2.026	**6.92E−4**	3.160
206	↑	Methyl 4-aminobutyrate	Protein digestion & absorption	1.6186	0.695	**7.20E−4**	3.143

Rank	Dir.	Metabolite	Pathway	FC	log_2(FC)	p-value	$-log_{10}$(p)
207	↓	Threoninyl-Threonine	Glycine, serine & threonine metabolism	0.6061	-0.722	**7.24E−4**	3.140
208	↑	Isoleucyl-Glutamate	Alanine, aspartate & glutamate metabolism	1.6852	0.753	**7.39E−4**	3.132
209	↑	Hypoxanthine	Purine metabolism	1.7472	0.805	**7.55E−4**	3.122
210	↑	Valyl-Aspartate	Protein digestion & absorption	2.1617	1.112	**7.96E−4**	3.099
211	↓	Valyl-Arginine	Protein digestion & absorption	0.2799	-1.837	**8.15E−4**	3.089
212	↓	D-Galactosamine 6-phosphate	Amino acid metabolism	0.5269	-0.924	**8.29E−4**	3.081
213	↑	N5-Acetyl-Ornithine	Arginine & proline metabolism	5.0642	2.340	**9.00E−4**	3.046
214	↑	Synephrine	Amino acid metabolism	2.4119	1.270	**9.27E−4**	3.033
215	↓	Epsilon-(Gamma-Glutamyl)-Lysine	Lysine degradation	0.4599	-1.120	**9.89E−4**	3.005
216	↑	L-Cysteinylglycine disulfide	Glutathione metabolism	5.0800	2.345	1.11E−3	2.955
217	↓	Isomer 1 of 5-Aminopentanoic acid	Amino acid metabolism	0.4914	-1.025	1.12E−3	2.951
218	↑	Isomer 1 of 4-Aminobutyraldehyde	Amino acid metabolism	1.7682	0.822	1.26E−3	2.900
219	↓	Isomer 1 of Pyrrolidine	Amino acid metabolism	0.5824	-0.780	1.37E−3	2.864
220	↑	Lysyl-Methionine/Methionyl-Lysine	Cysteine & methionine metabolism	2.0042	1.003	1.42E−3	2.848
221	↓	Phenylalanyl-Threonine	Glycine, serine & threonine metabolism	0.5112	-0.968	1.48E−3	2.829
222	↑	Prolyl-Glycine	Glycine, serine & threonine metabolism	1.8640	0.898	1.56E−3	2.807
223	↓	Glutaminyl-Lysine	Lysine degradation	0.3330	-1.586	1.57E−3	2.805
224	↑	Isoleucyl-Glutamine	Alanine, aspartate & glutamate metabolism	2.4498	1.293	1.57E−3	2.805
225	↑	4-Hydroxynorephedrine	Amino acid metabolism	2.1677	1.116	1.65E−3	2.783
226	↓	Ascorbic acid	Amino acid metabolism	0.5746	-0.799	1.66E−3	2.780
227	↓	2,3-Dihydroxyindole	Amino acid metabolism	0.4962	-1.011	1.67E−3	2.776
228	↑	Leucyl-Proline	Arginine & proline metabolism	1.7316	0.792	1.68E−3	2.774
229	↑	Isomer 1 of Prolyl-Alanine	Alanine, aspartate & glutamate metabolism	1.8385	0.879	1.69E−3	2.772
230	↑	Alanyl-Glycine	Glycine, serine & threonine metabolism	1.5294	0.613	1.73E−3	2.762
231	↓	3-Methoxyanthranilic acid	Protein digestion & absorption	0.5384	-0.893	1.74E−3	2.759
232	↑	Valine	Valine, leucine & isoleucine metabolism	1.7783	0.830	1.75E−3	2.758

Rank	Dir.	Metabolite	Pathway	FC	$\log_2$(FC)	p-value	$-\log_{10}$(p)
233	↓	Arginyl-Isoleucine	Valine, leucine & isoleucine metabolism	0.4667	-1.100	1.80E−3	2.745
234	↑	Glycyl-Valine	Valine, leucine & isoleucine metabolism	1.8725	0.905	1.80E−3	2.744
235	↑	2,8-Dihydroxyadenine	Amino acid metabolism	2.8265	1.499	1.83E−3	2.738
236	↑	O-Acetyl-L-serine hydrochloride	Glycine, serine & threonine metabolism	2.1576	1.109	1.87E−3	2.729
237	↓	4-Formylsalicylic acid	Amino acid metabolism	0.5607	-0.835	1.88E−3	2.727
238	↑	Homoarginine	Arginine & proline metabolism	2.5250	1.336	2.01E−3	2.698
239	↓	Maltol	Amino acid metabolism	0.3675	-1.444	2.03E−3	2.693
240	↑	Histidinyl-Alanine	Alanine, aspartate & glutamate metabolism	2.5230	1.335	2.17E−3	2.663
241	↓	3,4-Dihydroxyphenylacetaldehyde 3-O-glucuronide	Amino acid metabolism	0.3542	-1.497	2.24E−3	2.651
242	↓	Arginyl-Valine	Valine, leucine & isoleucine metabolism	0.3566	-1.488	2.25E−3	2.648
243	↓	2-Deoxy-scyllo-inosamine	Amino acid metabolism	0.6352	-0.655	2.40E−3	2.620
244	↓	3-Hydroxymonoethylglycinexylidide	Glycine, serine & threonine metabolism	0.5908	-0.759	2.56E−3	2.591
245	↑	Valyl-Glycine	Glycine, serine & threonine metabolism	1.9911	0.994	2.58E−3	2.589
246	↓	Lysyl-Phenylalanine	Phenylalanine metabolism	0.5806	-0.784	2.61E−3	2.583
247	↓	3-Hydroxykynurenamine O-sulfate	Amino acid metabolism	0.5047	-0.987	2.69E−3	2.571
248	↓	Isomer 1 of Isoleucyl-Alanine	Alanine, aspartate & glutamate metabolism	0.6099	-0.713	2.74E−3	2.562
249	↓	Isomer 1 of 1-Methylguanosine	Purine metabolism	0.5209	-0.941	2.97E−3	2.527
250	↑	Gly-Norleucine	Valine, leucine & isoleucine metabolism	2.1000	1.070	3.03E−3	2.518
251	↑	(3S,5S)-3,5-Diaminohexanoic acid4	Amino acid metabolism	1.7726	0.826	3.05E−3	2.516
252	↑	Threoninyl-Tryptophan	Tryptophan metabolism	2.0198	1.014	3.06E−3	2.514
253	↓	Isoleucyl-Isoleucine	Valine, leucine & isoleucine metabolism	0.3896	-1.360	3.19E−3	2.496
254	↑	Allocystathionine	Amino acid metabolism	2.9579	1.565	3.61E−3	2.442
255	↑	5-Hydroxyindoleacetylglycine	Glycine, serine & threonine metabolism	2.4829	1.312	3.66E−3	2.436
256	↑	Threoninyl-Alanine	Alanine, aspartate & glutamate metabolism	1.9741	0.981	3.73E−3	2.428
257	↑	4-Nitrocatechol	Amino acid metabolism	1.5918	0.671	3.74E−3	2.427
258	↓	N-Ethylglycine	Glycine, serine & threonine metabolism	0.5351	-0.902	3.77E−3	2.424

Rank	Dir.	Metabolite	Pathway	FC	log₂(FC)	p-value	−log₁₀(p)
259	↑	3-Amino-4-hydroxybenzoic acid	Amino acid metabolism	3.1876	1.672	3.92E−3	2.407
260	↑	Alanyl-Asparagine	Alanine, aspartate & glutamate metabolism	1.5099	0.594	3.98E−3	2.400
261	↓	Glutamyl-Leucine	Valine, leucine & isoleucine metabolism	0.5289	-0.919	4.04E−3	2.394
262	↑	2-Pyrocatechuic Acid	Amino acid metabolism	2.0118	1.008	4.09E−3	2.388
263	↑	Glutaminyl-Tryptophan	Tryptophan metabolism	2.2450	1.167	4.38E−3	2.358
264	↑	Glutaminyl-Methionine	Cysteine & methionine metabolism	2.4245	1.278	4.41E−3	2.356
265	↑	Cytidine	Pyrimidine metabolism	2.4338	1.283	4.42E−3	2.354
266	↑	Arginyl-Glycine	Glycine, serine & threonine metabolism	1.8587	0.894	4.43E−3	2.353
267	↑	Seryl-Serine	Glycine, serine & threonine metabolism	2.0148	1.011	4.64E−3	2.334
268	↑	Glycyl-Threonine	Glycine, serine & threonine metabolism	2.3824	1.252	4.69E−3	2.328
269	↑	Piceatannol	Amino acid metabolism	2.1357	1.095	4.74E−3	2.324
270	↓	3-(2,3-Dihydroxyphenyl)propanoic acid	Amino acid metabolism	0.5878	-0.767	4.84E−3	2.315
271	↑	Isoleucyl-Hydroxyproline	Arginine & proline metabolism	2.0862	1.061	5.24E−3	2.281
272	↑	Asparaginyl-Threonine	Glycine, serine & threonine metabolism	1.7810	0.833	5.67E−3	2.246
273	↑	Isomer 1 of (2R,3R,4R)-2-Amino-4-hydroxy-3-methylpentanoic acid	Amino acid metabolism	1.9204	0.941	5.88E−3	2.230
274	↑	Sarcosine	Glycine, serine & threonine metabolism	1.6497	0.722	6.39E−3	2.195
275	↓	N-Acetyl-1-(5'-Phosphoribosyl)-5-amino-4-imidazolecarboxamide	Amino acid metabolism	0.1783	-2.488	6.41E−3	2.193
276	↓	3,4-Dihydroxyphenylacetaldehyde 4-O-glucuronide	Amino acid metabolism	0.5191	-0.946	6.76E−3	2.170
277	↑	N6-Acetyl-Lysine	Lysine degradation	1.8453	0.884	7.82E−3	2.107
278	↑	Hydroxyprolyl-Serine	Glycine, serine & threonine metabolism	1.6738	0.743	7.89E−3	2.103
279	↑	4-Hydroxybenzaldehyde/3-Hydroxybenzaldehyde	Tyrosine metabolism	3.0598	1.613	7.97E−3	2.098
280	↑	(2R,3R)-3-Methylornithine	Arginine & proline metabolism	1.8830	0.913	8.00E−3	2.097
281	↓	3,4-Dihydroxymandelic acid	Amino acid metabolism	0.5680	-0.816	8.34E−3	2.079
282	↑	Lysyl-Glycine	Glycine, serine & threonine metabolism	1.8233	0.867	8.50E−3	2.070
283	↑	Aminoadipic Acid	Amino acid metabolism	2.0415	1.030	8.93E−3	2.049
284	↑	Isoleucyl-Threonine	Glycine, serine & threonine metabolism	1.5329	0.616	9.12E−3	2.040

Rank	Dir.	Metabolite	Pathway	FC	$\log_2$(FC)	p-value	$-\log_{10}$(p)
285	↓	Creatine	Arginine & proline metabolism	0.5246	-0.931	9.30E−3	2.032
286	↑	(E)-4-Hydroxyphenylacetaldehyde Oxime	Amino acid metabolism	2.0521	1.037	9.35E−3	2.029
287	↑	Arginyl-Serine	Glycine, serine & threonine metabolism	2.4881	1.315	9.86E−3	2.006
288	↑	Serotonin	Tryptophan metabolism	1.7807	0.832	1.02E−2	1.992
289	↑	Isomer 1 of Histidine	Histidine metabolism	2.0297	1.021	1.03E−2	1.987
290	↑	7,8-Dihydroneopterin/7,8-Dihydromonapterin	Amino acid metabolism	1.6742	0.743	1.06E−2	1.974
291	↓	Alanyl-Tyrosine	Protein digestion & absorption	0.0982	-3.348	1.09E−2	1.963
292	↑	Taurine	Taurine & hypotaurine metabolism	2.8966	1.534	1.15E−2	1.938
293	↑	Lysyl-Alanine	Alanine, aspartate & glutamate metabolism	1.7654	0.820	1.21E−2	1.916
294	↑	Threoninyl-Glutamine	Alanine, aspartate & glutamate metabolism	1.5600	0.642	1.35E−2	1.868
295	↑	Pipecolic Acid	Amino acid metabolism	1.8423	0.881	1.47E−2	1.834
296	↓	N2-Acetyl-5'-Hydroxykynurenamine	Amino acid metabolism	0.5175	-0.950	1.53E−2	1.816
297	↓	Caffeic acid	Amino acid metabolism	0.6142	-0.703	1.54E−2	1.813
298	↑	1-Methylhistidine	Histidine metabolism	1.8095	0.856	1.55E−2	1.811
299	↑	Dtdp-3-Amino-3,6-Dideoxy-D-Glucose/Dtdp-3-Amino-3,6-Dideoxy-Alpha-D-Galactopyranose	Protein digestion & absorption	2.7059	1.436	1.65E−2	1.782
300	↑	Arginyl-Histidine	Histidine metabolism	1.6122	0.689	1.69E−2	1.771
301	↓	5,6-Dihydroxyindole	Amino acid metabolism	0.6562	-0.608	1.76E−2	1.754
302	↓	5-Hydroxykynurenamine	Tryptophan metabolism	0.4868	-1.039	1.77E−2	1.753
303	↑	Glutamyl-Serine/Seryl-Glutamate	Alanine, aspartate & glutamate metabolism	1.9313	0.950	1.82E−2	1.739
304	↓	Pyrocatechol sulfate	Amino acid metabolism	0.6489	-0.624	2.02E−2	1.695
305	↑	4-Hydroxyproline	Arginine & proline metabolism	1.9921	0.994	2.05E−2	1.689
306	↑	Isomer 1 of 5-Hydroxyectoine	Amino acid metabolism	1.6648	0.735	2.49E−2	1.603
307	↓	Threoninyl-Lysine	Lysine degradation	0.5749	-0.799	2.50E−2	1.602
308	↓	Glycyl-Lysine	Lysine degradation	0.4518	-1.146	2.58E−2	1.588
309	↑	Glycyl-Tyrosine	Protein digestion & absorption	1.5498	0.632	2.98E−2	1.526

Rank	Dir.	Metabolite	Pathway	FC	$\log_2(FC)$	p-value	$-\log_{10}(p)$
310	↑	Valyl-Asparagine	Alanine, aspartate & glutamate metabolism	1.5034	0.588	3.18E−2	1.498
311	↑	Leucyl-Glutamine	Alanine, aspartate & glutamate metabolism	1.6822	0.750	3.34E−2	1.477
312	↓	Leucyl-Lysine	Lysine degradation	0.3277	-1.610	3.37E−2	1.473
313	↑	Hemigossypol	Amino acid metabolism	1.5858	0.665	3.37E−2	1.472
314	↓	N-(L-Arginino)Succinic Acid	Protein digestion & absorption	0.4208	-1.249	3.69E−2	1.433
315	↑	Hydroquinone	Amino acid metabolism	1.5347	0.618	4.30E−2	1.366
316	↓	Cyclic pyranopterin monophosphate	Amino acid metabolism	0.5434	-0.880	4.74E−2	1.324

Significance criteria: $p < 0.05$ (Welch's two-tailed t-test) AND $|FC| > 1.5$. Metabolites ranked by ascending p-value. FC = fold change of first group relative to second. ↑ = increased; ↓ = decreased. $\log_2(FC)$ and $-\log_{10}(p)$ provided for volcano plot reference. Pathway annotations based on KEGG Bos taurus metabolic library. Source data: metaboan2.csv ($n = 10$ per group; 474 metabolites after Tier 3 exclusions).

Table S7. Longitudinal changes in healthy quarters: H-D21 vs H-D2 (n = 316 significantly altered metabolites), ranked by ascending p-value.

Rank	Dir.	Metabolite	Pathway	FC	$log_2(FC)$	p-value	$-log_{10}(p)$
1	↑	Aspartic acid	Alanine, aspartate & glutamate metabolism	7.0730	2.822	**1.12E−14**	13.949
2	↑	Protocatechuic Acid	Benzoate degradation	6.2830	2.652	**4.30E−13**	12.367
3	↓	N-Acetyl-3-Hydroxyanthranilic acid	Tryptophan metabolism	0.1481	-2.755	**1.71E−12**	11.767
4	↑	N-Acetylindoxyl	Tryptophan metabolism	16.0020	4.000	**1.95E−12**	11.710
5	↓	Lysyl-Valine	Valine, leucine & isoleucine metabolism	0.1563	-2.677	**6.70E−12**	11.174
6	↑	L-Cysteinylglycine disulfide	Glutathione metabolism	10.3790	3.376	**4.20E−11**	10.376
7	↑	Glutamyl-Histidine	Histidine metabolism	3.6886	1.883	**4.50E−11**	10.346
8	↑	Leucine	Valine, leucine & isoleucine metabolism	5.8212	2.541	**5.60E−11**	10.252
9	↑	4-Amino-2-hydroxylamino-6-nitrotoluene	Amino acid metabolism	8.2308	3.041	**6.11E−11**	10.214
10	↓	S-Cysteinosuccinic acid	Protein digestion & absorption	0.2228	-2.166	**6.54E−11**	10.185
11	↑	4-Hydroxystyrene	Amino acid metabolism	7.5590	2.918	**6.70E−11**	10.174
12	↓	Diethanolamine	Glycerophospholipid metabolism	0.1631	-2.616	**8.92E−11**	10.050
13	↑	4-Oxoproline	Arginine & proline metabolism	7.4332	2.894	**1.24E−10**	9.907
14	↓	Valyl-Lysine	Lysine degradation	0.1387	-2.850	**1.63E−10**	9.787
15	↓	Pantothenic Acid	Pantothenate & CoA biosynthesis	0.0869	-3.525	**2.09E−10**	9.681
16	↑	Seryl-Valine	Valine, leucine & isoleucine metabolism	3.0837	1.625	**2.36E−10**	9.626
17	↓	Pyrrolidine	Amino acid metabolism	0.1463	-2.773	**2.44E−10**	9.613
18	↓	Isomer 1 of 4-Chloro-L-lysine	Lysine degradation	0.1451	-2.785	**3.27E−10**	9.485
19	↑	(E)-6'-Hydroxyferulate	Amino acid metabolism	3.8326	1.938	**3.36E−10**	9.474
20	↑	4-Guanidinobutanal	Amino acid metabolism	5.4468	2.445	**3.49E−10**	9.457
21	↑	Phenylalanine	Phenylalanine metabolism	4.9995	2.322	**3.60E−10**	9.444
22	↓	Tyrosyl-Leucine	Valine, leucine & isoleucine metabolism	0.0672	-3.896	**3.80E−10**	9.421
23	↓	Arginyl-Leucine	Valine, leucine & isoleucine metabolism	0.1046	-3.257	**4.02E−10**	9.395
24	↑	N(6)-Methyllysine	Lysine degradation	9.1492	3.194	**5.73E−10**	9.242

Rank	Dir.	Metabolite	Pathway	FC	log₂(FC)	p-value	−log₁₀(p)
25	↑	7,8-Dihydroxykynurenic acid	Tryptophan metabolism	4.0517	2.018	**6.73E−10**	9.172
26	↑	Lysyl-Aspartate	Protein digestion & absorption	3.7286	1.899	**9.44E−10**	9.025
27	↑	Seryl-Alanine	Alanine, aspartate & glutamate metabolism	4.5396	2.183	**1.12E−9**	8.949
28	↑	Octopamine	Tyrosine metabolism	4.4639	2.158	**1.16E−9**	8.934
29	↑	Methionine	Cysteine & methionine metabolism	4.2920	2.102	**1.20E−9**	8.920
30	↑	Alanyl-Alanine	Alanine, aspartate & glutamate metabolism	7.7678	2.958	**1.38E−9**	8.861
31	↓	Isomer 1 of p-Hydroxyphenylacetylglycine	Glycine, serine & threonine metabolism	0.3046	-1.715	**1.41E−9**	8.850
32	↓	Creatine	Arginine & proline metabolism	0.2810	-1.832	**1.51E−9**	8.821
33	↑	Glycyl-Threonine	Glycine, serine & threonine metabolism	3.2566	1.703	**1.51E−9**	8.820
34	↑	2-Amino-2-methyl-1,3-propanediol	Amino acid metabolism	2.8935	1.533	**1.92E−9**	8.718
35	↑	3,4-Dihydroxystyrene	Amino acid metabolism	8.3298	3.058	**2.63E−9**	8.580
36	↑	L-Isoleucine	Valine, leucine & isoleucine metabolism	4.4826	2.164	**3.28E−9**	8.484
37	↑	5-Aminolevulinic acid	Amino acid metabolism	8.2326	3.041	**4.05E−9**	8.392
38	↓	Leucyl-Leucine	Valine, leucine & isoleucine metabolism	0.2806	-1.833	**6.23E−9**	8.206
39	↓	N-Ethylglycine	Glycine, serine & threonine metabolism	0.3402	-1.556	**1.15E−8**	7.941
40	↓	Choline	Glycerophospholipid metabolism	0.1138	-3.136	**1.25E−8**	7.902
41	↑	Dehydroalanine	Alanine, aspartate & glutamate metabolism	2.9476	1.560	**1.27E−8**	7.896
42	↑	Adrenochrome o-semiquinone	Tyrosine metabolism	8.1475	3.026	**1.54E−8**	7.814
43	↑	Isoleucyl-Glycine	Glycine, serine & threonine metabolism	4.7219	2.239	**1.61E−8**	7.793
44	↑	Aspartyl-Aspartate	Protein digestion & absorption	2.9528	1.562	**1.77E−8**	7.751
45	↑	5-Hydroxyectoine	Amino acid metabolism	3.9572	1.984	**1.88E−8**	7.726
46	↑	3-Amino-3-(4-hydroxyphenyl)propanoic acid	Amino acid metabolism	5.7146	2.515	**2.22E−8**	7.654
47	↑	Methionine Sulfoxide	Cysteine & methionine metabolism	4.0533	2.019	**2.24E−8**	7.650
48	↑	Tyrosine	Amino acid metabolism	5.6734	2.504	**2.65E−8**	7.576
49	↑	Tyrosyl-Glycine	Glycine, serine & threonine metabolism	5.4417	2.444	**2.68E−8**	7.571
50	↑	2'-Hydroxyacetophenone	Amino acid metabolism	4.1451	2.051	**3.16E−8**	7.500

Rank	Dir.	Metabolite	Pathway	FC	$\log_2$(FC)	p-value	$-\log_{10}$(p)
51	↓	Leucocyanidin	Amino acid metabolism	0.2410	-2.053	**3.19E−8**	7.497
52	↓	alpha-D-Glucosamine 1-phosphate	Protein digestion & absorption	0.2202	-2.183	**3.25E−8**	7.488
53	↓	Isomer 2 of 5-Aminopentanoic acid	Amino acid metabolism	0.2154	-2.215	**3.44E−8**	7.464
54	↑	Cystine	Cysteine & methionine metabolism	3.5444	1.826	**3.82E−8**	7.418
55	↑	Uracil	Pyrimidine metabolism	108.3600	6.760	**4.19E−8**	7.378
56	↓	D-Galactosamine 6-phosphate	Amino acid metabolism	0.3567	-1.487	**5.07E−8**	7.295
57	↑	Gly-Norleucine	Valine, leucine & isoleucine metabolism	3.6467	1.867	**5.13E−8**	7.290
58	↑	4-Aminobutyraldehyde	Amino acid metabolism	3.2387	1.695	**6.06E−8**	7.218
59	↑	1,4-Diaminobutane	Amino acid metabolism	13.3650	3.740	**6.91E−8**	7.160
60	↓	L-2-Amino-3-(4-aminophenyl)propanoic acid	Amino acid metabolism	0.2124	-2.235	**7.04E−8**	7.152
61	↑	N8-Acetylspermidine	Amino acid metabolism	4.2539	2.089	**7.56E−8**	7.121
62	↑	Lysyl-Alanine	Alanine, aspartate & glutamate metabolism	4.1862	2.066	**9.02E−8**	7.045
63	↑	Asparaginyl-Threonine	Glycine, serine & threonine metabolism	2.0654	1.046	**9.18E−8**	7.037
64	↓	4-Formylsalicylic acid	Amino acid metabolism	0.4304	-1.216	**1.03E−7**	6.989
65	↑	2,6-Diamino-4-hydroxy-5-N-methylformamidopyrimidine	Amino acid metabolism	4.1684	2.060	**1.27E−7**	6.895
66	↓	(S)-2-Aminobutanoic Acid	Amino acid metabolism	0.4366	-1.196	**1.52E−7**	6.818
67	↓	Ethanolamine	Glycerophospholipid metabolism	0.2043	-2.291	**1.67E−7**	6.776
68	↓	Isomer 1 of 3-Amino-2-piperidone	Amino acid metabolism	0.2435	-2.038	**2.19E−7**	6.659
69	↑	Lysyl-Serine	Glycine, serine & threonine metabolism	6.0540	2.598	**2.67E−7**	6.574
70	↑	Cis-4-Hydroxy-D-Proline	Arginine & proline metabolism	6.3478	2.666	**2.82E−7**	6.549
71	↓	5-Hydroxylysine	Lysine degradation	0.1531	-2.707	**3.04E−7**	6.517
72	↑	2-Aminophenol	Amino acid metabolism	3.1731	1.666	**3.43E−7**	6.464
73	↑	Threoninyl-Glutamine	Alanine, aspartate & glutamate metabolism	2.6772	1.421	**3.47E−7**	6.460
74	↓	7,8-Dihydroxanthopterin	Amino acid metabolism	0.1464	-2.772	**3.53E−7**	6.453
75	↑	1H-Indole-3-methanamine	Amino acid metabolism	3.0355	1.602	**3.61E−7**	6.442
76	↑	3-Hydroxymelatonin	Amino acid metabolism	3.0366	1.602	**3.63E−7**	6.440

Rank	Dir.	Metabolite	Pathway	FC	log₂(FC)	p-value	−log₁₀(p)
77	↑	p-Aminobenzoic acid	Amino acid metabolism	2.2348	1.160	**3.93E−7**	6.406
78	↑	Threoninyl-Alanine	Alanine, aspartate & glutamate metabolism	2.4874	1.315	**4.13E−7**	6.384
79	↓	3-Hydroxykynurenamine	Amino acid metabolism	0.1856	-2.430	**4.24E−7**	6.372
80	↑	Histidinyl-Glutamate	Alanine, aspartate & glutamate metabolism	10.5990	3.406	**4.29E−7**	6.367
81	↑	Aspartyl-Lysine	Lysine degradation	2.5016	1.323	**4.65E−7**	6.332
82	↑	gamma-Glutamyl-Proline	Arginine & proline metabolism	2.7459	1.457	**4.94E−7**	6.307
83	↓	Carbapenem biosynthesis intermediate 3	Amino acid metabolism	0.3370	-1.569	**5.16E−7**	6.287
84	↑	Alanyl-Phenylalanine	Phenylalanine metabolism	3.3960	1.764	**6.77E−7**	6.170
85	↑	Isomer 1 of Sarcosine	Glycine, serine & threonine metabolism	7.4581	2.899	**6.94E−7**	6.159
86	↑	2-Hydroxyhepta-2,4-dienedioic acid	Amino acid metabolism	3.4882	1.802	**7.55E−7**	6.122
87	↑	Valyl-Glutamate	Alanine, aspartate & glutamate metabolism	6.7424	2.753	**7.59E−7**	6.120
88	↓	Amoxicillin	Amino acid metabolism	0.1723	-2.537	**7.73E−7**	6.112
89	↓	Isomer 1 of Isoleucyl-Alanine	Alanine, aspartate & glutamate metabolism	0.4217	-1.246	**7.85E−7**	6.105
90	↑	Histidine	Histidine metabolism	4.0373	2.013	**7.89E−7**	6.103
91	↑	Threoninyl-Aspartate	Protein digestion & absorption	4.3575	2.124	**7.95E−7**	6.100
92	↑	Isomer 1 of Arginine	Amino acid metabolism	1.9484	0.962	**7.97E−7**	6.099
93	↓	Pyrocatechol sulfate	Amino acid metabolism	0.4702	-1.089	**8.03E−7**	6.095
94	↑	Arginyl-Glycine	Glycine, serine & threonine metabolism	3.5086	1.811	**8.40E−7**	6.076
95	↑	2-Hydroxy-2,4-Pentadienoic Acid	Amino acid metabolism	3.7590	1.910	**1.01E−6**	5.997
96	↓	Isomer 1 of 5-Aminopentanoic acid	Amino acid metabolism	0.3008	-1.733	**1.01E−6**	5.996
97	↓	Prolyl-Aspartate	Protein digestion & absorption	0.3980	-1.329	**1.28E−6**	5.891
98	↓	Aminofurantoin	Amino acid metabolism	0.4188	-1.256	**1.30E−6**	5.887
99	↑	Valyl-Alanine	Alanine, aspartate & glutamate metabolism	3.7661	1.913	**1.49E−6**	5.828
100	↓	Alanyl-Methionine	Cysteine & methionine metabolism	0.1430	-2.806	**1.53E−6**	5.816
101	↓	Isomer 2 of 4-Chloro-L-lysine	Lysine degradation	0.2001	-2.321	**1.61E−6**	5.794
102	↑	Alanyl-Glutamine	Alanine, aspartate & glutamate metabolism	4.0002	2.000	**1.63E−6**	5.788

Rank	Dir.	Metabolite	Pathway	FC	log_2(FC)	p-value	$-log_{10}$(p)
103	↓	Threonine	Glycine, serine & threonine metabolism	0.1780	-2.490	**1.69E−6**	5.773
104	↓	N-formylkynurenine	Tryptophan metabolism	0.2428	-2.042	**2.03E−6**	5.693
105	↑	Salicylamide	Amino acid metabolism	2.1754	1.121	**2.09E−6**	5.680
106	↑	4-Aminohippuric acid	Purine metabolism	2.0294	1.021	**2.09E−6**	5.680
107	↑	N-gamma-Glutamyl-S-allylcysteine	Protein digestion & absorption	4.1110	2.039	**2.51E−6**	5.600
108	↑	1-Aminocyclopropane-1-Carboxylic Acid	Amino acid metabolism	5.1978	2.378	**2.53E−6**	5.597
109	↑	(R)-3,4-Dihydroxymandelonitrile	Amino acid metabolism	2.1957	1.135	**2.57E−6**	5.589
110	↑	4-O-Methylgallic acid	Protein digestion & absorption	4.5111	2.174	**2.84E−6**	5.546
111	↑	3-O-Methylgallic Acid	Protein digestion & absorption	6.1118	2.612	**2.87E−6**	5.542
112	↑	Aspartyl-Valine	Valine, leucine & isoleucine metabolism	4.9888	2.319	**3.10E−6**	5.509
113	↓	Nocardicin G	Amino acid metabolism	0.2186	-2.193	**3.41E−6**	5.468
114	↑	Beta-Ethynylserine	Glycine, serine & threonine metabolism	5.9918	2.583	**4.66E−6**	5.331
115	↑	Norsalsolinol	Tyrosine metabolism	2.6958	1.431	**4.75E−6**	5.323
116	↓	3,4-Dihydroxyphenylethyleneglycol 3-O-glucuronide	Amino acid metabolism	0.2730	-1.873	**4.84E−6**	5.315
117	↑	O-Acetyl-Homoserine	Glycine, serine & threonine metabolism	2.2890	1.195	**5.13E−6**	5.290
118	↓	Salsolinol 1-carboxylic acid	Tyrosine metabolism	0.3890	-1.362	**5.78E−6**	5.238
119	↓	Mangiferin	Flavonoid biosynthesis	0.3064	-1.706	**6.36E−6**	5.196
120	↓	S-Adenosyl-L-homocysteine	Cysteine & methionine metabolism	0.2455	-2.026	**7.05E−6**	5.152
121	↑	Isomer 1 of Histidine	Histidine metabolism	2.9596	1.565	**7.11E−6**	5.148
122	↑	Hypoxanthine	Purine metabolism	2.4278	1.280	**7.30E−6**	5.137
123	↑	Histidinyl-Alanine	Alanine, aspartate & glutamate metabolism	4.2333	2.082	**7.49E−6**	5.126
124	↑	Alanyl-Aspartic Acid	Alanine, aspartate & glutamate metabolism	4.9431	2.305	**7.90E−6**	5.102
125	↑	Seryl-Tyrosine	Protein digestion & absorption	5.3032	2.407	**8.09E−6**	5.092
126	↑	1-Methylguanosine	Purine metabolism	2.8197	1.496	**9.09E−6**	5.042
127	↑	Asparaginyl-Serine	Glycine, serine & threonine metabolism	5.5636	2.476	**9.55E−6**	5.020
128	↑	N-Acetylputrescine	Amino acid metabolism	1.9471	0.961	**1.05E−5**	4.980

Rank	Dir.	Metabolite	Pathway	FC	$\log_2$(FC)	p-value	$-\log_{10}$(p)
129	↑	Isoleucyl-Threonine	Glycine, serine & threonine metabolism	2.9430	1.557	**1.08E−5**	4.965
130	↑	Isomer 2 of Prolyl-Alanine	Alanine, aspartate & glutamate metabolism	2.0021	1.002	**1.12E−5**	4.952
131	↑	Tryptophyl-Aspartate	Protein digestion & absorption	2.8125	1.492	**1.37E−5**	4.863
132	↑	Aspartyl-Proline	Arginine & proline metabolism	2.3656	1.242	**1.41E−5**	4.850
133	↑	Glutamyl-Aspartate	Protein digestion & absorption	3.2960	1.721	**1.44E−5**	4.843
134	↓	2-Hydroxymuconate Semialdehyde	Amino acid metabolism	0.1981	-2.336	**1.59E−5**	4.799
135	↓	Histidinyl-Isoleucine	Valine, leucine & isoleucine metabolism	0.3916	-1.353	**1.64E−5**	4.784
136	↓	Galactosylhydroxylysine	Lysine degradation	0.2733	-1.871	**1.83E−5**	4.736
137	↑	6-Lactoyl-5,6,7,8-Tetrahydropterin	Amino acid metabolism	2.7315	1.450	**1.91E−5**	4.718
138	↑	Leucyl-Glutamine	Alanine, aspartate & glutamate metabolism	3.6841	1.881	**2.04E−5**	4.689
139	↑	Serotonin	Tryptophan metabolism	3.0518	1.610	**2.17E−5**	4.664
140	↑	Cytidine	Pyrimidine metabolism	4.7027	2.234	**2.28E−5**	4.642
141	↑	Threoninyl-Glutamate	Alanine, aspartate & glutamate metabolism	2.7657	1.468	**2.35E−5**	4.629
142	↑	Glycyl-Asparagine	Alanine, aspartate & glutamate metabolism	2.4944	1.319	**2.55E−5**	4.593
143	↑	Hydroxy-lacosamide	Amino acid metabolism	1.7669	0.821	**2.60E−5**	4.585
144	↑	p-Coumaroylputrescine	Amino acid metabolism	4.4273	2.146	**2.82E−5**	4.549
145	↑	Valyl-Glycine	Glycine, serine & threonine metabolism	1.9489	0.963	**3.11E−5**	4.508
146	↑	Piceatannol	Amino acid metabolism	3.1430	1.652	**3.13E−5**	4.505
147	↓	1,4-Dihydroxy-6-naphthoic acid	Amino acid metabolism	0.2981	-1.746	**3.17E−5**	4.498
148	↑	Glutamyl-Alanine	Alanine, aspartate & glutamate metabolism	2.1023	1.072	**3.18E−5**	4.497
149	↑	Tyrosyl-Proline	Arginine & proline metabolism	3.7104	1.892	**3.20E−5**	4.494
150	↑	Hydroquinone	Amino acid metabolism	2.2894	1.195	**3.61E−5**	4.442
151	↑	D-histidine methyl ester	Histidine metabolism	2.1468	1.102	**3.71E−5**	4.431
152	↑	2-Hydroxy-4-imino-2,5-cyclohexadienone	Amino acid metabolism	1.8301	0.872	**4.40E−5**	4.356
153	↓	4-Amino-4-Deoxychorismic Acid	Amino acid metabolism	0.3903	-1.357	**4.56E−5**	4.341
154	↑	Lysyl-Glutamine	Alanine, aspartate & glutamate metabolism	2.5780	1.366	**4.81E−5**	4.318

Rank	Dir.	Metabolite	Pathway	FC	$\log_2$(FC)	p-value	$-\log_{10}$(p)
155	↑	Arginyl-Alanine	Alanine, aspartate & glutamate metabolism	2.3989	1.262	**4.89E−5**	4.311
156	↑	Glutamic acid	Amino acid metabolism	2.6513	1.407	**5.29E−5**	4.276
157	↓	3-Methoxy-4-hydroxyphenylglycolaldehyde	Protein digestion & absorption	0.4971	-1.008	**5.31E−5**	4.275
158	↓	Benzene-1,2,4-Triol	Amino acid metabolism	0.2915	-1.778	**5.85E−5**	4.233
159	↑	(3S,5S)-3,5-Diaminohexanoic acid4	Amino acid metabolism	1.9535	0.966	**6.03E−5**	4.220
160	↑	Lysyl-Glycine	Glycine, serine & threonine metabolism	2.2195	1.150	**6.34E−5**	4.198
161	↓	Asparagine	Alanine, aspartate & glutamate metabolism	0.4340	-1.204	**7.27E−5**	4.139
162	↑	N-Methyl aspartate	Protein digestion & absorption	2.0092	1.007	**7.37E−5**	4.132
163	↑	2,6-Dihydroxypseudooxynicotine	Amino acid metabolism	1.8557	0.892	**7.37E−5**	4.132
164	↑	7-Cyano-7-Carbaguanine	Amino acid metabolism	8.6716	3.116	**7.97E−5**	4.099
165	↑	Seryl-Lysine	Lysine degradation	5.5308	2.468	**8.71E−5**	4.060
166	↑	2-Pyrocatechuic Acid	Amino acid metabolism	2.2659	1.180	**8.84E−5**	4.053
167	↑	2-(3-Carboxy-3-Aminopropyl)-L-Histidine	Histidine metabolism	4.5942	2.200	**9.55E−5**	4.020
168	↑	Homocitrulline	Arginine & proline metabolism	2.0638	1.045	**1.07E−4**	3.971
169	↑	Prolyl-Alanine	Alanine, aspartate & glutamate metabolism	2.3608	1.239	**1.10E−4**	3.957
170	↓	Pyridoxal	Vitamin B6 metabolism	0.1724	-2.536	**1.11E−4**	3.955
171	↑	4-Hydroxybenzaldehyde/3-Hydroxybenzaldehyde	Tyrosine metabolism	6.1514	2.621	**1.13E−4**	3.948
172	↑	Valyl-Serine	Glycine, serine & threonine metabolism	1.6852	0.753	**1.14E−4**	3.944
173	↑	Hydroxyprolyl-Serine	Glycine, serine & threonine metabolism	2.8077	1.489	**1.16E−4**	3.935
174	↑	2,3-Diaminosalicylic acid	Amino acid metabolism	2.8139	1.493	**1.21E−4**	3.919
175	↑	2,6-Dihydroxybenzoic acid	Amino acid metabolism	1.9174	0.939	**1.27E−4**	3.896
176	↑	Lysyl-Threonine	Glycine, serine & threonine metabolism	2.7978	1.484	**1.29E−4**	3.889
177	↑	Methyl 4-aminobutyrate	Protein digestion & absorption	1.6483	0.721	**1.46E−4**	3.836
178	↑	7,8-Dihydroneopterin/7,8-Dihydromonapterin	Amino acid metabolism	1.7922	0.842	**1.51E−4**	3.820
179	↑	Isomer 1 of Benzocaine	Amino acid metabolism	3.1201	1.642	**1.55E−4**	3.811
180	↓	3-(2,3-Dihydroxyphenyl)propanoic acid	Amino acid metabolism	0.4040	-1.308	**1.77E−4**	3.751

Rank	Dir.	Metabolite	Pathway	FC	log_2(FC)	p-value	$-log_{10}$(p)
181	↑	p-Hydroxyphenylacetic acid	Amino acid metabolism	1.5855	0.665	**1.87E−4**	3.729
182	↑	(2R,4S)-2,4-Diaminopentanoic Acid	Amino acid metabolism	2.7517	1.460	**1.94E−4**	3.712
183	↑	Seryl-Threonine	Glycine, serine & threonine metabolism	2.1044	1.073	**2.06E−4**	3.687
184	↓	Glutamine	Alanine, aspartate & glutamate metabolism	0.4935	-1.019	**2.09E−4**	3.679
185	↑	Isomer 1 of Iminodiacetic acid	Amino acid metabolism	1.9001	0.926	**2.11E−4**	3.676
186	↑	5-Hydroxykynurenine	Tryptophan metabolism	2.0643	1.046	**2.18E−4**	3.661
187	↑	Ornithine	Arginine & proline metabolism	2.6893	1.427	**2.26E−4**	3.647
188	↓	Atenolol	Amino acid metabolism	0.5318	-0.911	**2.34E−4**	3.630
189	↑	Lysyl-Methionine/Methionyl-Lysine	Cysteine & methionine metabolism	3.8327	1.938	**2.39E−4**	3.622
190	↓	2,3-Dihydroxycarbamazepine	Amino acid metabolism	0.3846	-1.379	**2.41E−4**	3.618
191	↑	3,4-Dihydroxybenzeneacetic acid	Amino acid metabolism	1.5747	0.655	**2.43E−4**	3.615
192	↑	Seryl-Cysteine	Protein digestion & absorption	2.3818	1.252	**2.44E−4**	3.613
193	↓	Glutaminyl-Proline/Prolyl-Glutamine	Arginine & proline metabolism	0.6176	-0.695	**2.48E−4**	3.606
194	↑	Glutaminyl-Aspartic Acid	Alanine, aspartate & glutamate metabolism	3.3791	1.757	**2.75E−4**	3.561
195	↑	Tryptophan	Tryptophan metabolism	3.9264	1.973	**2.76E−4**	3.559
196	↑	Threoninyl-Glycine	Glycine, serine & threonine metabolism	2.5094	1.327	**2.83E−4**	3.548
197	↑	Valyl-Threonine	Glycine, serine & threonine metabolism	2.1385	1.097	**2.85E−4**	3.545
198	↑	Glycyl-Leucine	Valine, leucine & isoleucine metabolism	4.8833	2.288	**2.91E−4**	3.536
199	↑	4-Hydroxybenzoic acid	Amino acid metabolism	2.6680	1.416	**2.92E−4**	3.534
200	↓	3,4-Dihydroxybenzylamine	Amino acid metabolism	0.3869	-1.370	**2.92E−4**	3.534
201	↑	Glutamyl-Serine/Seryl-Glutamate	Alanine, aspartate & glutamate metabolism	2.7699	1.470	**2.96E−4**	3.528
202	↑	Alanyl-Glycine	Glycine, serine & threonine metabolism	2.4755	1.308	**3.10E−4**	3.508
203	↑	Cystathionine	Amino acid metabolism	1.8612	0.896	**3.42E−4**	3.466
204	↓	Maltol	Amino acid metabolism	0.3100	-1.690	**3.44E−4**	3.463
205	↑	Isoleucyl-Glutamate	Alanine, aspartate & glutamate metabolism	3.4319	1.779	**3.73E−4**	3.428
206	↑	Isoleucyl-Glutamine	Alanine, aspartate & glutamate metabolism	2.6312	1.396	**3.85E−4**	3.415

Rank	Dir.	Metabolite	Pathway	FC	log_2(FC)	p-value	$-log_{10}$(p)
207	↑	Serylglutamic Acid	Protein digestion & absorption	5.0379	2.333	**3.93E−4**	3.405
208	↑	Glycyl-Valine	Valine, leucine & isoleucine metabolism	2.5746	1.364	**3.94E−4**	3.405
209	↑	Xanthurenic acid	Amino acid metabolism	2.4306	1.281	**4.26E−4**	3.371
210	↑	Valyl-Asparagine	Alanine, aspartate & glutamate metabolism	2.3643	1.241	**5.14E−4**	3.289
211	↑	Prolyl-Isoleucine	Valine, leucine & isoleucine metabolism	2.9498	1.561	**5.37E−4**	3.270
212	↑	N-Acetyl-L-Tyrosine	Protein digestion & absorption	2.2200	1.150	**5.82E−4**	3.235
213	↑	Neopterin	Amino acid metabolism	1.5731	0.654	**6.08E−4**	3.216
214	↑	Leucyl-Threonine	Glycine, serine & threonine metabolism	3.6528	1.869	**6.63E−4**	3.179
215	↓	3-Cyano-Alanine	Cyanoamino acid metabolism	0.6368	-0.651	**6.81E−4**	3.167
216	↑	di-Hydroxymelatonin	Amino acid metabolism	1.9716	0.979	**6.92E−4**	3.160
217	↑	(R)-1-Aminopropan-2-Ol	Amino acid metabolism	3.2209	1.687	**7.01E−4**	3.154
218	↑	Asparaginyl-Lysine	Lysine degradation	2.4294	1.281	**7.07E−4**	3.150
219	↓	Tyrosinamide	Amino acid metabolism	0.3083	-1.698	**7.16E−4**	3.145
220	↓	Deisopropylatrazine	Amino acid metabolism	0.4513	-1.148	**8.00E−4**	3.097
221	↑	Valyl-Aspartate	Protein digestion & absorption	2.0640	1.046	**8.48E−4**	3.071
222	↑	Glycine	Glycine, serine & threonine metabolism	2.9026	1.537	**8.55E−4**	3.068
223	↑	(2R,3R)-3-Methylornithine	Arginine & proline metabolism	2.0082	1.006	**8.56E−4**	3.068
224	↑	Isomer 1 of 2-Hydroxy-6-oxonona-2,4-diene-1,9-dioic acid	Amino acid metabolism	1.6294	0.704	**9.14E−4**	3.039
225	↑	Proline	Arginine & proline metabolism	2.0415	1.030	**9.32E−4**	3.030
226	↓	Leucyl-Glycine	Glycine, serine & threonine metabolism	0.4933	-1.020	1.04E−3	2.985
227	↑	Arginyl-Serine	Glycine, serine & threonine metabolism	3.1932	1.675	1.08E−3	2.966
228	↑	Leucyl-Aspartate	Protein digestion & absorption	2.7099	1.438	1.11E−3	2.956
229	↑	Tetrahydrobiopterin/L-Threo-Tetrahydrobiopterin	Protein digestion & absorption	2.7956	1.483	1.11E−3	2.954
230	↑	N2'-Acetyl-3'-Hydroxy-L-kynurenine	Tryptophan metabolism	1.5505	0.633	1.13E−3	2.946
231	↑	N6-Acetyl-Lysine	Lysine degradation	1.8458	0.884	1.16E−3	2.935
232	↓	Coutaric acid	Amino acid metabolism	0.4760	-1.071	1.24E−3	2.906

Rank	Dir.	Metabolite	Pathway	FC	log_2(FC)	p-value	$-log_{10}$(p)
233	↓	Spermine dialdehyde	Amino acid metabolism	0.4724	-1.082	1.26E−3	2.900
234	↓	5-Hydroxy-N-Formylkynurenine	Tryptophan metabolism	0.3613	-1.469	1.26E−3	2.900
235	↑	Arginyl-Valine	Valine, leucine & isoleucine metabolism	1.9357	0.953	1.39E−3	2.856
236	↑	Threoninyl-Tryptophan	Tryptophan metabolism	2.1779	1.123	1.42E−3	2.847
237	↑	Arginyl-Tyrosine	Protein digestion & absorption	2.2438	1.166	1.50E−3	2.824
238	↑	Alanyl-Asparagine	Alanine, aspartate & glutamate metabolism	1.7789	0.831	1.62E−3	2.789
239	↑	5-[[(4,7-Dihydroxy-2-Oxo-2H-1-Benzopyran-3-Yl)Amino]Carbonyl]-4-Methyl-1H-Pyrrole-3-Carboxylic Acid	Protein digestion & absorption	4.9589	2.310	2.05E−3	2.689
240	↓	Caffeic acid	Amino acid metabolism	0.6375	-0.649	2.15E−3	2.668
241	↑	Formyl-5-Hydroxykynurenamine	Tryptophan metabolism	2.7751	1.472	2.28E−3	2.642
242	↑	5-Aminopentanamide	Amino acid metabolism	2.0645	1.046	2.31E−3	2.637
243	↑	2'-Aminoacetophenone	Amino acid metabolism	2.0931	1.066	2.41E−3	2.618
244	↑	3-Amino-4-hydroxybenzoic acid	Amino acid metabolism	2.9137	1.543	2.57E−3	2.591
245	↑	Alanyl-Glutamic Acid	Protein digestion & absorption	2.1635	1.113	2.67E−3	2.573
246	↑	2,8-Dihydroxyadenine	Amino acid metabolism	1.7346	0.795	2.85E−3	2.545
247	↓	7-Methylguanine	Protein digestion & absorption	0.5631	-0.829	3.04E−3	2.517
248	↑	Uridine	Pyrimidine metabolism	2.5420	1.346	3.09E−3	2.510
249	↑	Isomer 1 of Prolyl-Alanine	Alanine, aspartate & glutamate metabolism	1.6691	0.739	3.23E−3	2.490
250	↑	Glutaminyl-Tryptophan	Tryptophan metabolism	2.6093	1.384	3.29E−3	2.482
251	↓	Serine	Glycine, serine & threonine metabolism	0.5243	-0.932	3.33E−3	2.478
252	↑	Isoleucyl-Hydroxyproline	Arginine & proline metabolism	1.7220	0.784	3.49E−3	2.457
253	↑	4-Nitrocatechol	Amino acid metabolism	1.7137	0.777	3.57E−3	2.447
254	↓	Isoglutamine	Alanine, aspartate & glutamate metabolism	0.1452	-2.784	3.58E−3	2.446
255	↓	2-Aminoadipate 6-Semialdehyde	Amino acid metabolism	0.4297	-1.219	3.93E−3	2.406
256	↑	Isomer 1 of (2R,3R,4R)-2-Amino-4-hydroxy-3-methylpentanoic acid	Amino acid metabolism	1.6829	0.751	4.10E−3	2.387
257	↓	8-Aminooctanoic acid	Amino acid metabolism	0.6308	-0.665	4.52E−3	2.345

Rank	Dir.	Metabolite	Pathway	FC	log_2(FC)	p-value	$-log_{10}$(p)
258	↑	Taurine	Taurine & hypotaurine metabolism	1.7647	0.819	4.63E−3	2.334
259	↑	2-Deoxystreptamine	Amino acid metabolism	1.6098	0.687	4.67E−3	2.330
260	↓	Isoleucylproline	Arginine & proline metabolism	0.5643	-0.825	4.70E−3	2.328
261	↓	Alanyl-Proline	Arginine & proline metabolism	0.1884	-2.408	4.70E−3	2.328
262	↑	Prolyl-Proline	Arginine & proline metabolism	3.5081	1.811	4.82E−3	2.317
263	↑	Glutaminyl-Serine/Seryl-Glutamine	Alanine, aspartate & glutamate metabolism	2.0053	1.004	5.91E−3	2.229
264	↑	Valyl-Leucine	Valine, leucine & isoleucine metabolism	1.6011	0.679	5.93E−3	2.227
265	↑	4-Methylene-L-glutamic acid	Protein digestion & absorption	2.2571	1.174	5.94E−3	2.227
266	↑	Glutamylproline	Arginine & proline metabolism	1.6944	0.761	6.02E−3	2.220
267	↑	L-Pyridosine	Amino acid metabolism	1.6634	0.734	6.08E−3	2.216
268	↑	Glutamyl-Glutamate	Alanine, aspartate & glutamate metabolism	2.2924	1.197	6.13E−3	2.212
269	↑	4-Hydroxy-4-methylglutamic acid	Amino acid metabolism	2.6301	1.395	6.24E−3	2.205
270	↑	Arginyl-Histidine	Histidine metabolism	1.8009	0.849	6.27E−3	2.203
271	↓	Cyclic pyranopterin monophosphate	Amino acid metabolism	0.4467	-1.163	6.40E−3	2.194
272	↑	Threo-3-Methylaspartic Acid	Alanine, aspartate & glutamate metabolism	1.7990	0.847	6.99E−3	2.156
273	↓	Tyrosyl-Valine	Valine, leucine & isoleucine metabolism	0.2844	-1.814	7.41E−3	2.130
274	↑	Alanyl-Tyrosine	Protein digestion & absorption	1.5482	0.631	7.96E−3	2.099
275	↑	Iminodiacetic acid	Amino acid metabolism	1.6921	0.759	8.12E−3	2.090
276	↑	Hemigossypol	Amino acid metabolism	1.6292	0.704	8.23E−3	2.085
277	↓	Allysine	Lysine degradation	0.4694	-1.091	8.86E−3	2.053
278	↑	Isoleucyl-Alanine	Alanine, aspartate & glutamate metabolism	1.5413	0.624	9.46E−3	2.024
279	↑	Dtdp-3-Amino-3,6-Dideoxy-D-Glucose/Dtdp-3-Amino-3,6-Dideoxy-Alpha-D-Galactopyranose	Protein digestion & absorption	2.1049	1.074	9.77E−3	2.010
280	↑	Alanine	Alanine, aspartate & glutamate metabolism	2.0510	1.036	1.06E−2	1.975
281	↓	3-Hydroxykynurenamine O-sulfate	Amino acid metabolism	0.4831	-1.050	1.13E−2	1.946
282	↓	Isomer 1 of 1-Methylguanosine	Purine metabolism	0.5071	-0.980	1.18E−2	1.928

Rank	Dir.	Metabolite	Pathway	FC	log_2(FC)	p-value	$-log_{10}$(p)
283	↑	Methylcysteine	Cysteine & methionine metabolism	2.2117	1.145	1.21E−2	1.919
284	↑	5-Hydroxykynurenamine	Tryptophan metabolism	1.6380	0.712	1.24E−2	1.908
285	↑	Seryl-Serine	Glycine, serine & threonine metabolism	1.7313	0.792	1.28E−2	1.893
286	↓	Ascorbic acid	Amino acid metabolism	0.6253	-0.677	1.29E−2	1.888
287	↑	Isoleucyl-Lysine	Lysine degradation	2.0401	1.029	1.36E−2	1.866
288	↑	Allocystathionine	Amino acid metabolism	1.8702	0.903	1.39E−2	1.858
289	↑	Thiamine	Amino acid metabolism	1.8482	0.886	1.48E−2	1.829
290	↑	5,6-Dihydroxyindole	Amino acid metabolism	1.5917	0.671	1.61E−2	1.793
291	↑	N-Acetyl-Dihydrofolic Acid	Amino acid metabolism	2.0369	1.026	1.65E−2	1.783
292	↓	3-Amino-2-Piperidone	Amino acid metabolism	0.5618	-0.832	1.65E−2	1.783
293	↓	Phenylalanyl-Glycine	Glycine, serine & threonine metabolism	0.3277	-1.610	1.78E−2	1.750
294	↑	Valine	Valine, leucine & isoleucine metabolism	1.5890	0.668	1.80E−2	1.745
295	↑	N-Acetyl-N-Methylserotonin	Tryptophan metabolism	1.5527	0.635	2.12E−2	1.674
296	↑	Lysyl-Asparagine/Asparaginyl-Lysine	Alanine, aspartate & glutamate metabolism	1.8907	0.919	2.13E−2	1.671
297	↑	Glycyl-Tyrosine	Protein digestion & absorption	1.5877	0.667	2.23E−2	1.652
298	↑	Valyl-Arginine	Protein digestion & absorption	1.8056	0.852	2.29E−2	1.640
299	↑	Kynurenine	Tryptophan metabolism	1.9433	0.958	2.40E−2	1.619
300	↓	3,4-Dihydroxyphenylacetaldehyde 4-O-glucuronide	Amino acid metabolism	0.5994	-0.738	2.42E−2	1.616
301	↓	Isomer 1 of Lysyl-Proline	Arginine & proline metabolism	0.4735	-1.078	2.43E−2	1.614
302	↓	Phenol	Amino acid metabolism	0.6291	-0.669	2.47E−2	1.608
303	↑	Chlorohydroquinone	Amino acid metabolism	2.9579	1.564	2.52E−2	1.599
304	↑	Isomer 1 of 4-Hydroxystyrene	Amino acid metabolism	1.6971	0.763	2.67E−2	1.573
305	↑	Isomer 1 of Lysyl-Glutamate	Alanine, aspartate & glutamate metabolism	2.1294	1.090	2.70E−2	1.568
306	↓	Cysteinyldopa	Protein digestion & absorption	0.6643	-0.590	2.77E−2	1.558
307	↓	2-Deoxy-scyllo-inosamine	Amino acid metabolism	0.6652	-0.588	2.80E−2	1.554
308	↓	Isoleucyl-Methionine	Cysteine & methionine metabolism	0.6296	-0.667	2.88E−2	1.541

Rank	Dir.	Metabolite	Pathway	FC	$\log_2$(FC)	p-value	$-\log_{10}$(p)
309	↑	Glutaminyl-Phenylalanine	Phenylalanine metabolism	1.8164	0.861	3.14E−2	1.504
310	↑	p-Coumaroylagmatine	Amino acid metabolism	1.5355	0.619	3.24E−2	1.489
311	↓	3-Sulfocatechol	Amino acid metabolism	0.4702	-1.089	3.25E−2	1.488
312	↑	Aminoadipic Acid	Amino acid metabolism	1.8065	0.853	3.40E−2	1.468
313	↑	Isoleucyl-Isoleucine	Valine, leucine & isoleucine metabolism	1.6522	0.724	4.04E−2	1.394
314	↓	Cysteinyl-Tryptophan	Tryptophan metabolism	0.6238	-0.681	4.28E−2	1.368
315	↓	3,4-Dihydroxyphenylacetaldehyde 3-O-glucuronide	Amino acid metabolism	0.6129	-0.706	4.46E−2	1.351
316	↓	Lysyl-Tryptophan	Tryptophan metabolism	0.6489	-0.624	4.81E−2	1.317

Significance criteria: $p < 0.05$ (Welch's two-tailed t-test) AND $|FC| > 1.5$. Metabolites ranked by ascending p-value. FC = fold change of first group relative to second. ↑ = increased; ↓ = decreased. $\log_2$(FC) and $-\log_{10}$(p) provided for volcano plot reference. Pathway annotations based on KEGG Bos taurus metabolic library. Source data: metaboan2.csv (n = 10 per group; 474 metabolites after Tier 3 exclusions).
