# Supplementary material for: The Dry Secretion Metabolome: LC-MS Profiling Distinguishes Subclinical Mastitis from Healthy Udder Quarters Across the Dry Period in Dairy Cows
Source: Vet Sci. 2026 Apr 2;13(4):345. doi: 10.3390/vetsci13040345 (PMC13120032; doi:10.3390/vetsci13040345)
Supplement: Supplementary file 1 [file vetsci-13-00345-s001.zip › Supplementary_Figures.pdf]

Supplementary Figures

The Dry Secretion Metabolome: LC-MS Profiling Distinguishes Subclinical Mastitis from Healthy Udder Quarters Across the Dry Period in Dairy Cows

High Performance Chemical Isotope Labeling LC-MS (TMIC028P)

Figure S1: Permutation Test Validation of PLS-DA Models Across Four Pairwise Comparisons (200 Permutations)

Figure S2: ROC Curves for Leave-One-Out Cross-Validated PLS-DA Classification Models Across Four Pairwise Comparisons

Figure S3: Radar Chart of Significantly Altered Metabolites per KEGG Pathway Across Four Pairwise Comparisons

Figure S4: Waterfall Plots of Significantly Altered Metabolites Ranked by $\log_2$(Fold Change), Color-Coded by KEGG Pathway

Abbreviations: SCM = Subclinical Mastitis; H = Healthy; D2 = Day 2 dry-off; D21 = Day 21 dry period. AUC = Area Under the Curve; ROC = Receiver Operating Characteristic; VIP = Variable Importance in Projection; PLS-DA = Partial Least Squares Discriminant Analysis; FC = Fold Change; FDR = False Discovery Rate (Benjamini-Hochberg). Pathway annotations from KEGG *Bos taurus* metabolic library via MetaboAnalyst. n = 10 cows per group; 474 total metabolites after exclusion of 8 Tier 3 identifications.

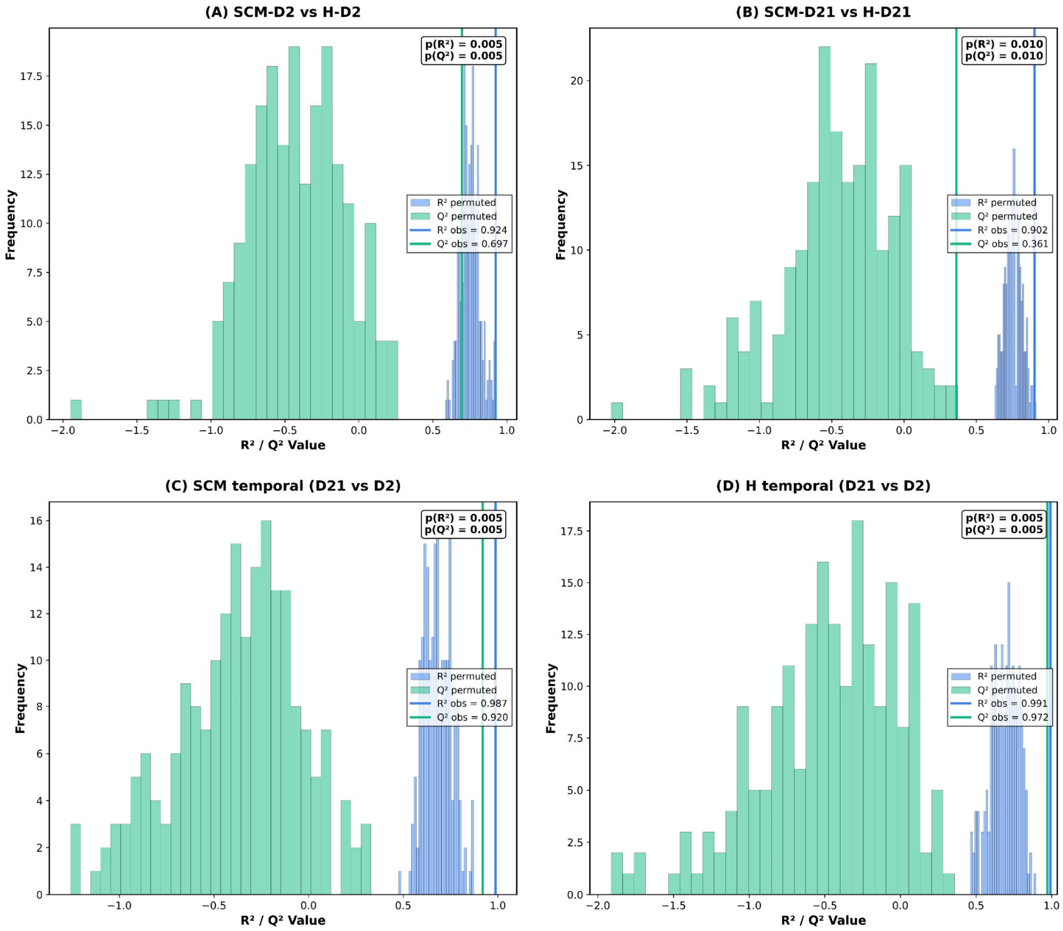

Figure S1. Permutation tests for PLS-DA model validation (200 permutations per comparison). Histograms show the distribution of R2 (blue) and Q2 (green) values obtained by fitting PLS-DA models to randomly permuted group labels, representing the null hypothesis of no systematic difference between groups. Vertical lines indicate the observed R2 and Q2 from the true (unpermuted) model. (A) SCM-D2 vs H-D2: observed R2 = 0.924, Q2 = 0.697; both significantly exceeded all permuted values (p = 0.005). (B) SCM-D21 vs H-D21: observed R2 = 0.902, Q2 = 0.361; significant at p = 0.010, although the lower Q2 reflects reduced predictive ability consistent with the attenuated disease signature. (C) SCM temporal (D21 vs D2): observed R2 = 0.987, Q2 = 0.920 (p = 0.005). (D) H temporal (D21 vs D2): observed R2 = 0.991, Q2 = 0.972 (p = 0.005). In all four comparisons, the observed statistics fell outside the null distribution, confirming that group separations are not attributable to chance. Two-component PLS-DA models; n = 10 per group; 482 metabolites; auto-scaled data.

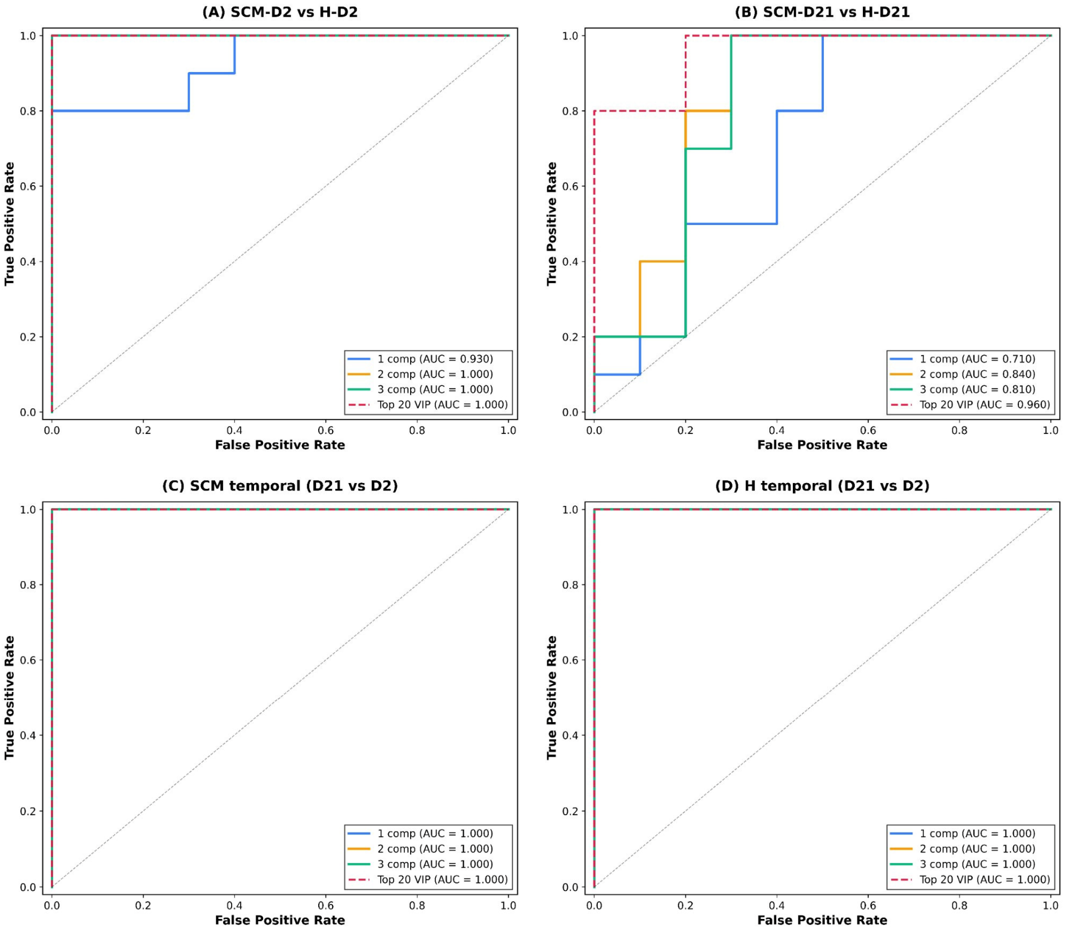

Figure S2. Receiver operating characteristic (ROC) curves for PLS-DA classification models across four pairwise comparisons. Curves were generated from leave-one-out cross-validated predicted scores to avoid overfitting. (A) SCM-D2 vs H-D2: AUC = 1.000 with 1–3 PLS components; the top-20-VIP reduced model (dashed rose) also achieved AUC = 1.000. (B) SCM-D21 vs H-D21: AUC = 0.810 (3 components), reflecting the weaker metabolic separation at Day 21. (C) SCM temporal: AUC = 1.000 across all model configurations. (D) H temporal: AUC = 1.000 across all configurations. Diagonal line: random classifier (AUC = 0.5). Solid lines: models using all 474 metabolites with 1, 2, or 3 PLS components. Dashed rose line: parsimonious model using only the top 20 VIP-ranked metabolites (2 components). The comparable performance of the reduced model in (A), (C), and (D) suggests that a compact biomarker panel captures the essential discriminatory information. n = 10 per group.

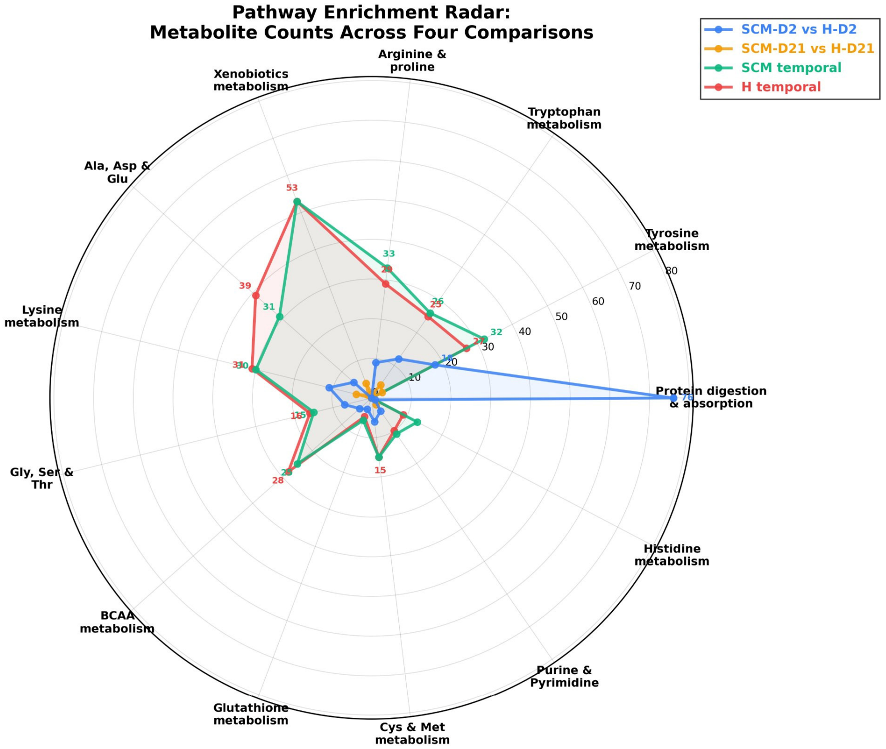

Figure X. Radar chart of metabolite counts per KEGG pathway across four pairwise comparisons. Each spoke represents one pathway; distance from center indicates number of significant metabolites (p < 0.05). Note the dramatic protein digestion spike in the D2 health comparison (dipeptides) and the parallel xenobiotics peaks in both temporal comparisons. Health comparisons at D21 (amber) show uniformly low counts, confirming attenuation of the SCM metabolic signature.

Figure S3. Radar chart of metabolite counts per KEGG pathway across four pairwise comparisons. Each spoke represents one metabolic pathway; distance from the centre indicates the number of significantly altered metabolites ($p < 0.05$). Lines connect values for each comparison: SCM-D2 vs H-D2 (blue), SCM-D21 vs H-D21 (amber), SCM temporal (green), H temporal (red). The protein digestion and absorption spike (76 metabolites, blue) reflect the dipeptide-dominated SCM signature. Both temporal comparisons show parallel peaks in xenobiotics metabolism (53 each). The near-collapse of the amber polygon confirms attenuation of health-associated differences by Day 21. This figure provides an alternative visualisation of the data presented in Figure 8. n = 10 per group; 474 total metabolites.

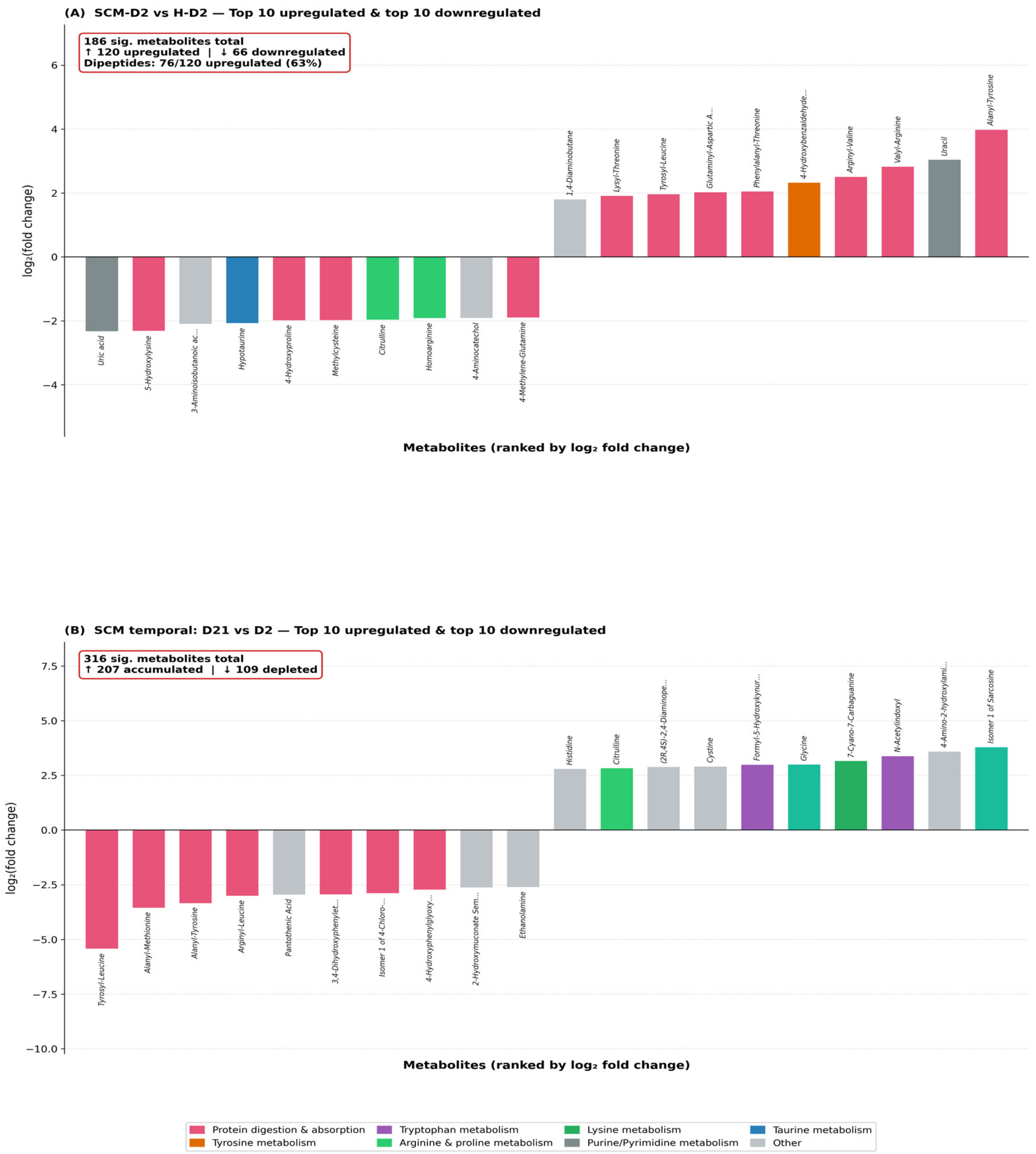

Figure S4. Waterfall plots of the top 10 most upregulated and top 10 most downregulated metabolites per comparison, ranked by $\log_2$(fold change) and colour-coded by KEGG pathway. Only the 20 most extreme metabolites are shown for clarity; total significant counts are indicated in each panel. (A) SCM-D2 vs H-D2: 186 significant metabolites total; the upregulated tail is dominated by dipeptides (rose), with 76 of 120 upregulated metabolites (63%) classified under protein digestion and absorption, reflecting neutrophil-driven proteolysis in SCM quarters. (B) SCM temporal (D21 vs D2): 316 significant metabolites total, with 207 accumulated and 109 depleted. Legend shows only pathways represented among the top 20 metabolites in each panel (n = 10 per group; CIL-LC-MS platform).
